# Validation of an Independent Web-Based Tool for Measuring Visual Acuity and Refractive Error (the Manifest versus Online Refractive Evaluation Trial): Prospective Open-Label Noninferiority Clinical Trial.

**DOI:** 10.2196/14808

**Published:** 2019-11-08

**Authors:** Robert P L Wisse, Marc B Muijzer, Francesco Cassano, Daniel A Godefrooij, Yves F D M Prevoo, Nienke Soeters

**Affiliations:** 1 Utrecht Cornea Research Group Ophthalmology Department University Medical Center Utrecht Utrecht Netherlands; 2 Easee BV Amsterdam Netherlands

**Keywords:** digital refraction, easee, telemedicine, medical informatics, refractive error

## Abstract

**Background:**

Digital tools provide a unique opportunity to increase access to eye care. We developed a Web-based test that measures visual acuity and both spherical and cylindrical refractive errors. This test is Conformité Européenne marked and available on the Easee website. The purpose of this study was to compare the efficacy of this Web-based tool with traditional subjective manifest refraction in a prospective open-label noninferiority clinical trial.

**Objective:**

The aim of this study was to evaluate the outcome of a Web-based refraction compared with a manifest refraction (golden standard).

**Methods:**

Healthy volunteers from 18 to 40 years of age, with a refraction error between –6 and +4 diopter (D), were eligible. Each participant performed the Web-based test, and the reference test was performed by an optometrist. An absolute difference in refractive error of <0.5 D was considered noninferior. Reliability was assessed by using an intraclass correlation coefficient (ICC). Both uncorrected and corrected visual acuity were measured.

**Results:**

A total of 200 eyes in 100 healthy volunteers were examined. The Web-based assessment of refractive error had excellent correlation with the reference test (ICC=0.92) and was considered noninferior to the reference test. Uncorrected visual acuity was similar with the Web-based test and the reference test (*P*=.21). Visual acuity was significantly improved using the prescription obtained by using the Web-based tool (*P*<.01). The Web-based test provided the best results in participants with mild myopia (ie, <3 D), with a mean difference of 0.02 (SD 0.49) D (*P*=.48) and yielding a corrected visual acuity of >1.0 in 90% (n=77) of participants.

**Conclusions:**

Our results indicate that Web-based eye testing is a valid and safe method for measuring visual acuity and refractive error in healthy eyes, particularly for mild myopia. This tool can be used for screening purposes, and it is an easily accessible alternative to the subjective manifest refraction test.

**Trial Registration:**

Clinicaltrials.gov NCT03313921; https://clinicaltrials.gov/ct2/show/NCT03313921.

## Introduction

### Background

**Figure 1:**
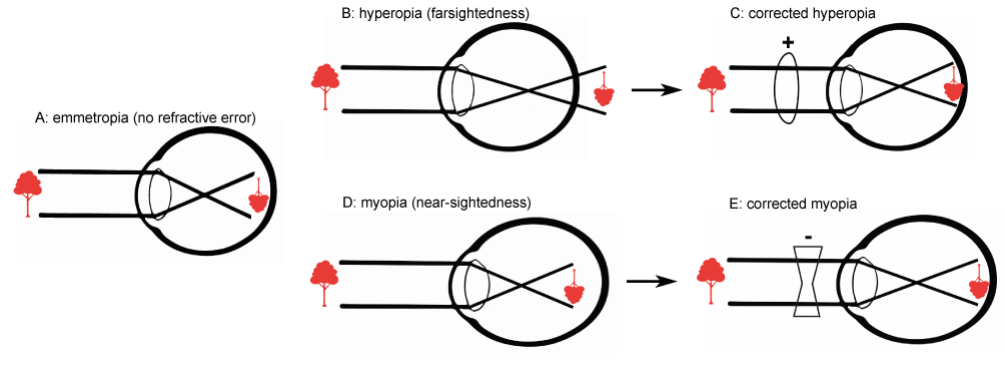
Optics of the eye. A: With no refractive error, the image is focused properly on the retina, providing perfect uncorrected visual acuity. B and D: In hyperopia (far-sightedness; B) and myopia (nearsightedness; D) the image falls either behind or in front of the retina, respectively. C and E: Lenses can be used to re-focus the image on the retina, restoring visual acuity.

Globally, approximately 60% of individuals require a visual aid, such as spectacles or contact lenses, for proper visual acuity [1,2]. Moreover, studies have shown that the incidence of myopia (ie, nearsightedness) is increasing steadily because of higher literacy rates and increasing urbanization [3,4]. The World Health Organization has reported that these so-called refractive errors—if not corrected—represent the principal cause of visual impairment [5]. Strikingly, nearly 50% of preventable visual impairment is caused by the use of inappropriate spectacles or lenses, with severe economic implications [3,6]. Even in countries with readily accessible health care services, this rate remains unacceptably high, and calls for a new way of thinking about how visual aids are prescribed [2]. To improve eye health in our global population, we need access to reliable, affordable tools for measuring refractive error. In today’s digital era, the ability to digitize the refractive exam is the logical solution. Indeed, many examples are available, supporting the robust potential of digital medicine, as well as the cultural and operational hurdles that must be overcome to bring medicine into the data-driven age [7,8]. To increase access to refractive testing, the Dutch company Easee BV in Amsterdam, the Netherlands, developed an algorithm-based Web-based tool that measures the refractive state of the eye by using a smartphone and computer screen. This tool is *Conformité Européenne* (CE) marked, complies with all required International Organization for Standardization (ISO) standards, and is currently available on the Web. Notwithstanding the apparent accessibility of this service, its validity and safety need to be studied and reported as a means to keep developers accountable for their health innovations. Traditionally, refractive error is measured by an eye care professional, in which trial lenses of various corrective strength are tested on the basis of the patient’s responses, whereas a letter chart is used to assess the resulting visual acuity. The outcome of this test can include emmetropia (no refractive error), hyperopia (farsightedness), or myopia (nearsightedness), as well as astigmatism (a cylindrical error; see Figure 1). This so-called subjective manifest refraction test is currently considered the gold standard [9,10]. However, the quality of the measurement can depend upon a variety of factors, including the availability of the necessary equipment, a suitable environment for testing, the patient’s ability and willingness to cooperate with the examiner, and the examiner’s experience and training. Alternatively, refraction can be measured by using an automated approach, for example, with an automated refractor [11,12], an aberrometer [13], or adaptive optics [14]. Nevertheless, both the subjective and automated techniques require expensive medical equipment and qualified personnel, which can limit their availability and accessibility. Current developments in new refractive methods are summarized the Research in Context panel (Multimedia Appendix 1).

### Objectives

In this paper, we present the results of the Manifest versus Online Refraction Evaluation (MORE) trial, a study designed to validate this Web-based refractive assessment by comparing the outcome between the Web-based test and the subjective manifest refraction test, focusing on corrected visual acuity achieved by using the prescription obtained from the Web-based test.

## Methods

### Study Design and Recruitment

Data were prospectively collected in the open-label single-center noninferiority MORE trial, performed at the University Medical Center Utrecht in Utrecht, the Netherlands. The participants were healthy volunteers, from 18 to 40 years of age, with no history of eye disease or current evidence of eye disease. We excluded subjects whose refractive error was worse than –6 diopter (D; for myopia) or +4 D (for hyperopia) and subjects who had diabetes, were pregnant or lactated, or were unable to perform the Web-based test. All participants provided written informed consent. All subjects underwent 3 consecutive tests designed to determine the refractive state of both eyes in the following order. First, the subject performed the index test using the Web-based refractive assessment tool with the Easee algorithm. Second, the refractive error was measured using autorefraction (Topcon RM 8800). Finally, an optometrist performed the reference test (manifest subjective refraction). The subject was blinded for the outcome of all tests. The subject’s uncorrected distance visual acuity (UDVA) was recorded using a traditional Early Treatment Diabetic Retinopathy Study visual acuity chart and the Easee Web-based visual acuity test. Corrected distance visual acuity (CDVA) was measured using correction on the basis of the results of the manifest and Web-based refraction tests. Visual acuity was tested in accordance with ISO 8596, with regard to optotypes and room illumination [15]. The projected optotypes were randomized to mitigate any possible test-retest effect. Clinical agreement between manifest subjective refraction and autorefraction is generally considered excellent [10]; therefore, CDVA was not assessed using the results of the autorefraction test. The following data were recorded for each participant/eye: age, gender, laterality, medical history, previous prescription (if known), use of spectacles or contact lenses, UDVA, CDVA, and refractive outcome, including spherical and cylindrical power (in D) and axis (in degrees), which were converted into power vectors, using a Fourier analysis [16,17]. All procedures were performed in accordance with the Declaration of Helsinki, local and national laws regarding research (ie, the Act on Scientific Research Involving Humans), European directives with respect to privacy (General Data Protection Regulation 2016/679) and medical devices (Medical Device Regulation 2017/745), and the 2015 Standards for Reporting Diagnostic Accuracy Studies [18]. The study protocol was approved by our institution’s Ethics Review Board (METC number: 17-524), and it was registered on the Web at clinicaltrials.gov (number: NCT03313921) and CCMO.nl (number: NL61478.041.17).

### Information Regarding the Web-Based Tool

**Figure 2:**
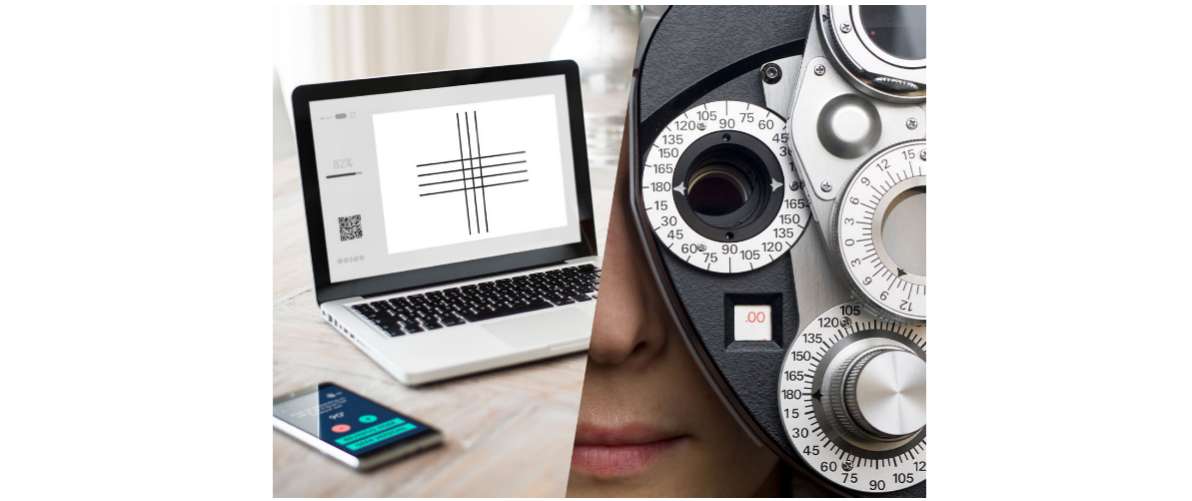
An impression of the online refraction exam and its comparator the manifest refraction.

The Web-based tool for measuring refractive error uses a smartphone and a standard computer screen (Figure 2). This commercially available test is available via the website of Easee, and it uses the same algorithm described in this study; an 80-second video tutorial is also available at the website, and a clinical test flow is provided in the supplementary files. In brief, a smartphone functions as a remote control by which the user submits input from a distance of 3 m or 1.5 m to a computer screen that displays the Web-based test. Audio instructions (currently available in Dutch, English, and German) guide the user through the test, during which both eyes are tested consecutively. During the test, the user is presented a sequence of images and optotypes that the user must correctly identify, in addition to various grate sizes and astigmatism dials used to assess the cylindrical error. Any visual acuity below 1.0 (ie, worse than 20/20) is considered to be because of a refractive error. The direction of the refractive error (ie, hyperopia + or myopia –) is based on an adapted red/green duochrome test [19] and a questionnaire designed to discern between nearsightedness and farsightedness. A version of the Easee Web tool was custom built for this clinical trial in which only anonymized data were captured by the tool. The Web tool is classified as a class 1 medical device, which is in accordance with Medical Device Regulation 2017/745, and the software is classified as class A, which is in accordance with IEC 62304:2014.

### Statistical Analysis

The primary study outcome was refractive error, measured using the Web-based tool, and refractive error compared with a subjective manifest refraction and autorefraction. Specifically, we analyzed the sign of the refractive error (+/–), spherical power, cylindrical power, and axis, which were converted into power vectors by using a Fourier analysis [16,17]. An intraclass correlation coefficient (ICC) among the various methods was also calculated [20]. Autorefraction measurements were primarily used to provide a context for the level of correlation between a subjective manifest refraction and the Web-based tool. The secondary study outcomes included UDVA and CDVA, measured using the prescriptions obtained using the Web-based tool and a subjective manifest refraction. UDVA and CDVA were converted to logarithm of the minimum angle of resolution (logMAR) values for statistical analysis. Groups were compared by using the 2-tailed paired Student’s *t* test, or Pearson chi-square test. In addition, a multivariable analysis, using a generalized estimates equation, was used to correct for bilaterality (both eyes of the same patient included), age, and sex. Differences with a *P* value <.05 were considered statistically significant. A stratification in outcomes was defined in the study protocol for myopic and hyperopic results, as the subjective measurement of these distinct refractive states is prone to particular errors. A difference in spherical equivalent (SEQ) >0.5 D between the 3 refraction methods was considered to reflect a clinically significant difference; thus, this constituted the threshold for noninferiority [21,22]. The power calculation was based on an intraclass correlation for the 3 different refraction methods, using the following formula in R: Sample size(p=0.70,p0=0,k=3,alpha=(0.05/12),tails=2,power=0.80, by=”p”, step=0.025).

Initially, 50 healthy subjects with 100 healthy eyes were scheduled to enter the study. An interim analysis indicated that the algorithm yielded more outlier measurements than anticipated, thereby skewing the results. A second-generation algorithm was therefore developed, using the clinical data acquired to date. An extension was requested for the trial, and it was granted by our institution’s Ethics Review Board. Any incomplete data were imputed, except when it concerned missing data from the primary outcome. Data were analyzed using IBM SPSS v25.0 (IBM).

## Results

### Description of the Study Population

A total of 200 eyes from 100 healthy subjects were included in the study; 1 eye was excluded from analysis because of amblyopia (lazy eye). All subjects were enrolled in the study between December 28, 2017 and January 28, 2019. The clinical characteristics of the participants are summarized in Table 1. Most of the subjects (62%, n=62) were regular users of spectacles or contact lenses. A total of 4 subjects reported receiving previous treatment for an ophthalmic condition; in all 4 cases, the ophthalmic condition resolved without sequelae. A total of 11 subjects reported ocular complaints at the time of the measurements; 8 subjects reported blurred vision, and 3 subjects reported other complaints, such as floaters and dry eyes. The mean test duration was 22 (SD 10) min (range 5-58). No adverse events or complications were recorded during the trial.

**Table 1:** Clinical characteristics of the study population.

Clinical characteristics^a^		Total (N=100)	Web-based test algorithm		*P* value^b^
			1st generation (N=36)	2nd generation (N=64)	
Age (years), mean (SD)		25.4 (4.7)	25.3 (4.2)	25.5 (4.9)	.86
Sex (male), n (%)		47 (47)	23 (64)	24 (38)	.001
**Current use of visual aids, n (%)**		62 (62)	21 (58)	42 (6)	.58
	Spectacles	60 (60)	21 (58)	27 (61)	.88
	Contact lenses	22 (22)	7 (20)	15 (23)	.54
Previous ophthalmic treatment, n (%)		4 (4)	3 (8)	1 (2)	.12
Ocular complaints, n (%)		13 (13)	3 (8)	10 (16)	.34
Medication use, n (%)		13 (13)	2 (6)	11 (17)	.08
**Refractive error^c^** **, n (%)**					
	Emmetropia	16 (8)	4 (6)	12 (9)	N/A^d^
	Mild myopia	119 (60)	40 (56)	79 (62)	N/A
	Severe myopia	32 (16)	13 (18)	19 (15)	N/A
	Hyperopia	32 (16)	15 (21)	17 (13)	N/A
	Total	199	72	127	.24

^a^Except where indicated otherwise, data are presented as n (%).

^b^Calculated using an independent samples Student *t* test or Pearson chi-square test.

^c^Mild myopia was defined as refractive error of –3 D or less; severe myopia was defined as refractive error worse than –3 D. Refractive error was determined on the basis of the spherical equivalent of the manifest refraction value, and it is reported for both eyes separately.

^d^Not applicable.

### Intraclass Correlation Coefficients

As a first measure of the concordance among the 3 methods for assessing refractive error, we measured the ICC. For this analysis, we included only each participant’s right eye and based our calculations on the SEQ. The overall ICC of all 3 measurements was 0.93 (95% CI 0.90-0.96), and the overall ICC for manifest refraction and Web-based refraction was 0.89 (95% CI 0.84-0.93). When only measurements taken with the second-generation algorithm are considered, the ICC improved to 0.92 (95% CI 0.86-0.95). The latter can be considered an excellent agreement [20]. Analyses based on vectors rather than SEQs did not materially alter these findings.

### Reliability of Web-Based Visual Acuity Testing

UDVA was measured by using both the Web-based test and a visual acuity wall chart. UDVA data for the Web-based test were imputed for 6 participants because of a technical recording error. Our analysis revealed that the Web-based test provided UDVA values that were similar to results obtained by using a chart, with mean values of 0.67 (SD 0.33) versus 0.69 (SD 0.37), respectively (LogMAR: 0.33 (SD 0.30) vs 0.39 (SD 0.39); *P*=.21). In addition, the overall ICC of this measurement (for each participant’s right eye only) was 0.89 (95% CI 0.83-0.92).

### Overall Outcome for Measuring Refractive Error With the Web-Based Test Versus the Reference Test

In the entire study group, refractive error between the Web-based refraction test and the reference test differed by –0.18 (SD 0.77) D for participants with myopia and 0.63 (SD 0.89) D for participants with hyperopia. With respect to the participants with myopia, this difference was within our *a priori* threshold for defining noninferiority (see the Multimedia Appendices 2-4). When we analyzed only the participants who were tested using the second-generation algorithm, the difference in SEQ was –0.13 (SD 0.62) D for patients with myopia and 0.50 (SD 0.81) D for patients with hyperopia, both of which are within our threshold for noninferiority margin (see Table 2). Similar results were obtained when we corrected for the confounding factors bilaterality, age, and sex (data not shown).

**Figure 3:**
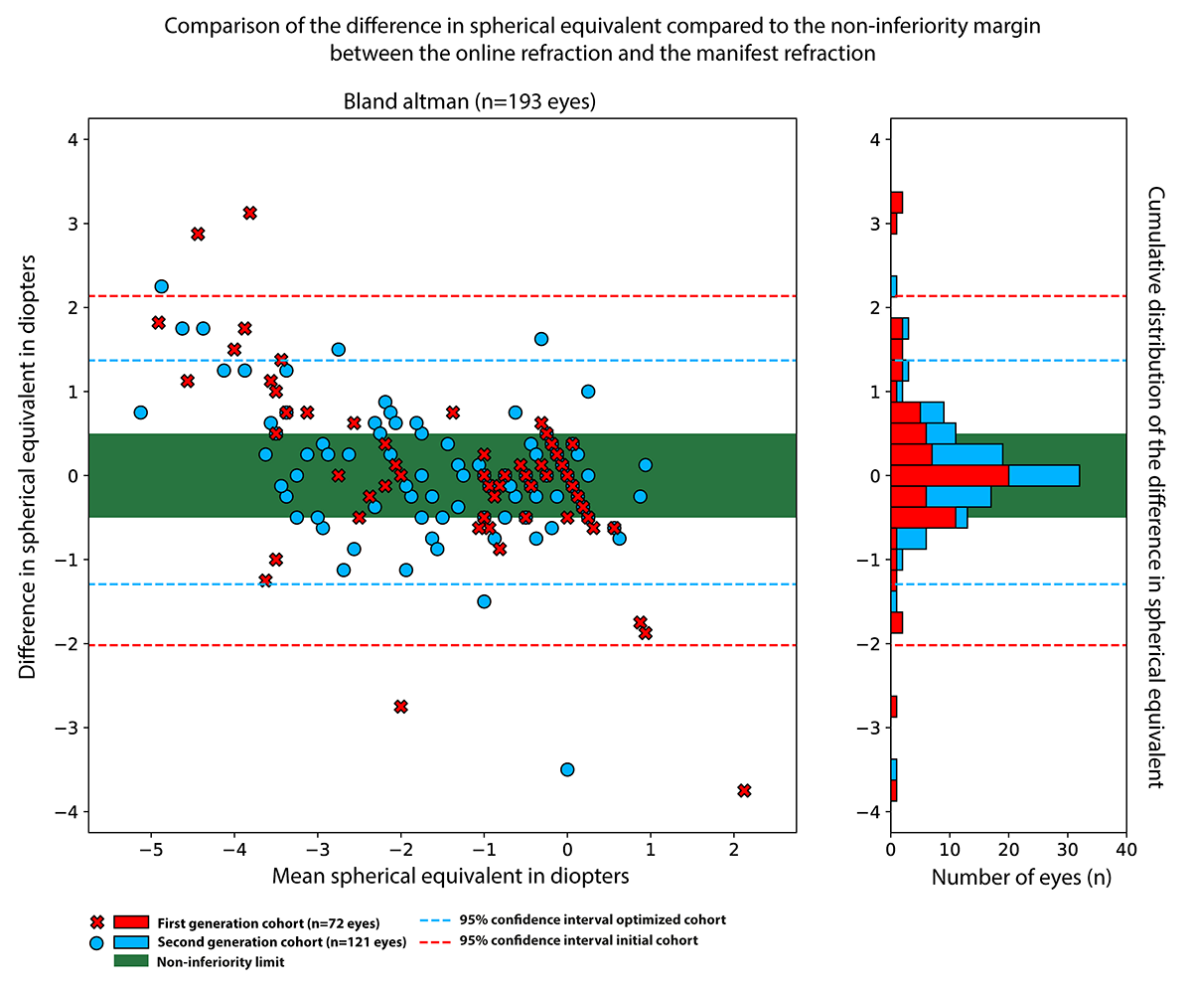
The difference between the refractive error measurement of the first (red) and second generation (blue) online refraction test compared to the outcome of the manifest refraction with respect to the non-inferiority limit (green area) and 95% confidence interval (dashed lines).

**Figure 4:**
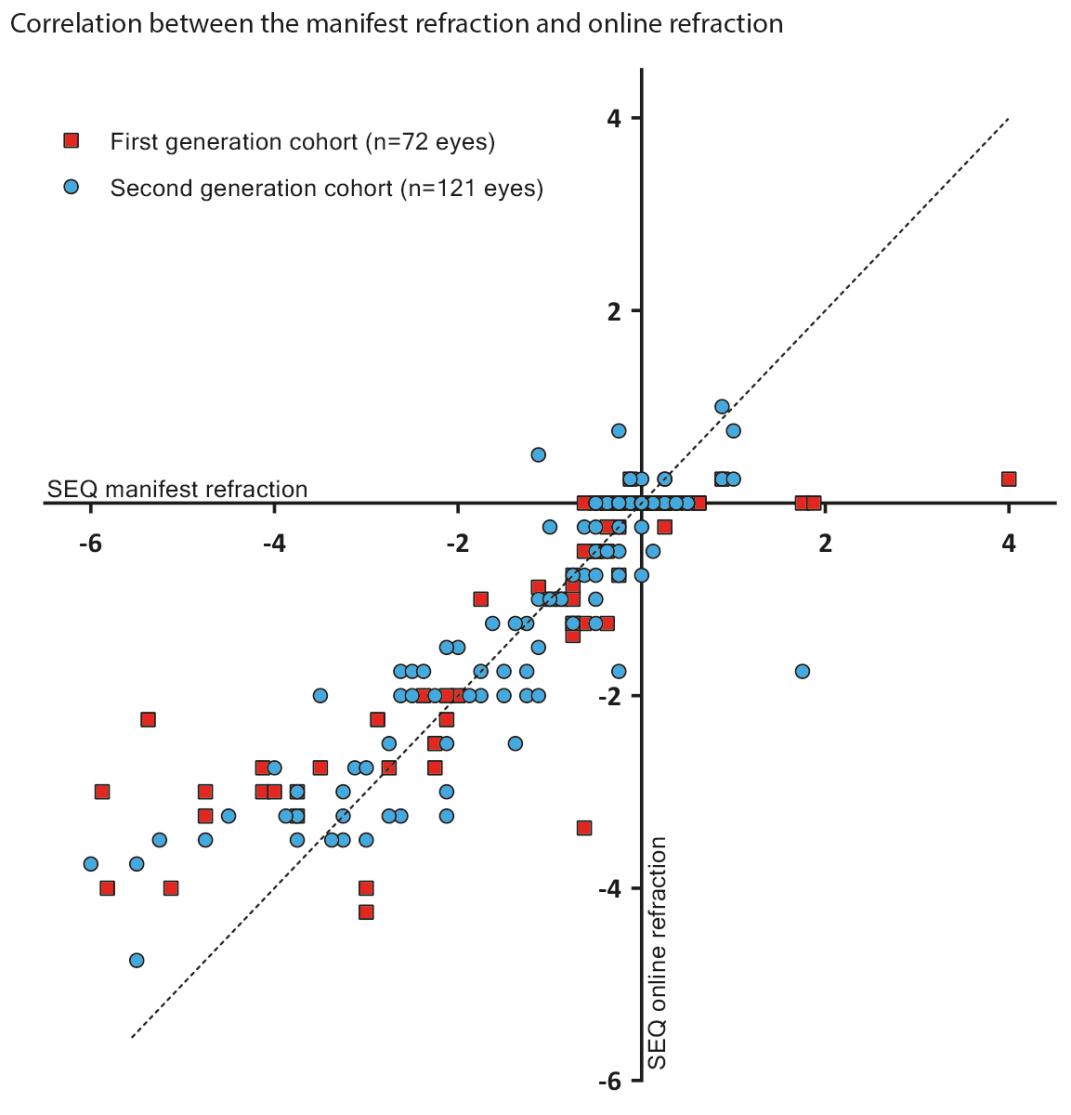
Refractive error measured using the online test was plotted against refractive error measured using manifest refraction; each symbol represents an individual eye measured in a participant who was tested using the first-generation algorithm (red squares) or the second-generation algorithm (blue circles). The 45° dashed line represents an ideal fit. Outliers are identified particularly in the high-myopia group (bottom-left), and these differences are reduced in the second generation cohort. SEQ: spherical equivalent.

Figure 3 shows the difference of the Web-based test compared with the reference test and with respect to the noninferiority limit. As can be observed, a majority of measurements fall within the noninferiority limit, and almost all measurements fall within the 95% CI. In addition, we summarized the distribution of the differences in refractive outcome by using the Web-based test and manifest refraction. Figure 4 shows the individual refractive error data measured for each patient; note that the 6 patients for whom data were missing are not included in these graphs.

**Table 2:** Refractive error and visual acuity measured in the myopic and hyperopic participants (second-generation algorithm; N=121 eyes).

Refractive error and visual acuity		Manifest refraction^a^	Online refraction^a^	Difference	95% CI	*P* value^b^	GEE model^c^	
							Beta value	*P* value^c^
**Emmetropic and myopic eyes (n=104)**								
	Power vector (Diopter)^d^	1.59 (1.50)	1.47 (1.27)	0.12	0.00-0.24	.04	1.11	<.001
	J0 vector (Diopter)	0.09 (0.29)	–0.01 (0.22)	0.10	0.04-0.15	N/A^e^	N/A	N/A
	J45 vector (Diopter)	0.01 (0.17)	–0.01 (0.15)	0.00	–0.04 to 0.03	N/A	N/A	N/A
	Spherical equivalent (Diopter)	–1.54 (–1.52)	–1.41 (1.31)	0.13	–0.25 to –0.01	N/A	N/A	N/A
	Spherical power (Diopter)	–1.31 (1.43)	–1.30 (1.31)	–0.01	–0.13 to 0.10	N/A	N/A	N/A
	Cylindrical power (Diopter)	–0.45 (0.51)	–0.23 (0.47)	–0.22	–0.34 to –0.11	N/A	N/A	N/A
	Cylindrical axis (degrees)	97 (58)	101 (51)	–4	–24 to 16	N/A	N/A	N/A
	CDVA^f^ logarithm of the minimum angle of resolution^g^	–0.14 (0.06)	–0.03 (0.18)	–0.11	–0.14 to –0.08	<.001	.08	.13
	CDVA Snellen^h,i^	1.38 (0.20)	1.15 (0.35)	0.25	0.18-0.32	N/A	N/A	N/A
**Hyperopic eyes (n=17)**								
	Power vector (Diopter)^d^	0.58 (0.45)	0.33 (0.48)	0.25	0.14-0.37	.001	.84	<.001
	J0 vector (Diopter)	0.03 (0.21)	0.00 (0.14)	0.02	–0.11 to 0.15	N/A	N/A	N/A
	J45 vector (Diopter)	–0.02 (0.17)	0.03 (0.09)	0.05	–0.15 to 0.05	N/A	N/A	N/A
	Spherical equivalent (Diopter)	0.53 (0.44)	0.03 (0.57)	0.50	0.11-0.89	N/A	N/A	N/A
	Spherical power (Diopter)	0.71 (0.57)	0.10 (0.58)	0.61	0.16-1.04	N/A	N/A	N/A
	Cylindrical power (Diopter)	–0.35 (0.40)	–0.15 (0.29)	–0.21	–0.38 to –0.04	N/A	N/A	N/A
	Cylindrical axis (degrees)	53 (50)	46 (40)	8	–72 to 88	N/A	N/A	N/A
	CDVA logarithm of the minimum angle of resolution^h^	–0.13 (0.06)	–0.10 (0.11)	–0.03	–0.08 to 0.02	.20	.25	.54
	CDVA Snellen^h,i^	1.37 (0.19)	1.29 (0.28)	0.08	–0.06 to 0.21	N/A	N/A	N/A

^a^Unless otherwise specified, reported as mean (SD).

^b^Paired-sample Student *t* test was performed for predefined primary and secondary outcome parameters only.

^c^Generalized estimates equation model to statistically correct for the inclusion of 2 eyes of one subject, age, and sex.

^d^Spherical and cylindrical power and axes were translated into vectors using Fourier analysis.

^e^Not applicable.

^f^CDVA: corrected distance visual acuity.

^g^Assessed with either the manifest or Web-based achieved correction.

^h^Snellen, decimal visual acuity.

^i^Statistical tests were performed only on predefined parameters (power vector for refraction and logarithm of the minimum angle of resolution for visual acuity).

### Overall Visual Acuity Measured Using the Web-Based Refraction Test and Manifest Refraction

Visual acuity improved significantly using the prescription obtained by using the Web-based refraction test, particularly when using the second-generation algorithm. Specifically, the UDVA was 0.66 (SD 0.41) (LogMAR 0.32 [SD 0.40]), and it improved to a CDVA of 1.17±0.34 (LogMAR –0.04 [SD 0.17]; *P*<.01). Interestingly, we found that CDVA in the hyperopic participants did not differ significantly between the Web-based refraction test (1.29 [SD 0.28], LogMAR –0.10 [SD 0.11]) and the manifest refraction test (1.37 [SD 0.19], LogMAR –0.13 [SD 0.06]; *P*=.20). This is likely because of the accommodation reflex that corrects residual hyperopic refractive errors [1]. A multivariable Generalized Estimating Equations (GEE) analysis did not reveal any major confounders (Table 2).

For myopic participants, the visual acuity (CDVA) differed significantly between the Web-based refraction test (1.15 [SD 0.35]; LogMAR –0.03 [SD 0.18]) and the manifest refraction test (1.38 [SD 0.19]; LogMAR –0.14 [SD 0.06]; *P*<.01). Contrary to hyperopia, even a small an uncorrected residual myopic refractive error will negatively influence distance visual acuity [1]. A multivariable GEE analysis revealed that confounding factors influenced this difference, although with a very small effect size, attributable to the second-generation cohort, harboring relatively more myopic females. Analysis of Web-based test meta-data revealed no clues to a difference in performance of male versus female participants.

### The Ability of the Web-Based Refraction Test to Correctly Distinguish Myopia Versus Hyperopia

In nearly every case, the Web-based refraction was able to correctly determine the participant’s refractive error as either myopia or hyperopia, with the exception of 4 cases. A total of 1 case fell within the noninferiority margin, with a difference of 0.25 D, and CDVA was similar for this eye when corrected with either prescription. The other 3 cases differed to a clinically relevant level: -1.125 versus + 0.50, +0.125 versus –0.50, and +1.75 versus –1.75 for the Web-based and manifest refraction test, respectively. In 195 of 199 cases (98%) of the Web-based assessments, the signation was correct.

### Subgroup Analysis of Participants With Mild Myopia

A majority of eyes in our study were classified as having mild myopia, which is consistent with mild myopia being the most common refractive error in the general population [23]. A subgroup analysis was performed in the eyes, with a refractive error between –3 and 0 D, 91 eyes in total. Using the prescription obtained with the Web-based refraction test, the eyes with mild myopia had a markedly better CDVA compared with the entire group of eyes with myopia (1.22 [SD 0.29]; LogMAR –0.08 [SD 0.11]), with 90% (n=77) of the participants scoring over 1.0. The average difference in refractive error between the 2 tests is now reduced to 0.02 (SD 0.49) D (*P*=.48), and 80% of the Web-based refraction tests were within SD 0.5 D of the reference test. Notwithstanding, the manifest refraction test yielded a slightly better CDVA (1.39 [SD 0.20]; LogMAR –0.13 [SD 0.06]; *P*<.01). Outcomes are reported in detail in Table 3. In this subgroup, a GEE multivariable analysis indicated a comparable confounding effect as described earlier regarding the overall outcomes.

**Table 3:** Refractive error and visual acuity measured in the mildly myopic participants (second-generation algorithm; N=86 eyes).

Refractive error and visual acuity	Manifest refraction^a^	Web-based refraction^a^	Difference	95% CI	*P* value^b^	GEE model^c^	
						Beta value	*P* value^c^
Power vector (Diopter)^d^	1.04 (0.86)	1.07 (0.96)	0.03	–0.13 to 0.06	.48	.82	<.01
J0 vector (Diopter)	0.05 (0.24)	–0.00 (0.23)	0.05	0.00 to 0.10	N/A^f^	N/A	N/A
J45 vector (Diopter)	–0.14 (0.14)	–0.10 (0.16)	–0.04	–0.04 to 0.03	N/A	N/A	N/A
Spherical equivalent (Diopter)	–0.98 (0.88)	–1.00 (1.00)	0.02	–0.09 to 0.13	N/A	N/A	N/A
Spherical power (Diopter)	–0.78 (0.85)	–0.87 (0.96)	0.09	–0.03 to 0.20	N/A	N/A	N/A
Cylindrical power (Diopter)	–0.38 (0.41)	–0.26 (0.50)	0.12	–0.24 to –0.02	N/A	N/A	N/A
Cylindrical axis (degrees)	101 (59)	105 (50)	4	–27 to 19	N/A	N/A	N/A
Corrected distance visual acuity logarithm of the minimum angle of resolution^e,g^	–0.14 (0.06)	–0.08 (0.10)	–0.06	–0.09 to –0.04	<.01	.15	.03
Corrected distance visual acuity Snellen^e,g,h^	1.39 (0.20)	1.22 (0.29)	0.17	0.11 to 0.23	N/A	N/A	N/A

^a^Unless otherwise specified, reported as mean (SD).

^b^Paired-sample *t* test performed for predefined primary and secondary outcome parameter only.

^c^Generalized Estimates Equation model to statistically correct for the inclusion of 2 eyes of one subject, age, and sex.

^d^Spherical and cylindrical power and axes were translated in vectors by Fourier analysis.

^e^Assessed with either the manifest or Web-based achieved correction.

^f^Not applicable.

^g^Snellen: decimal visual acuity.

^h^Statistical tests were performed only on predefined parameters (power vector for refraction and logarithm of the minimum angle of resolution for visual acuity).

## Discussion

### Principal Findings

In this noninferiority clinical trial, we compared a Web-based tool for measuring refractive error with the current gold standard, the subjective manifest refraction. Our analysis revealed excellent correlations between the 2 tests (ICC 0.92). Thus, we conclude that the Web-based test can be considered noninferior to manifest refraction. Importantly, the Web-based test provided a reliable measure visual acuity, similar to using a traditional wall chart, and visual acuity improved significantly using the prescription obtained by using the Web-based test, particularly among participants with mild myopia. This study provides the necessary validity and safety data for the Web-based eye test offered by Easee.

The Web-based tool measures the eye’s visual acuity and translates this outcome into refractive error, while assuming that any error is caused solely by an uncorrected refractive error. Thus, patients with a vision-limiting eye condition, such as amblyopia, cataract, or a retinal disease, may not necessarily obtain a reliable measure of refractive error by using the Web-based tool. In practice, this effect is mitigated by including a disclaimer for patients who have such an eye condition, although the patients must be aware of having such a condition to heed this disclaimer. It is also important to note that refractive errors in subjects with a high visual acuity or eye conditions that do not limit vision (eg, glaucoma or mild diabetic retinopathy) will likely not be detected by this Web-based test.

### Considerations

However, some limitations of the study itself should be taken into consideration. No randomization of the test order was performed and could have impacted our results. Subjects may become tired during the assessments. Although because of the fixed test order, this should have impacted all subject similarly. We consider the learning or training effect during the tests as negligible. The 3 methods of refractive assessment are very different, and randomized projected optotypes were used to assess visual acuity. In addition, subjects were blinded for the outcome to prevent testing bias. Notwithstanding, the observer had access to the test outcomes; thus, an observer bias cannot fully be excluded. The Web-based test has been validated for use in healthy individuals. Further studies should be performed to test the feasibility of using this Web-based tool in children and populations with a higher incidence of eye disease. Another consideration is the role of accommodation during the test, which is defined as a semivoluntary reflex, causing the eye to focus on a nearby object; this reflex can increase the eye’s refractive power and can therefore mask a residual hyperopic refractive error. We found that the Web-based test tended to underestimate a hyperopic refractive error by an average of 0.5 D, which suggests that the accommodation reflex may have played a role in these participants. We consider the manifest refraction test a more powerful tool to measure the full hyperopic refractive error. Nevertheless, undercorrecting a hyperopic refractive error may be preferred over issuing the full-strength prescription, and this can sufficiently alleviate the patient’s visual complaints [24]. All measurements were performed in accordance with ISO standards regarding visual acuity testing, revealing that a fully autonomous algorithm is capable of nearly matching the results obtained by an optometrist, at least in a healthy population. In daily practice, not all refraction assessments are performed by an optometrist, and not all assessments are performed under ideal conditions. Depending on local regulations and customs, a technician or a trained optician may perform the exam. Moreover, an authoritative consumer report revealed that prescriptions issued by eye care professionals can have wide variability [25]. Importantly, although our Web-based refraction test depends on the patient’s input, it has zero variability with respect to interpreting the patient’s responses, and it should provide high test-retest reproducibility. Further research is needed to determine whether the Web-based tool has high intrasubject consistency.

### Practical Perspective

The recent increase in digitization has increased the availability and accessibility of the Web-based refraction test, as anyone with a laptop and smartphone can complete the test without the need to visit an eye care professional. Moreover, 2.7 billion people are estimated to have a smartphone in 2019 [26], and approximately 97% of our target patient population—users from 18 to 45 years of age—have a smartphone [27]. The availability of a Web-based refraction fits into the current trend of digitalization, and this provides consumers with more flexibility in planning their eye test. In addition, the Web-based refraction benefits patients in areas with limited access to eye care professionals. Basatwrous et al have convincingly shown that creating a comprehensive digital eye care ecosystem can elevate the overall health in a rural community [28]. The Research in Context panel summarizes current initiatives on remote eye testing. A future perspective is the use of the Easee eye test in a clinical environment with automated data entry in the electronic health record, as well as integration in clinical care, for example, cataract, macular degeneration, and glaucoma patients. The measurements provided by the Web-based refraction test were not subjected to post hoc processing, and these were entered directly into the database for analysis. Despite the high rate of concordance between the Web-based refraction test and the manifest refraction test, the Web-based test was not able to detect all outliers and unusual results. Therefore, additional interpretation of previous prescriptions and remote validation of the data by a qualified optometrist may still be warranted. Importantly, the Web-based refraction test is not designed to fully replace a comprehensive eye exam by a trained eye care professional, and users must comply with the test’s terms and conditions; failure to do so will prompt the advice to visit an eye care professional.

### Conclusions

Here, we report that the Easee Web-based test for measuring refractive error provides a safe, valid method for obtaining a corrective prescription in individuals with healthy eyes, particularly patients with mild myopia. Using the prescription obtained with the Web-based test significantly improves visual acuity to a degree similar to the prescription obtained using manifest refraction. Therefore, the Web-based refraction test provides a user-friendly, easily accessible alternative to the traditional subjective manifest refraction test, although it should not be considered a replacement for a comprehensive eye examination. The Web-based test is CE marked; therefore, it meets the requirements established by the European Union with respect to safety and health.

## Appendix

Multimedia Appendix 1 Panel - Research in context.

Multimedia Appendix 2 Clinical test flow second generation online refraction test.

Multimedia Appendix 3 Clinical test flow second generation online refraction test.

Multimedia Appendix 4 Supplementary data overall results.

## Abbreviations

CDVA: corrected distance visual acuity
CE: Conformité Européenne
D: diopter
GEE: generalized estimating equations
ICC: intraclass correlation coefficient
ISO: International Organization for Standardization
LogMAR: logarithm of the minimum angle of resolution
MORE: Manifest versus Online Refraction Evaluation
SEQ: spherical equivalent
UDVA: uncorrected distance visual acuity
