# Supplementary material for: Validation of an Independent Web-Based Tool for Measuring Visual Acuity and Refractive Error (the Manifest versus Online Refractive Evaluation Trial): Prospective Open-Label Noninferiority Clinical Trial
Source: J Med Internet Res. 2019 Nov 8;21(11):e14808. doi: 10.2196/14808 (PMC6874802; doi:10.2196/14808)
Supplement: Multimedia Appendix 1 [file jmir_v21i11e14808_app1.pdf]

Research in context

Evidence before this study

We performed an unrestricted search of PubMed and EMBASE for relevant studies published through February 2018 regarding remote and/or online eye testing; additional studies were identified by searching relevant websites and the references listed the identified publications. Twenty-six relevant studies and/or equivalent medical devices were identified, reporting six different devices and services.

The refractive state of the eye can be assessed using the SVONE device (https://www.smartvisionlabs.com/), a handheld smartphone-based autorefractor validated for use in adults and children. Similarly, EYENETRA (https://eyenetra.com/) offers a suite of validated smartphone-based optometry devices. Both sets of devices have excellent concordance with the manifest refraction reference test; however, these devices are aimed at opticians and optometrists, rather than the consumer.

Currently, visual acuity can be measured online using Peek (https://www.peekvision.org/), Paxos (https://www.digisight.net/), and GoCheck (https://www.gocheckkids.com/). Peek has been validated for clinical practice and community-based fieldwork in 300 subjects, and Paxos (DigiSight) was found to be reproducible for home measurements by diabetic patients. Finally, the amblyopia screening tool GoCheck has been validated for use in a pediatric population.

An online tool for measuring both refractive error and visual acuity is currently offered by Visibly (formerly Opternative; https://www.govisibly.com/); this medical device is the most similar to the online tool described in our study. Clinical results have not been reported in peer-reviewed publications; however, the outcome of a trial are available online, showing a satisfactory rating for the refractive exam (8.6 out of 10) and relatively high confidence among users (8.7 out of 10). Moreover, 88% of prescriptions obtained using Visibly were within 0.5 D of the prescriptions obtained using manifest refraction, with excellent correlation (r = 0.93).

Added value of this study

This prospective open-label clinical trial involving 200 healthy eyes is the first to report the validity and safety of an online self-assessment tool for measuring refractive error. The tools algorithm has excellent correlation with the reference test and is non-inferior to a manifest subjective refraction reference test conducted by a trained optometrist. Visual acuity is significantly improved using the prescription obtained with the online tool, and this improvement is similar to the improvement obtained with the prescription based on the reference test, especially particularly in participants with mild myopia.

Implications of all the available evidence

The digitization of healthcare is rapidly changing the way in which eye testing can be performed, and smartphone-based eye tests have been shown to be both safe and reliable when combined with dedicated devices in the optician’s practice. Independent online self-assessment tools for measuring visual acuity is both robust and accessible, and is currently used worldwide in a variety of telemedicine and screening programs. Our results indicate that our novel online self-assessment tool is also valid for measuring refractive error in healthy eyes.
