# Supplementary material for: Validation of an Independent Web-Based Tool for Measuring Visual Acuity and Refractive Error (the Manifest versus Online Refractive Evaluation Trial): Prospective Open-Label Noninferiority Clinical Trial
Source: J Med Internet Res. 2019 Nov 8;21(11):e14808. doi: 10.2196/14808 (PMC6874802; doi:10.2196/14808)
Supplement: Multimedia Appendix 2 [file jmir_v21i11e14808_app2.pdf]

Supplementary data to MORE-trial (JMIR ms#14808)

Complete flow of clinical study environment, including requested responses by the user.

Computer	Phone

Accessing the Clinical Study Exam

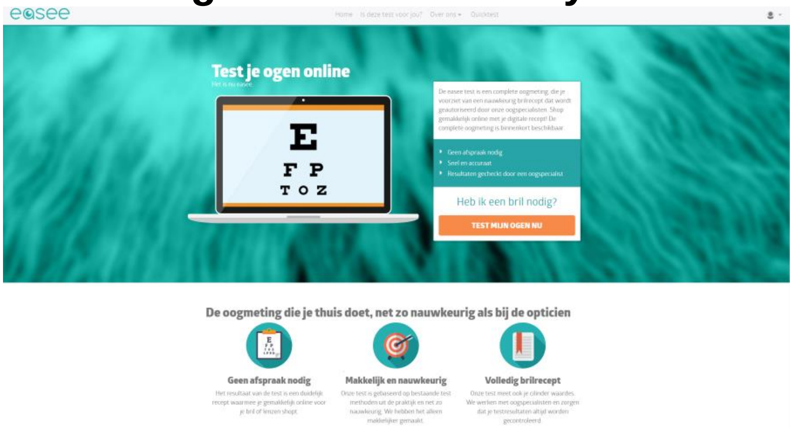

The front page of the web site which host the exam.

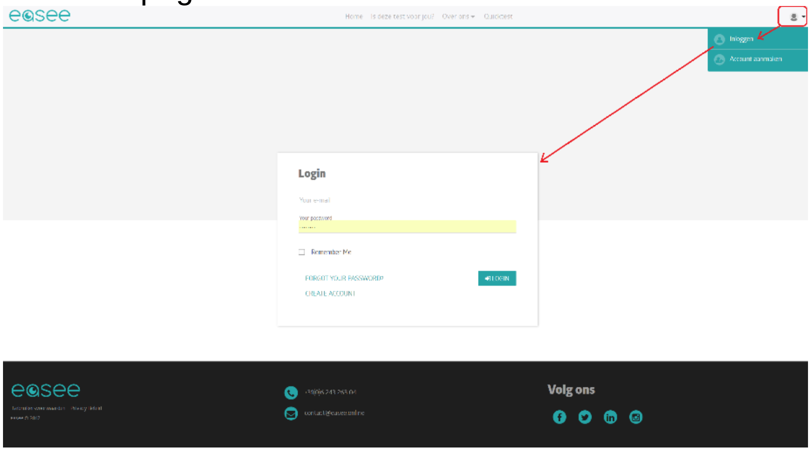

The observer logs into the system with a dedicated account.

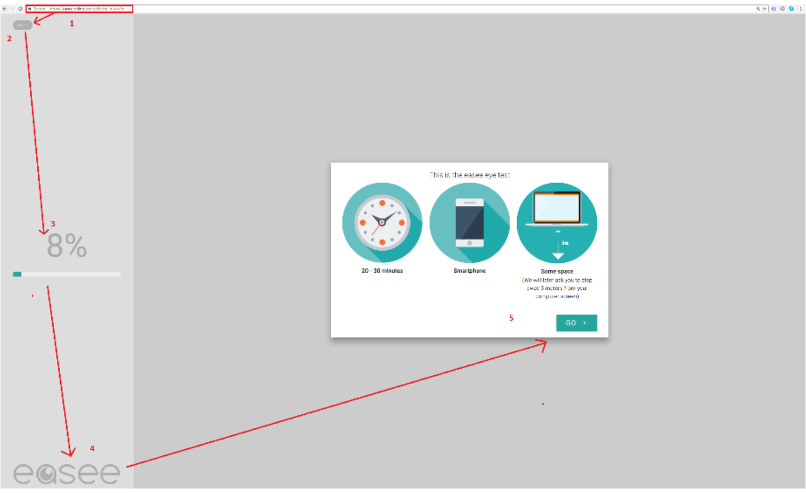

1. The url to the exam
2. The verion number of the exam
3. The progress status of the exam

4. The branding of the exam
5. The exam wizard navigation

This is the first screen of the exam the users see. It contains an overview to familiarize the user with what it takes to complete the exam

Setting up the environment

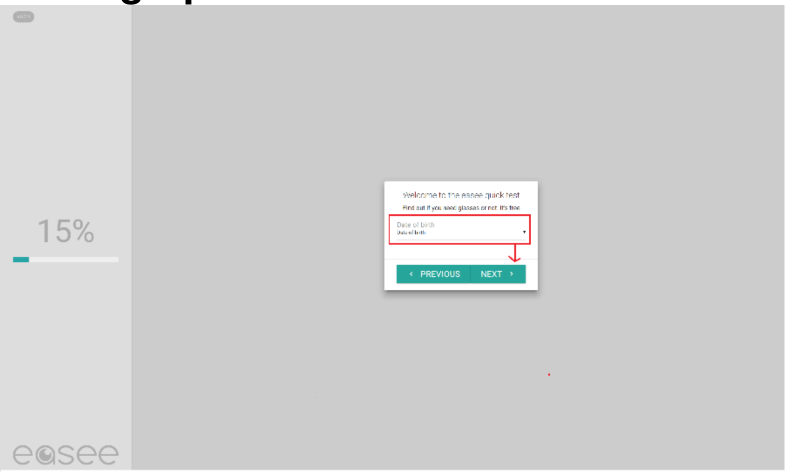

The user will be asked to provide his year of birth.

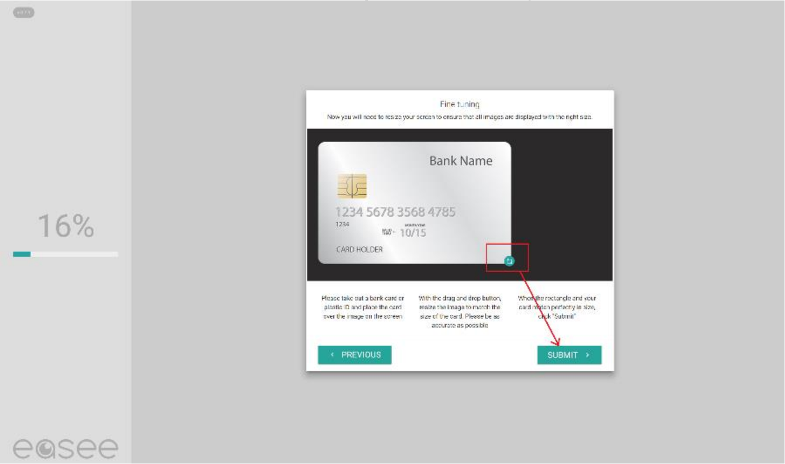

The user will be asked to use a credit card to calibrate his screen. This step is necessary for tool to present exam images later in the right size.

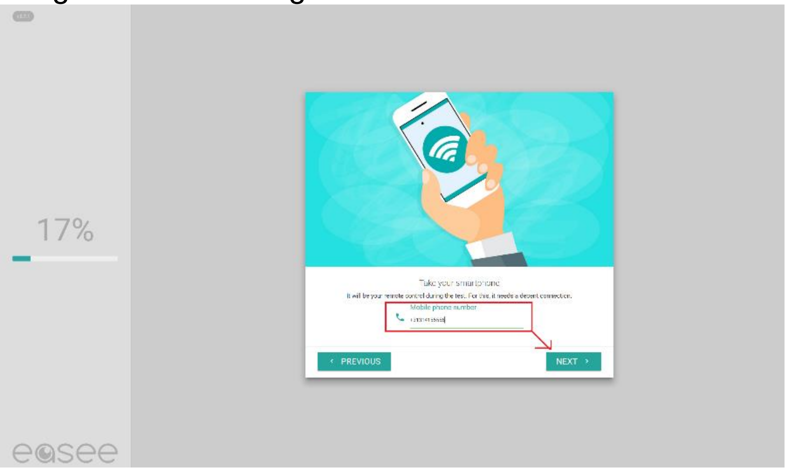

The user will then be asked to provide a valid phone number. A link will will be sent using sms, which, when clicked will transform his phone into a remote control for the exam.

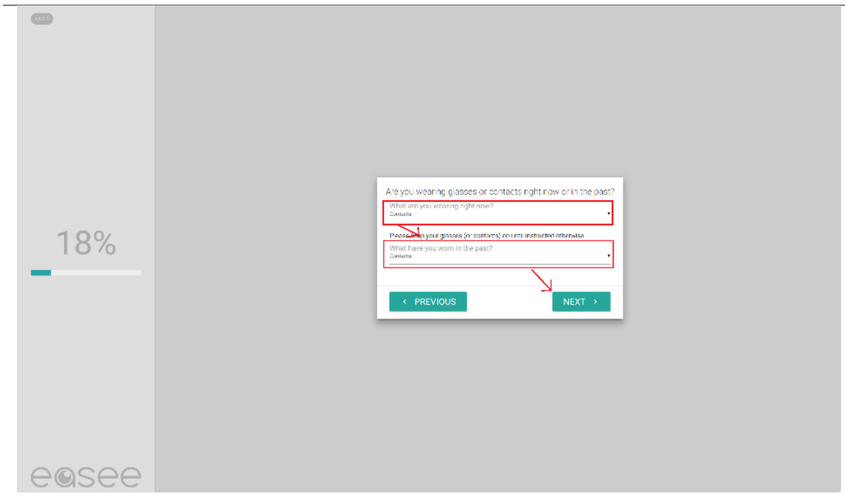

The user will be asked whether he current uses glasses/contacts or in the past. This information will be used to determine whether certain section of the exam are necessary to take aided.

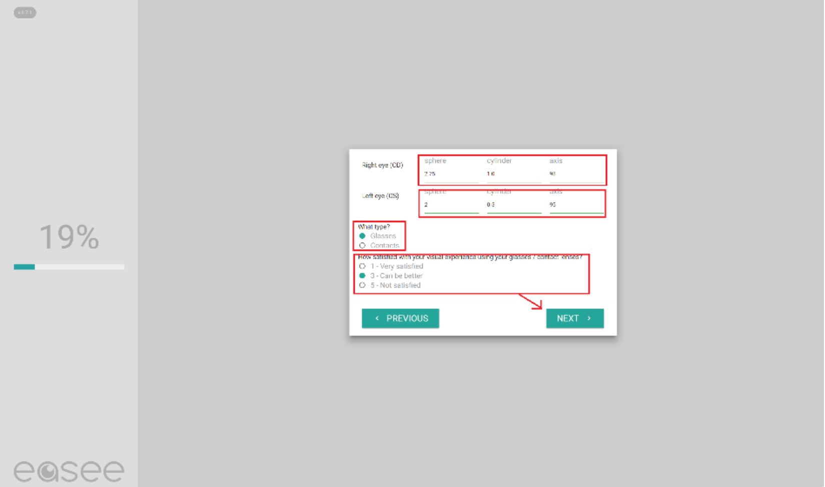

If the user has stated he has used contact/glasses at point in time, he will then be be asked to provide his prescritption is he has it available.

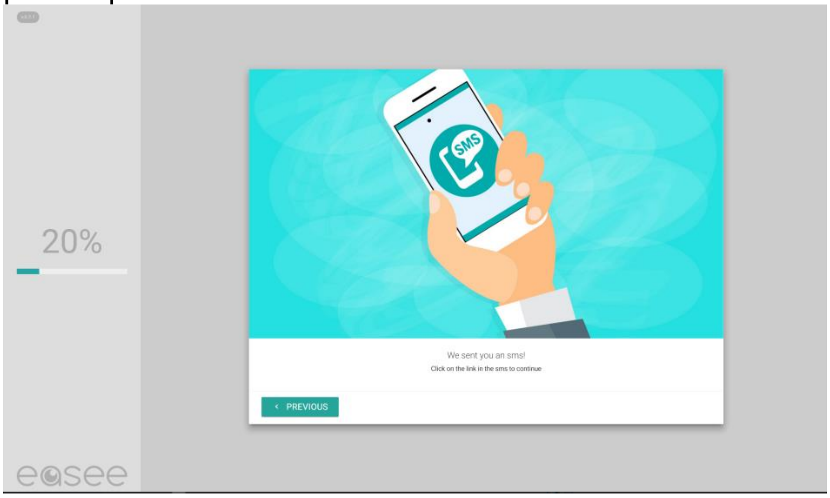

The user will then be asked to check whether he has received an sms on phone and if so, click on the url link that is attached.

Establishing connection with phone

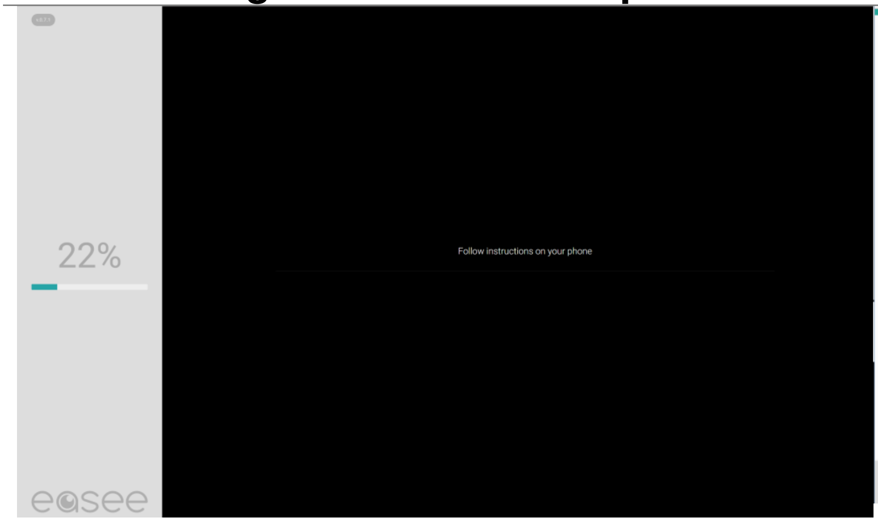

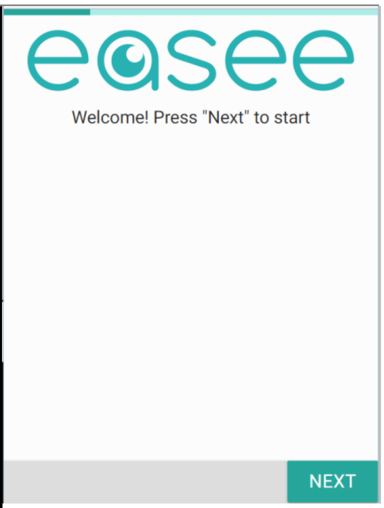

A confirmation screen will be presented once the user has successfully clikcked on the sms on his phone.

Taking the aided visual acuity exam

The user will be expected to be standing 3 meters away from his computer.
All other environment setups will have been conducted by the doctor supervising the exam.
e.g. screen brightness, dimly lit room, screen saver off.

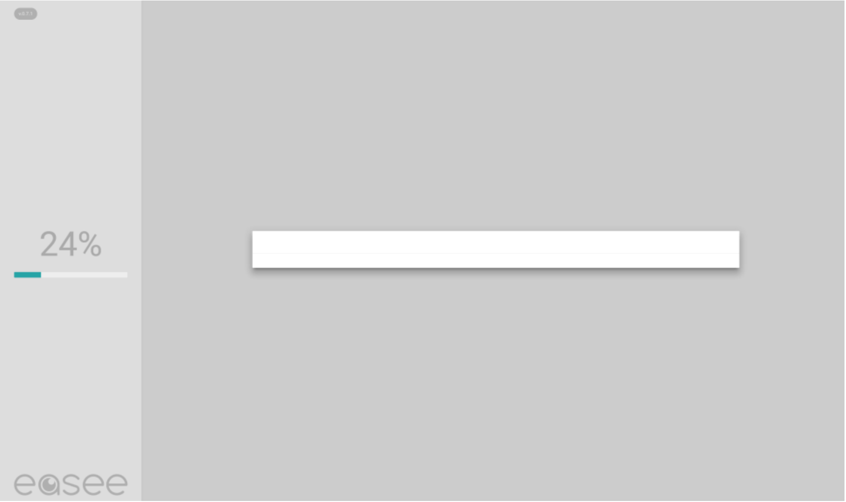

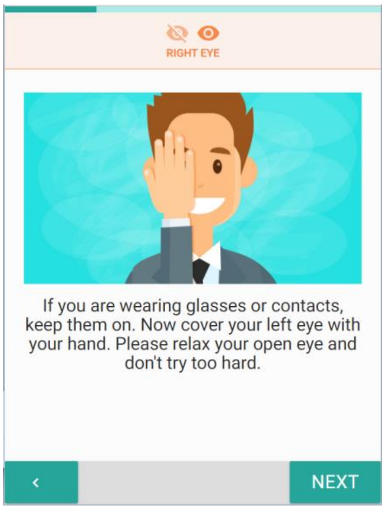

He should cover his left eye as instructed. And should be wearing his glasses/contact on.

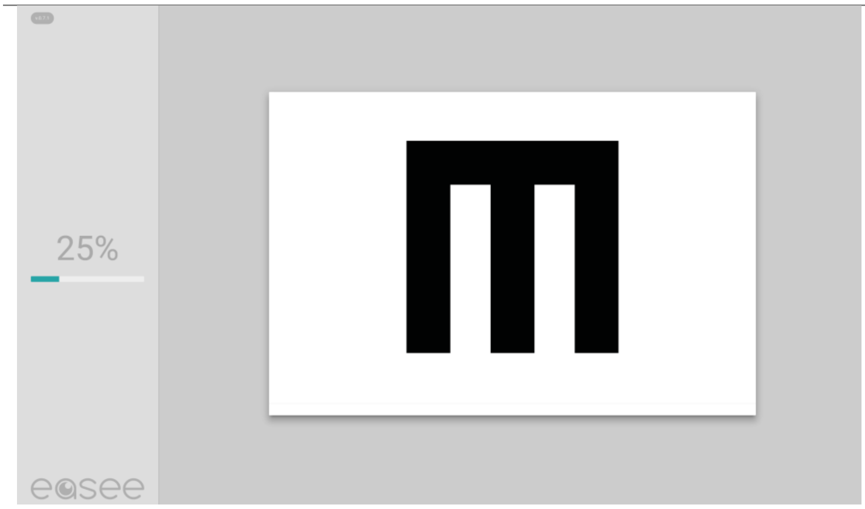

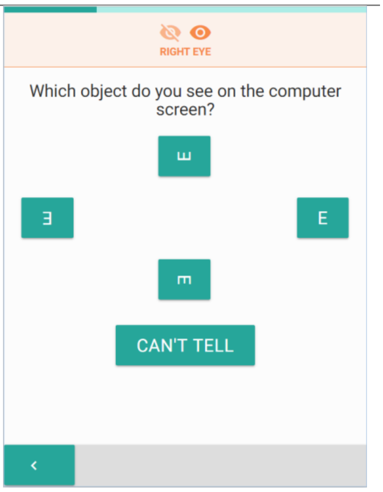

The user will be presented a sequence of tumbling E images on the computer screen.
He will user his phone to provide answers that correspond to the image he sees on the computer screen.

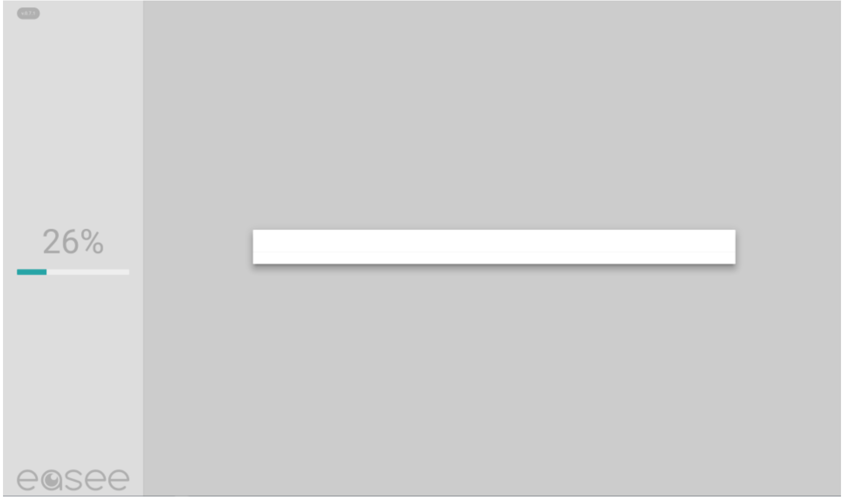

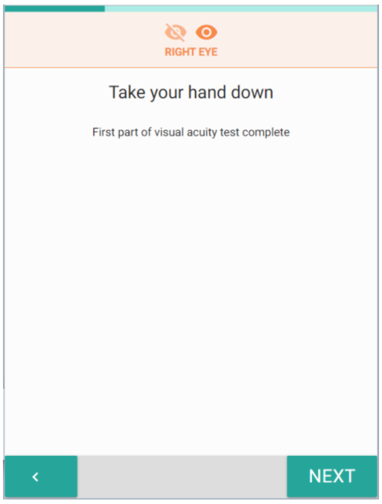

The user will be shown a screen to confirm completion of the exam for the right eye.

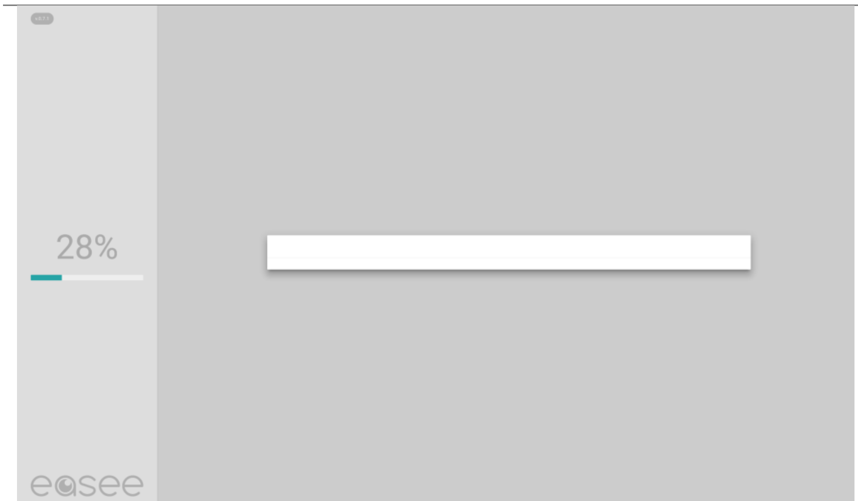

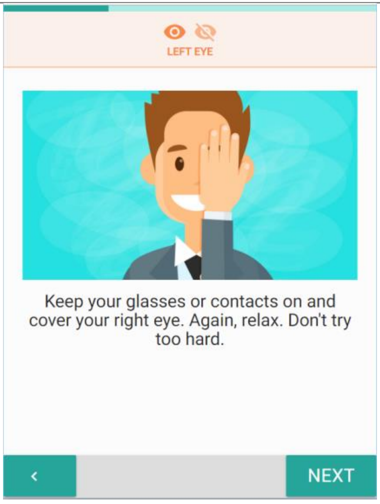

The exam will then repeat the sequence for the left eye.

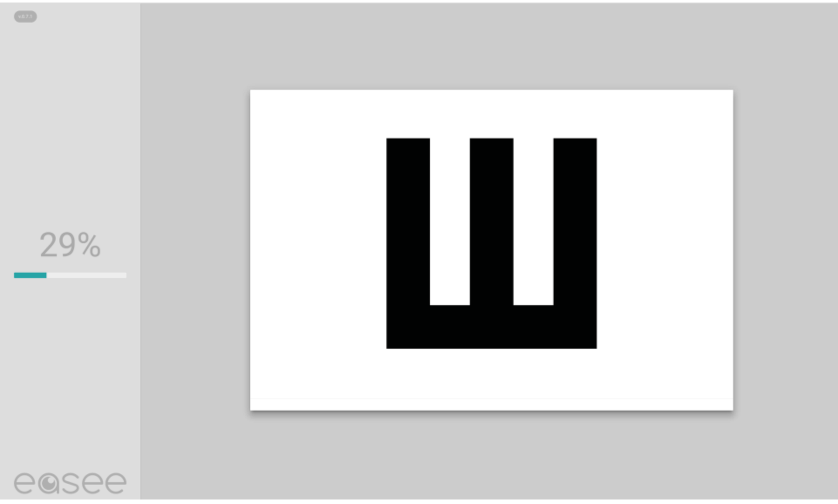

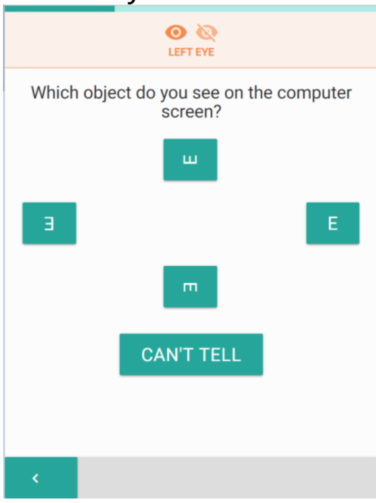

The user will be presented a sequence of tumbling E images on the computer screen.
He will user his phone to provide answers that correspond to the image he sees on the computer screen

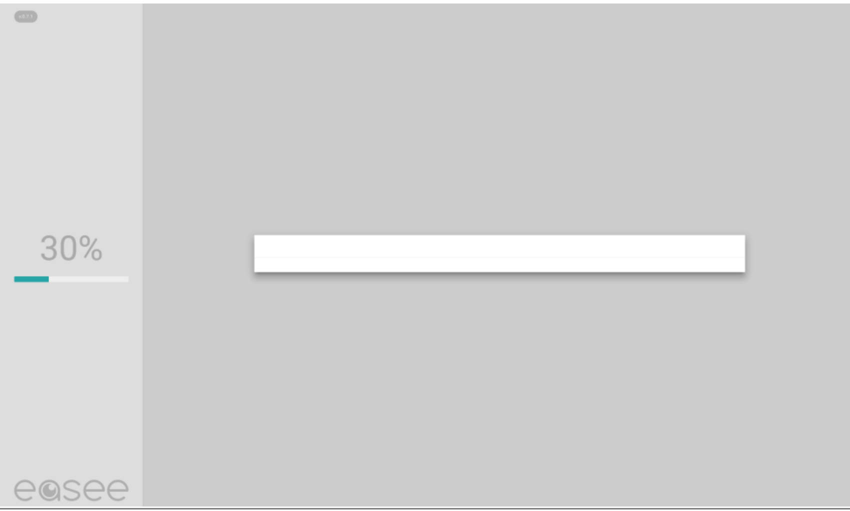

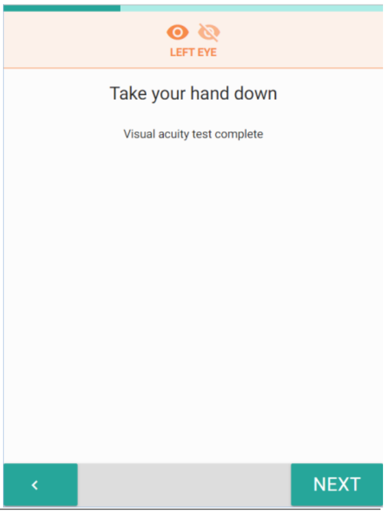

The user will be shown a screen to confirm completion of the exam for the left eye.

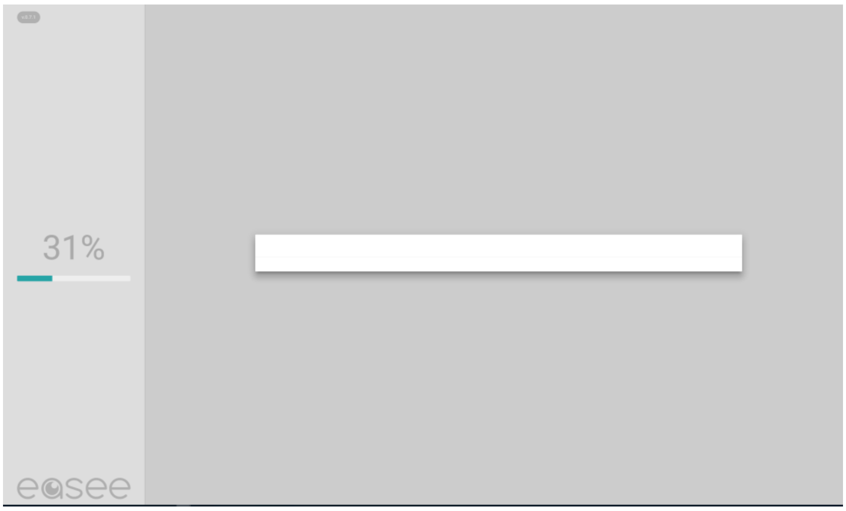

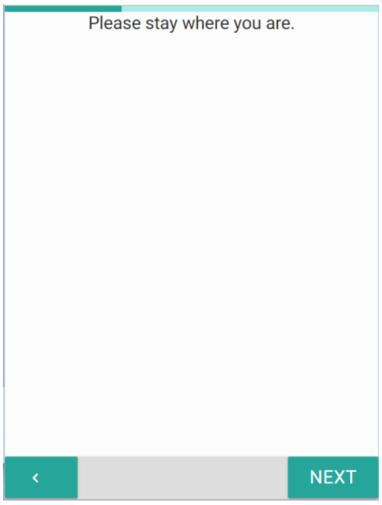

For the next part of the test the user is asked to remain at the same distance of 3 meters to the screen.

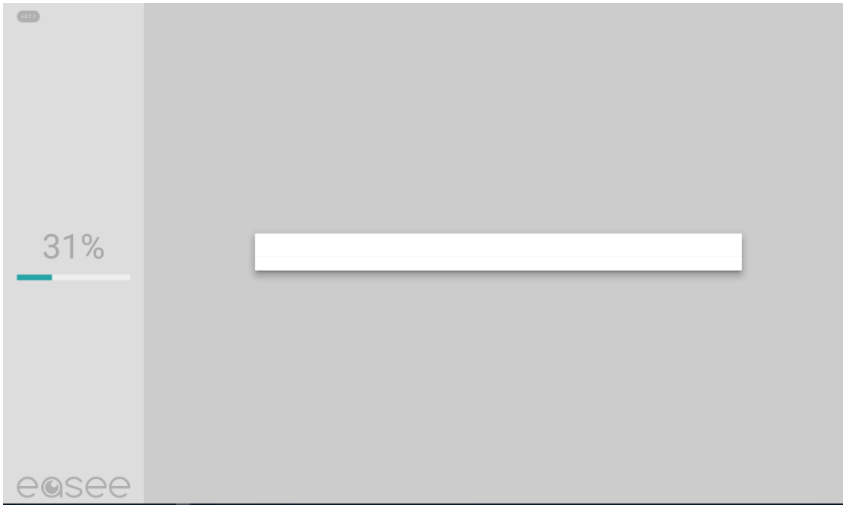

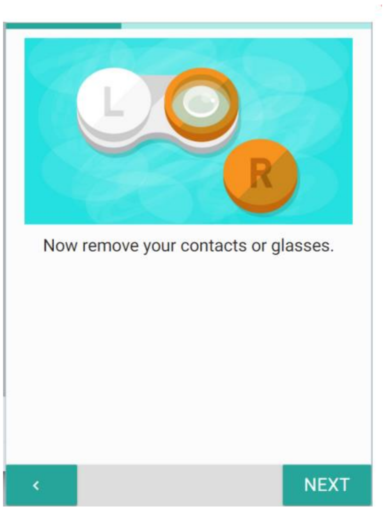

Now the user is instructed to remove the glasses or contacts.

Taking the aided visual acuity exam

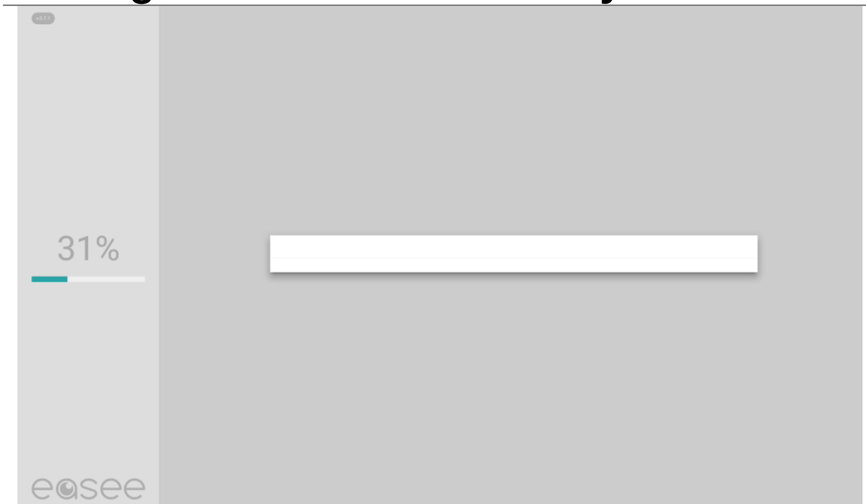

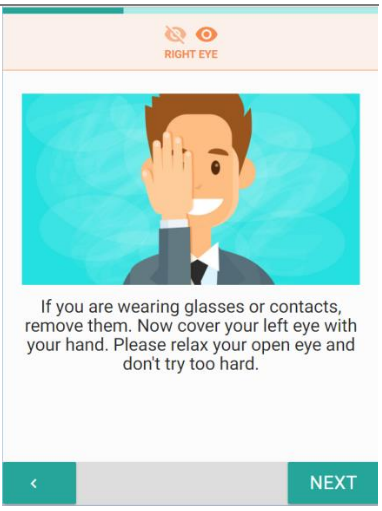

Similar to the aided visual avuity test the unaided refraction assessment starts with presenting tumbling Es.

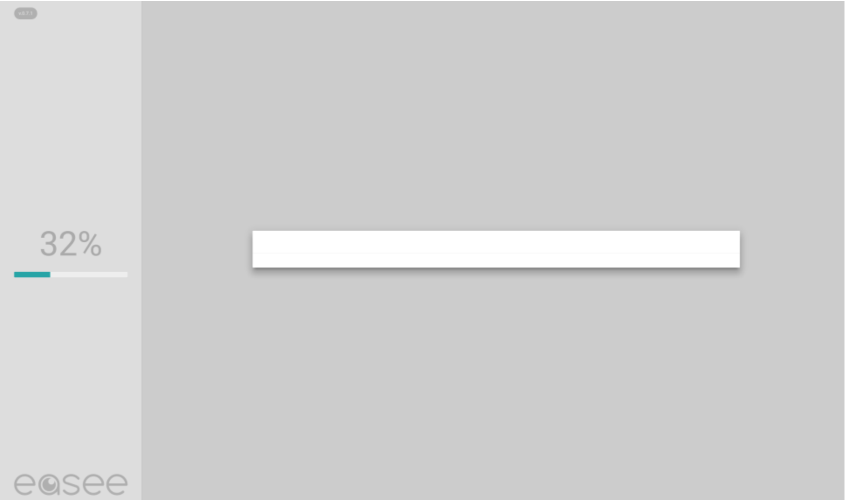

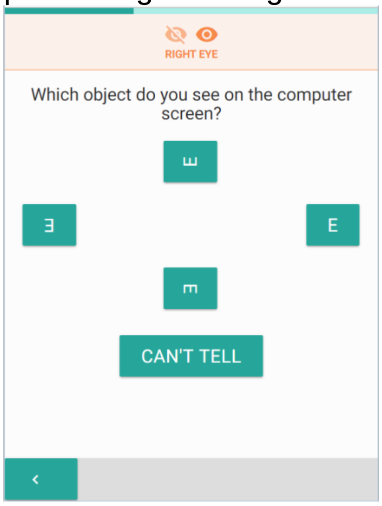

Similar to the aided visual acuity test, the user will again be presented a sequence of tumbling E images on the computer screen. He will user his phone to provide answers that correspond to the image he sees on the computer screen

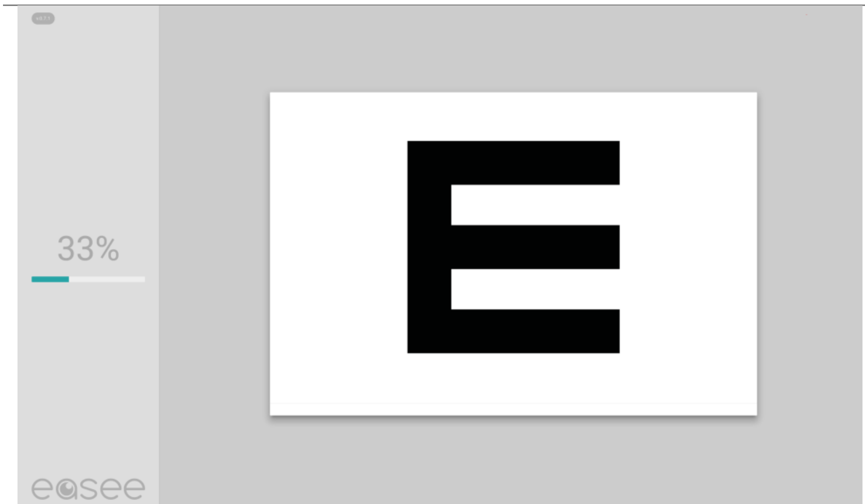

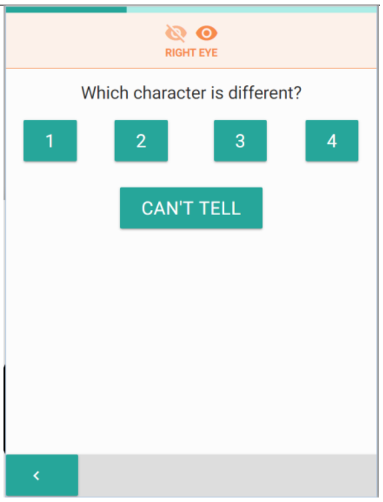

Taking the

The next part of the test the exam fomat changes. Now the user will be presented 4 images of which one will be different. The user will have to indicate on his phone which image looks different, or click ’can’t tell’ if they all look the same.

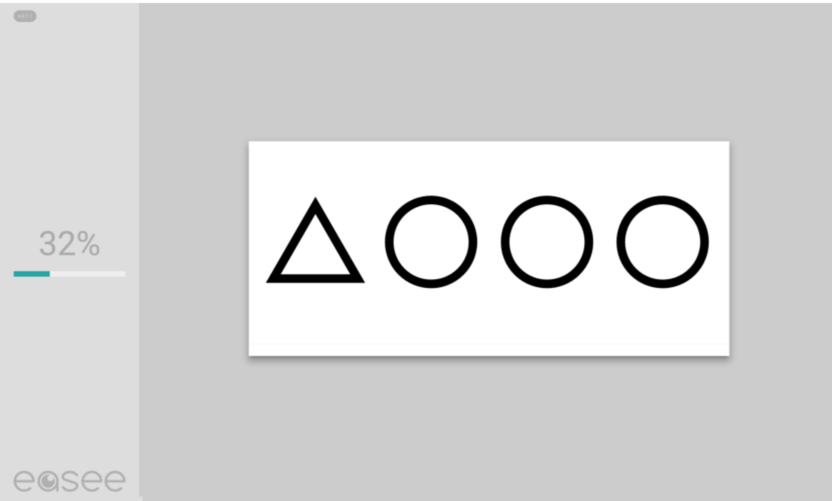

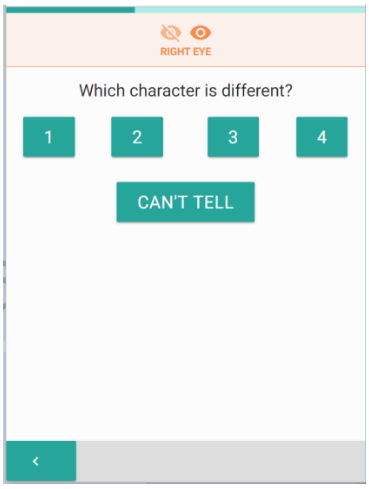

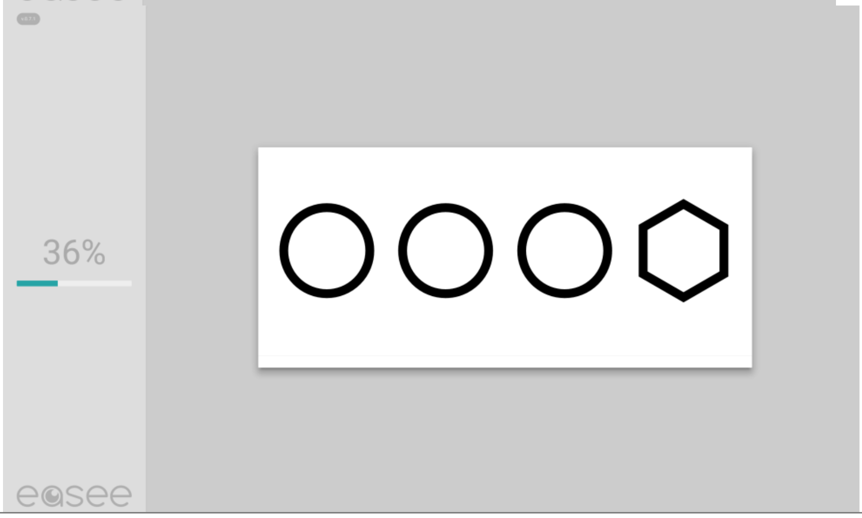

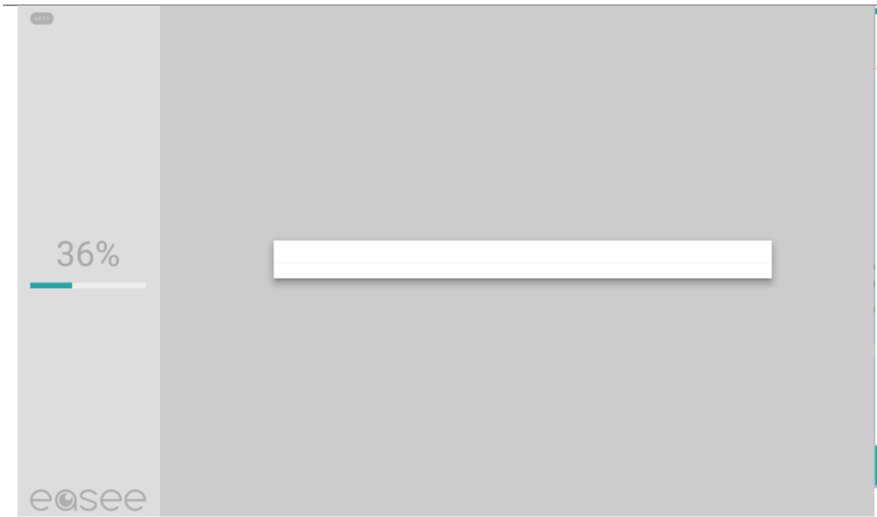

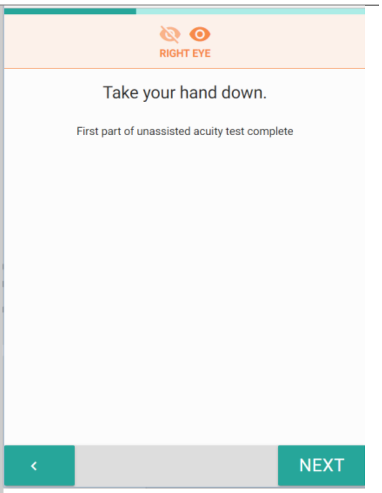

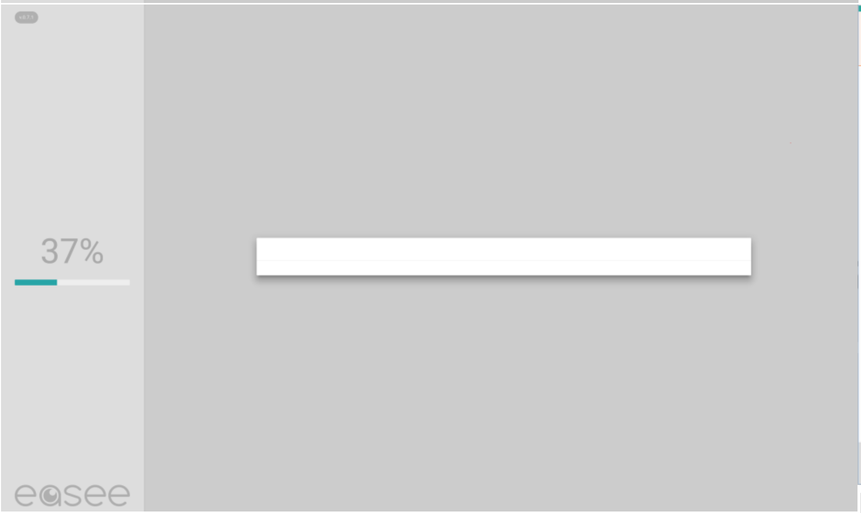

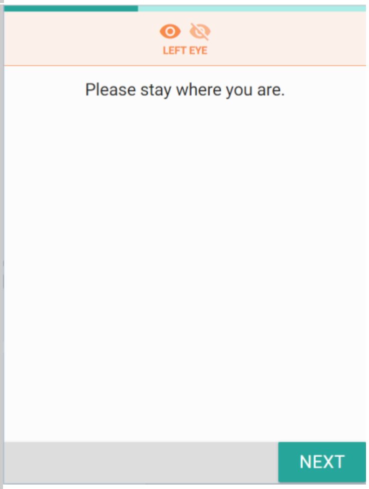

For the next part of the test the user is asked to remain at the same distance of 3 meters to the screen.

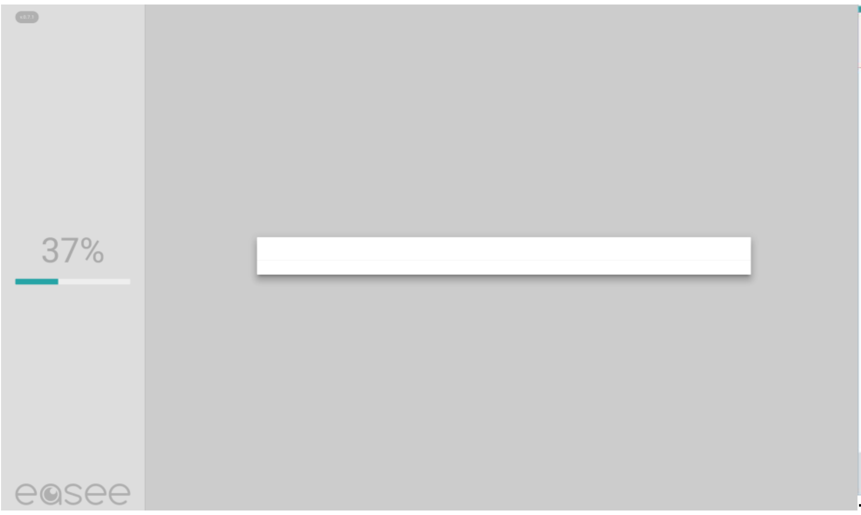

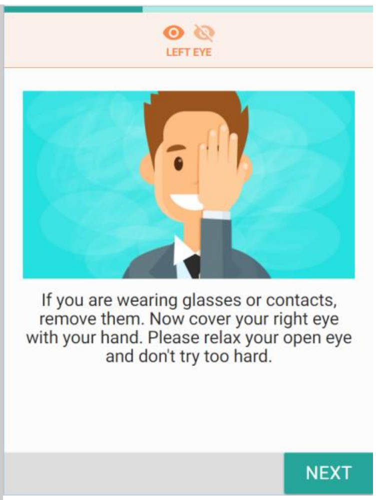

The test is now reapeated for the left eye.

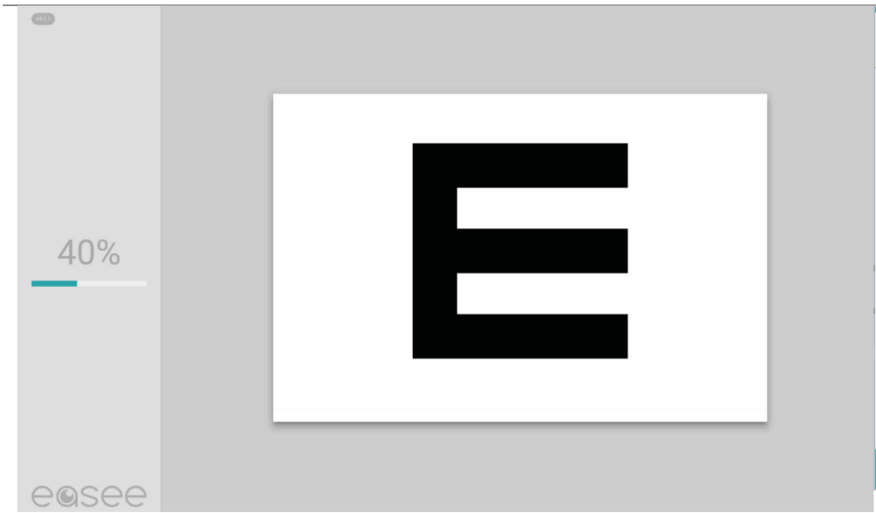
40%
easee
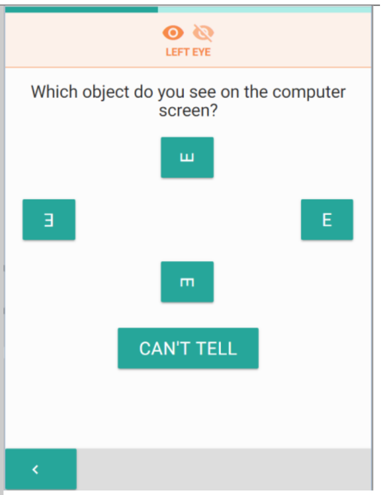
LEFT EYE
Which object do you see on the computer screen?
E
CAN'T TELL
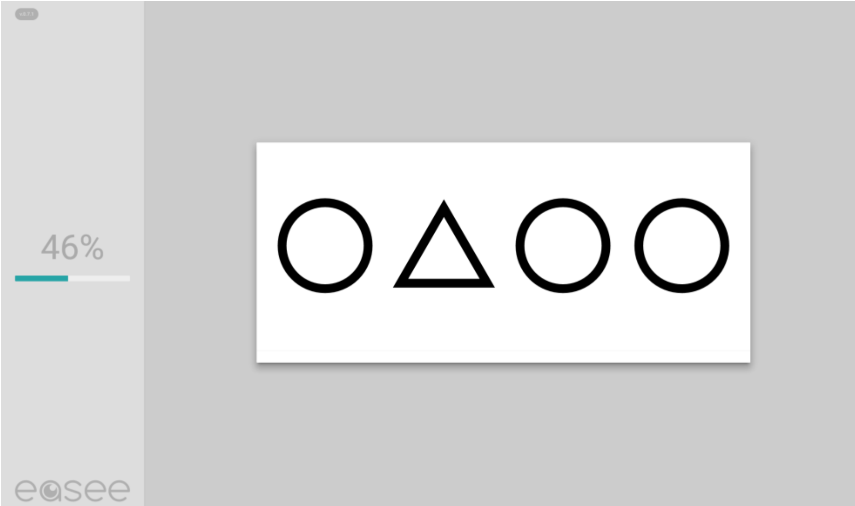
46%
easee
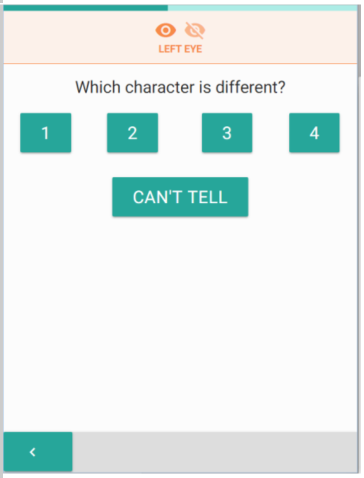
LEFT EYE
Which character is different?
1
2
3
4
CAN'T TELL
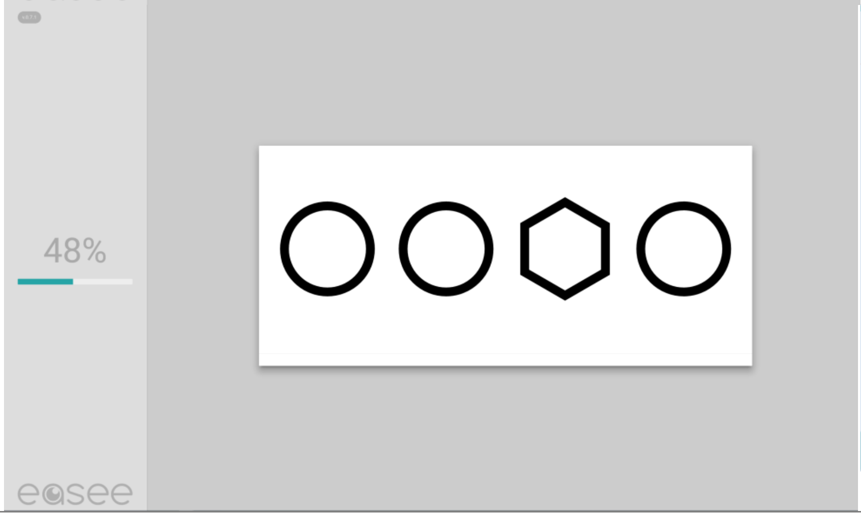
48%
easee
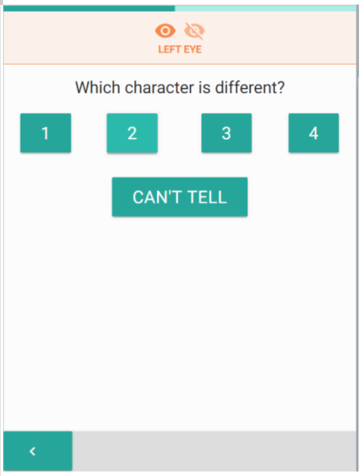
LEFT EYE
Which character is different?
1
2
3
4
CAN'T TELL

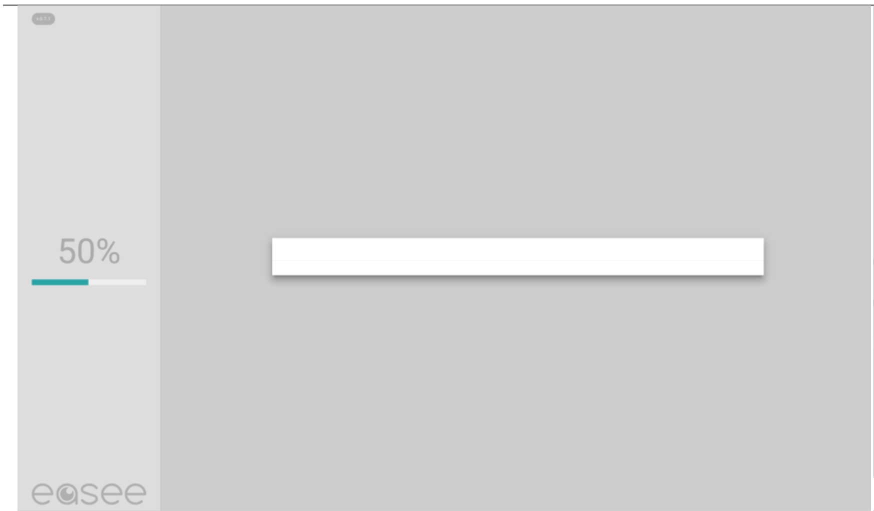

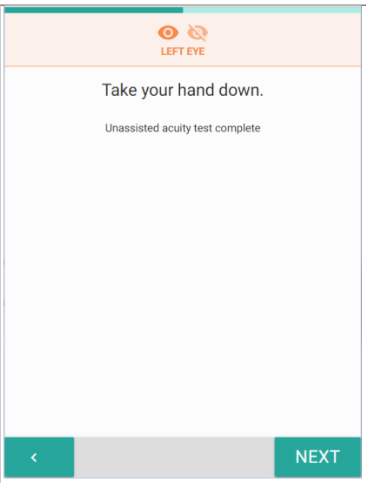

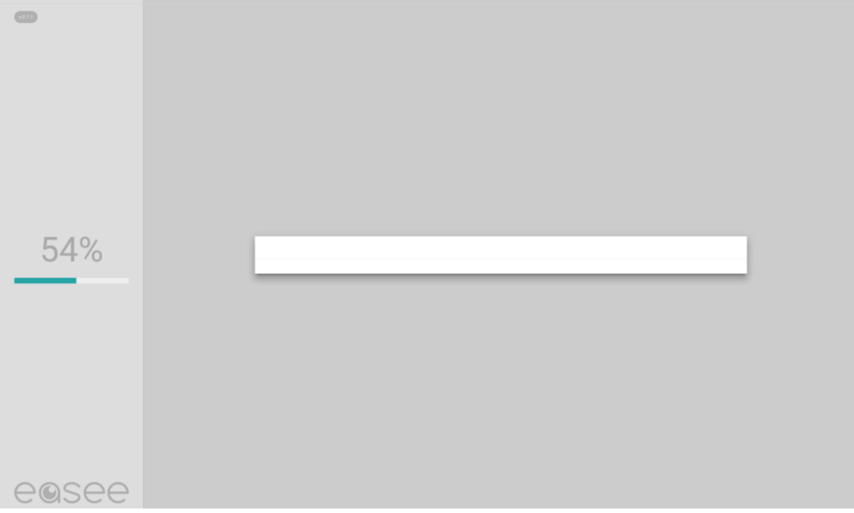

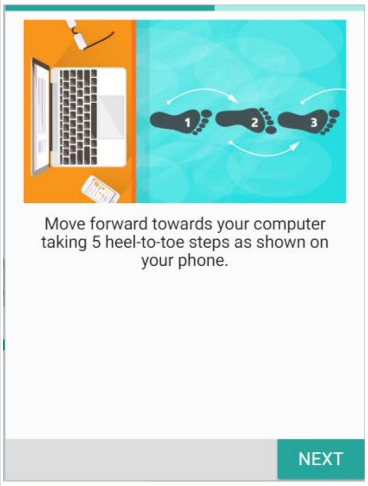

After completing the unaided visual acuity part of the exam the user is intructed to move 5 heel-to-toe steps towards the screen.

Taking the duochcrome exam

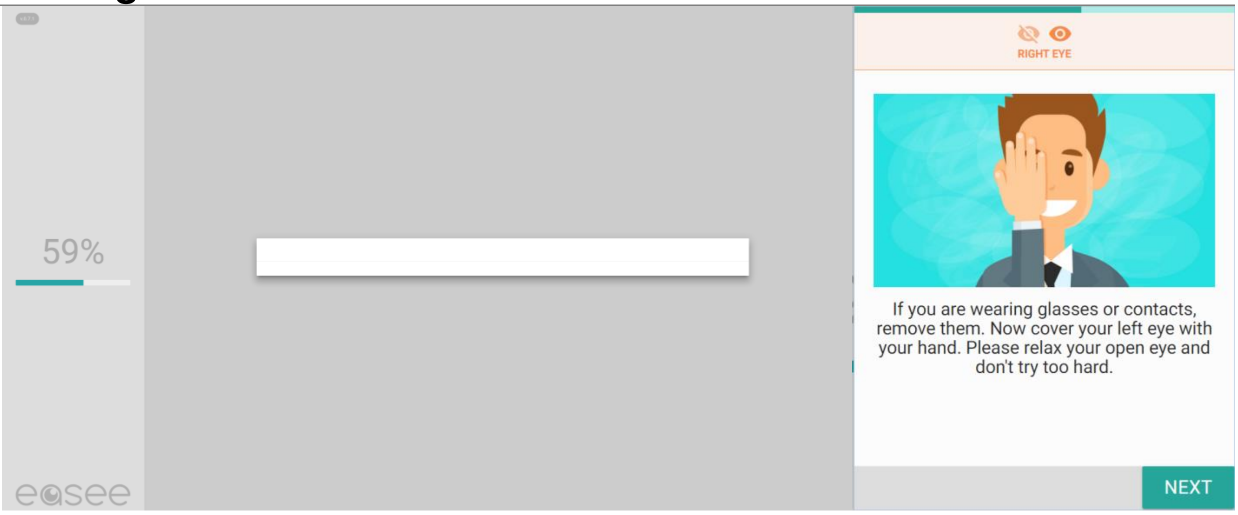

The user is again asked to cover his left eye to execute the next part of the test.

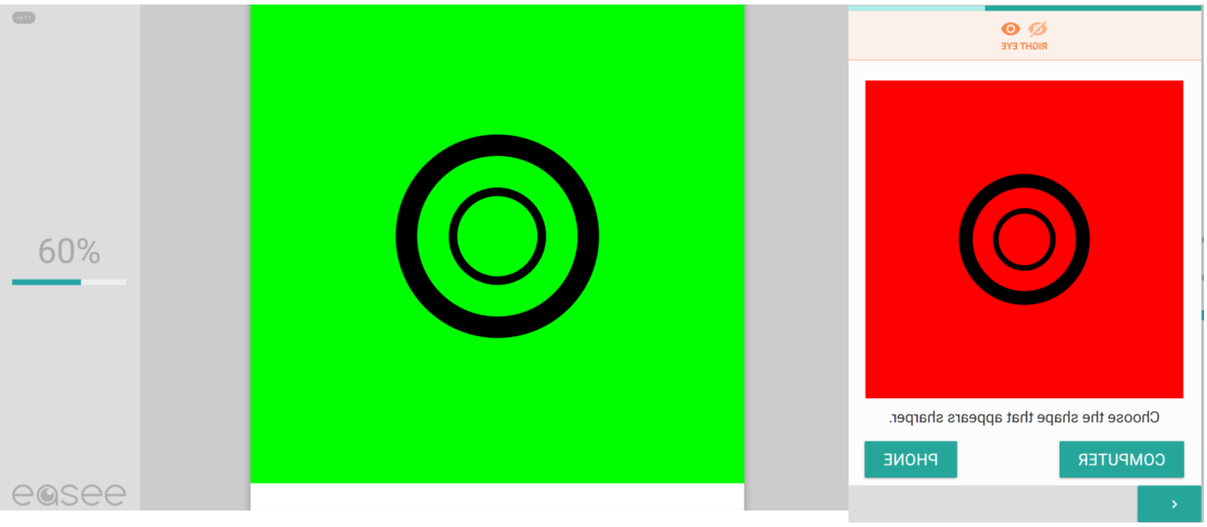

The user is presented a green image on the computer screen and simultanioulsy a red image on the phone screen. The user is asked to first look at the image on the phone and then on the computerscreen and indicate which of the two looks clearer.

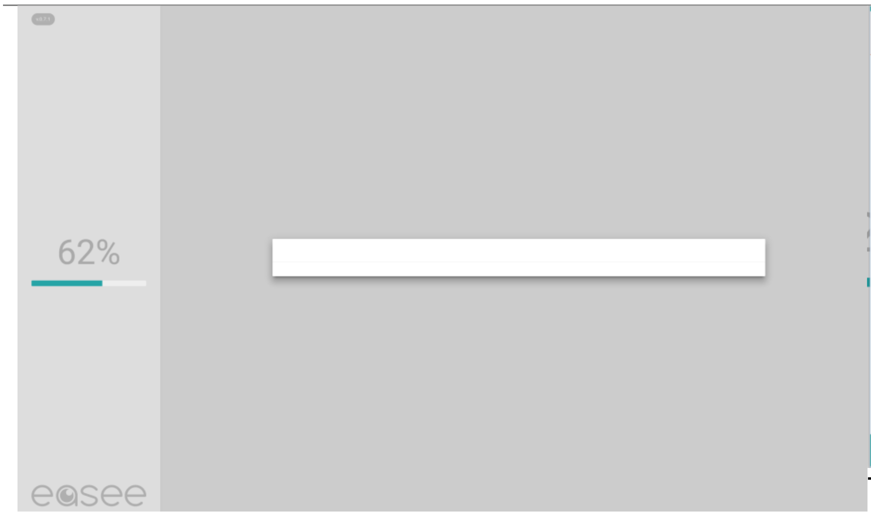

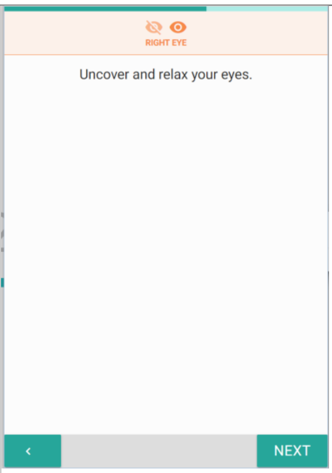

The user will be shown a screen to confirm completion of this part of the exam for the right eye.

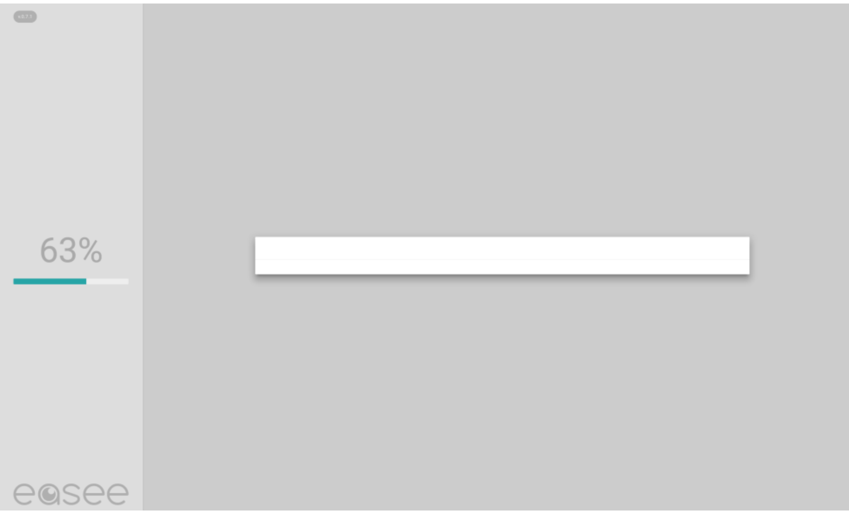

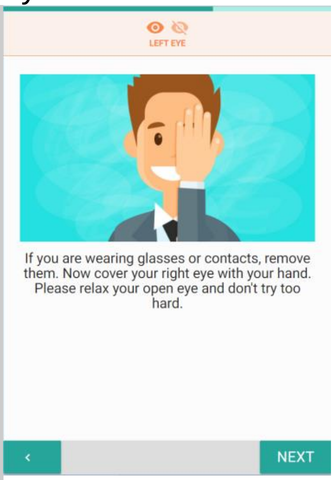

The user is now asked to cover his right eye to execute the next part of the test.

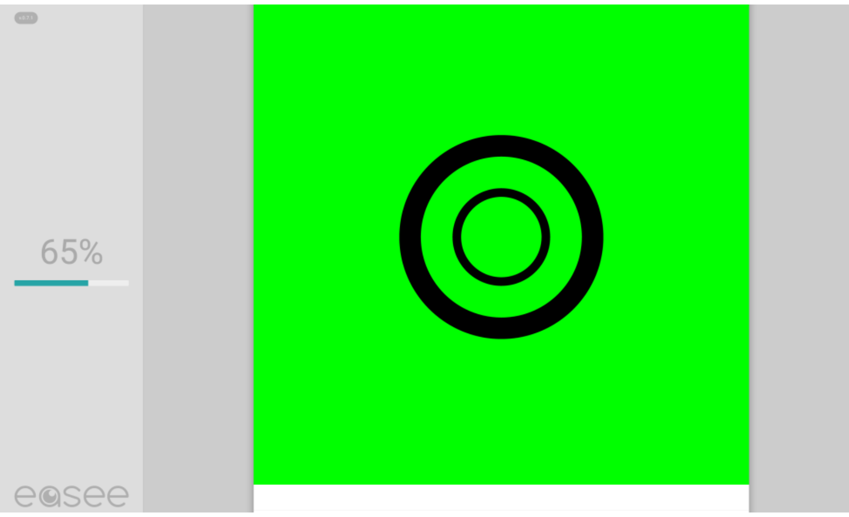

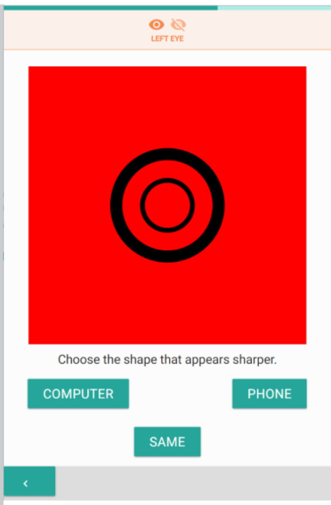

The user is again presented a green image on the computer screen and simultanioulsy a red image on the phone

screen. The user is asked to first look at the image on the phone and then on the computerscreen and indicate which of the two looks clearer.

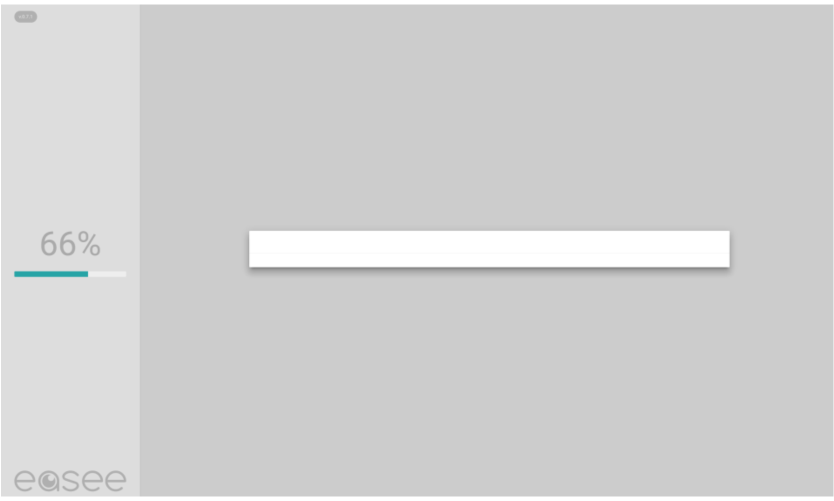

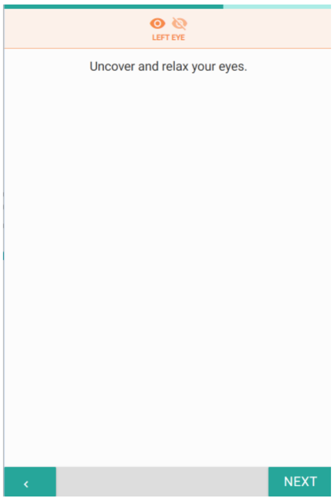

The user will be shown a screen to confirm completion of the exam for the left eye.

Taking the visual experience questionaire for myopia / hyperopia determination

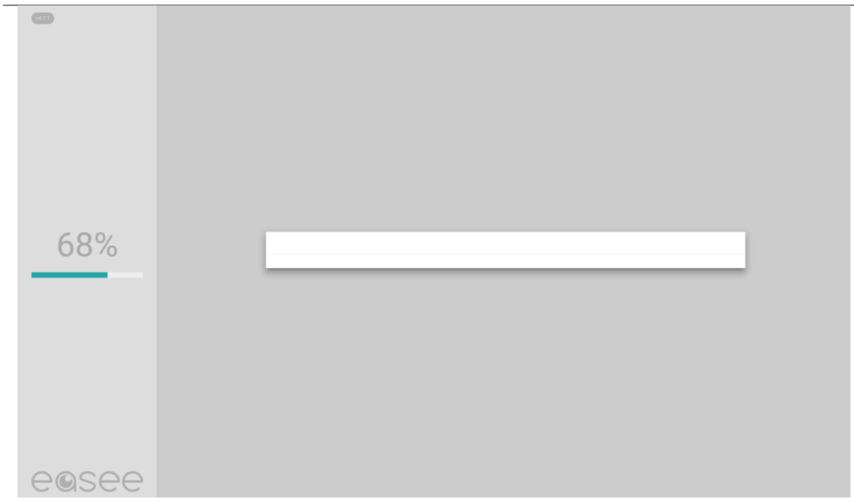

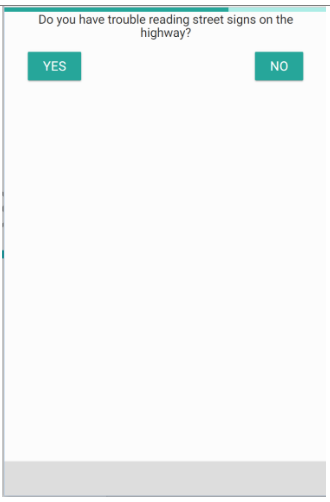

Now, the user is presented a set of Yes/ No questions to get more information about the refractive condition.

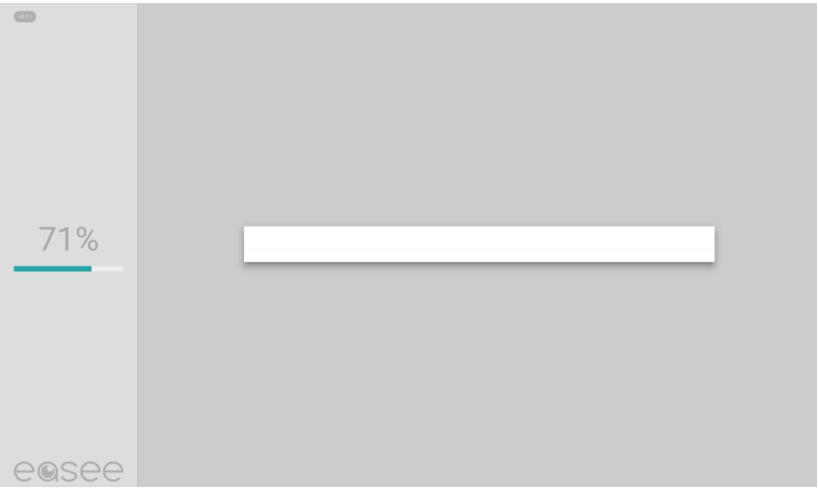

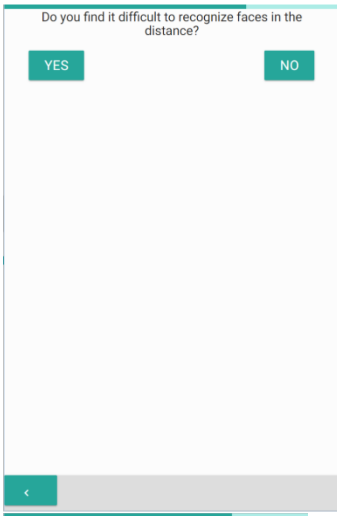

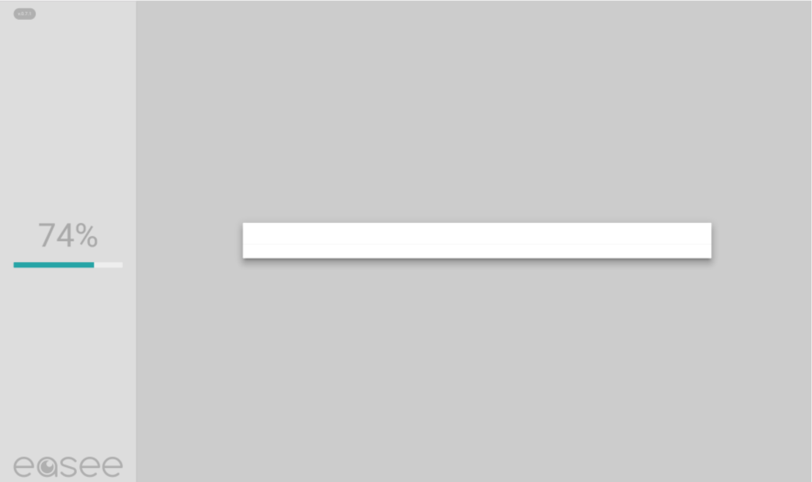

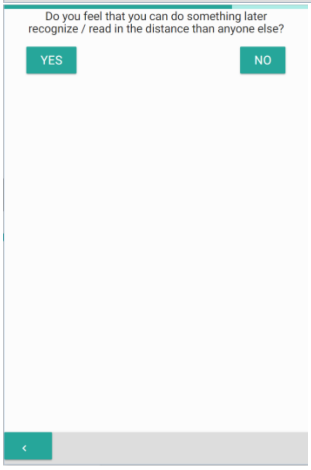

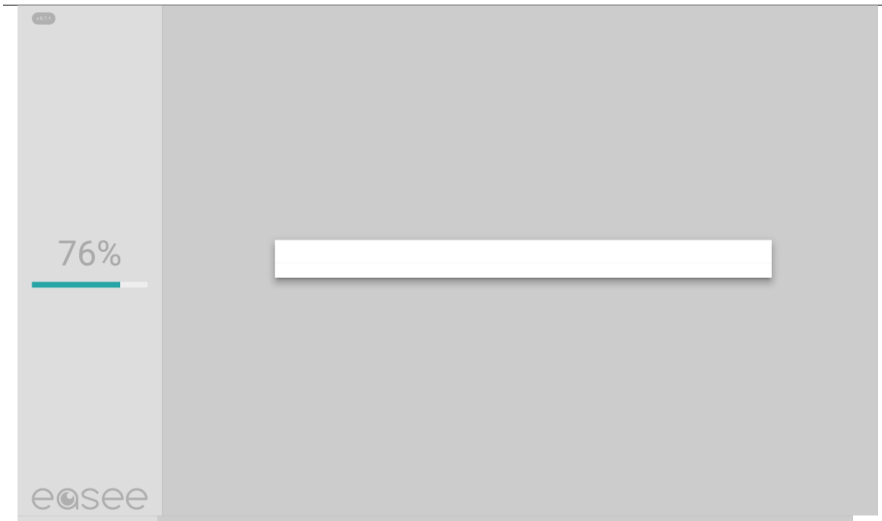
76%

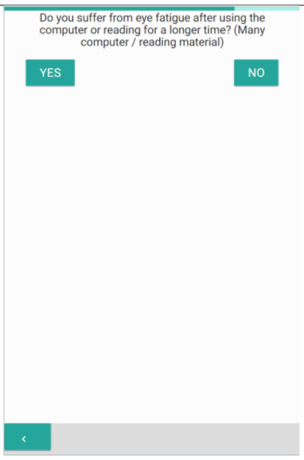
Do you suffer from eye fatigue after using the computer or reading for a longer time? (Many computer / reading material)
YES
NO

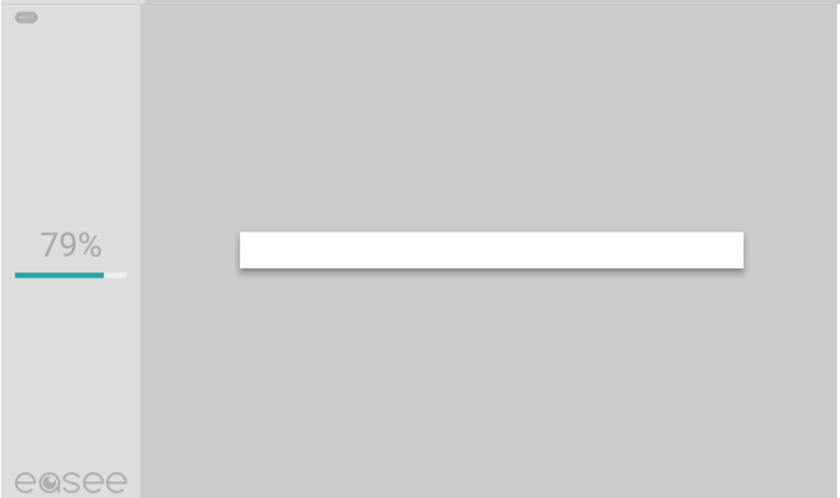
79%

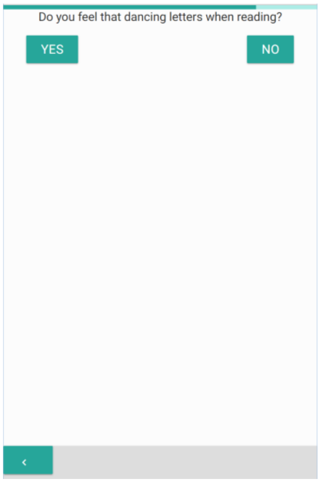
Do you feel that dancing letters when reading?
YES
NO

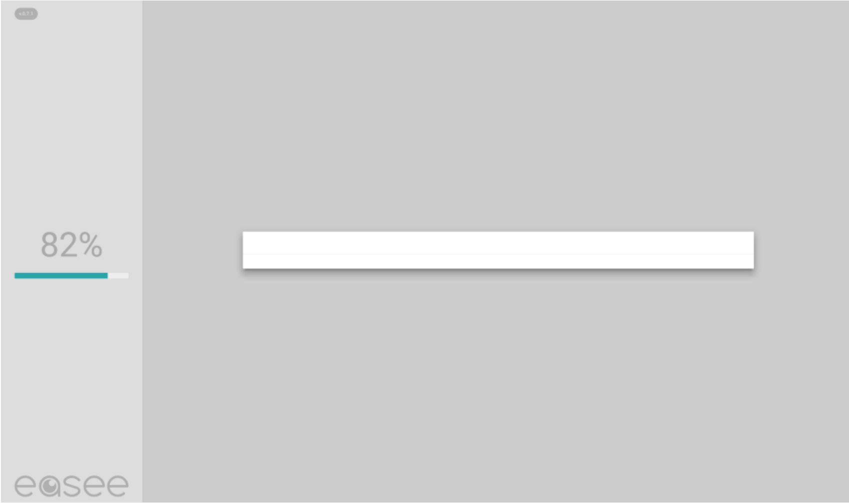
82%

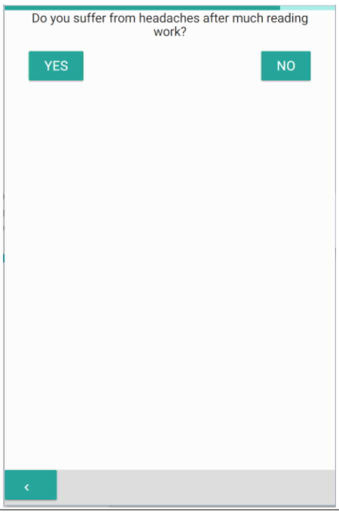
Do you suffer from headaches after much reading work?
YES
NO

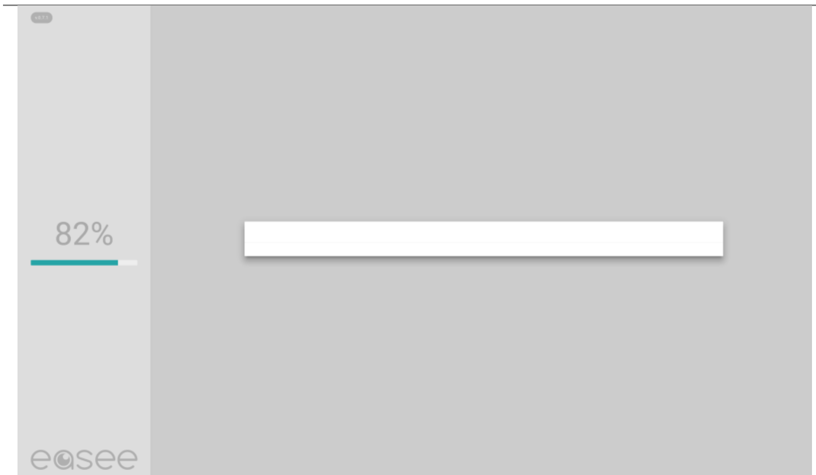

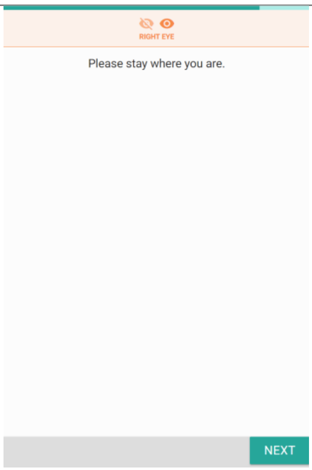

For the next part of the test the user is asked to either remain at the same distance of 1.5 meters to the screen or move towards the screen and sit in front of the computer screen, depedning on the refrative error of the user.

Taking the astigmatism exam

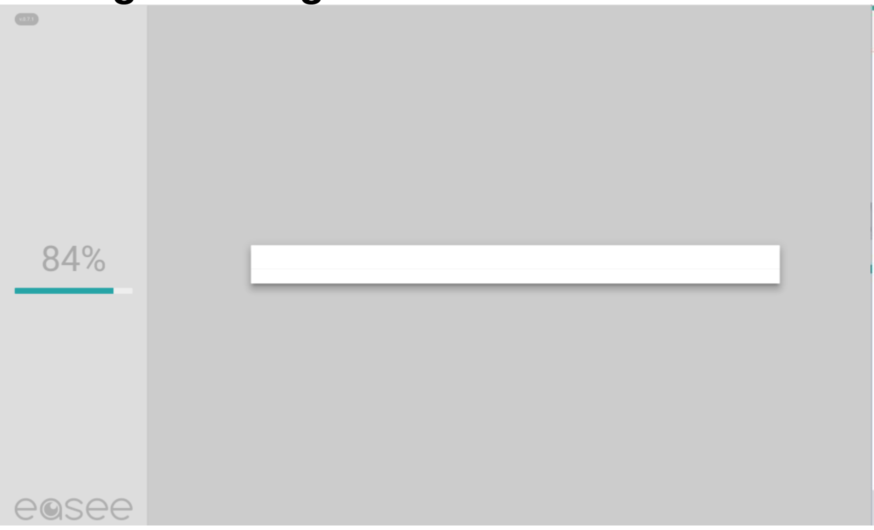

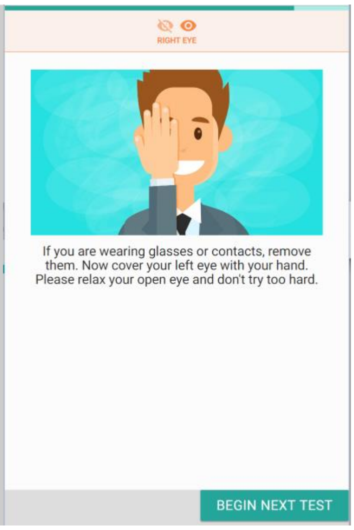

The user is now asked to cover his left eye to execute the next part of the test.

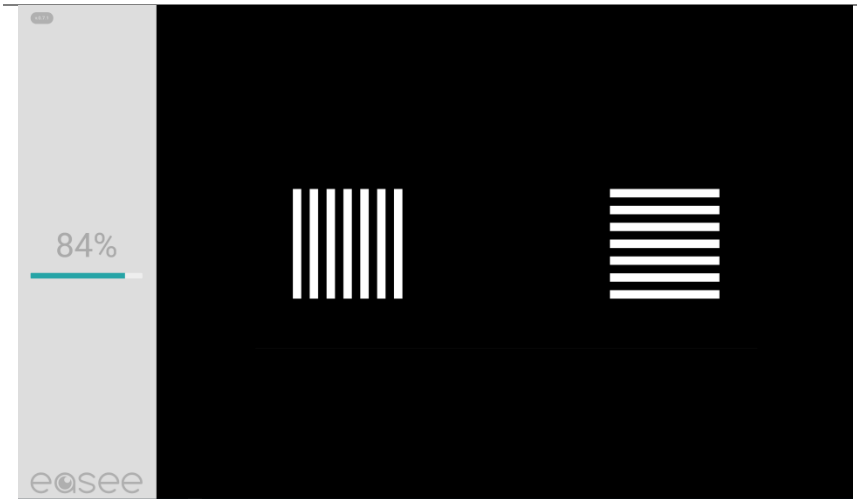

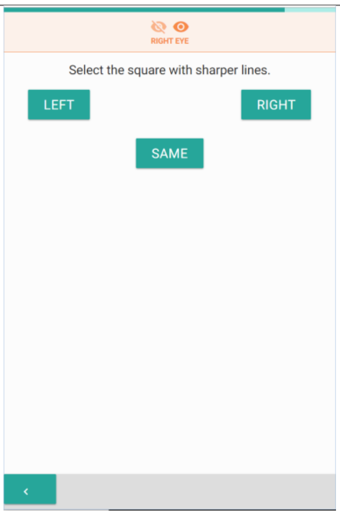

The user is presented two striped blocks that are presented at different angles, at each presentation the user is has to indicate on the phone which of the two has sharper lines, the left of the right one. If they both look the same, he can select same.

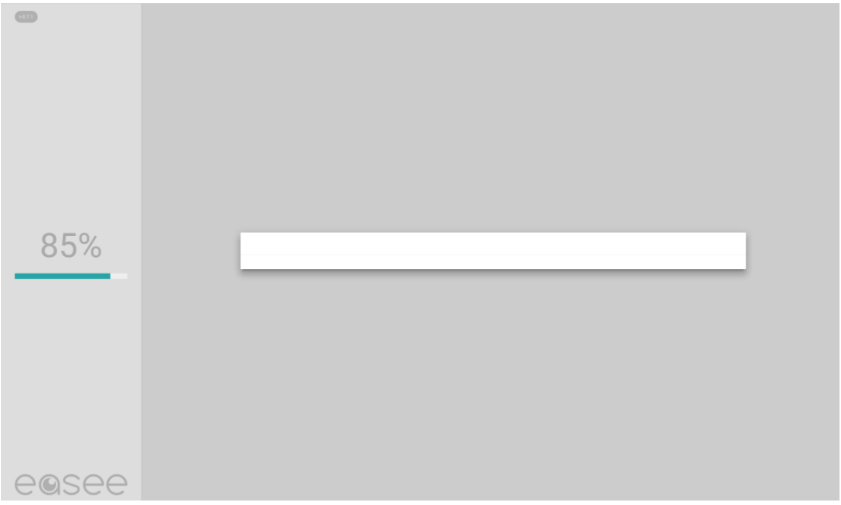

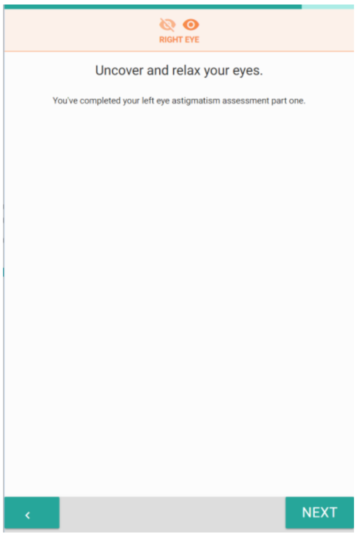

The user will be shown a screen to confirm completion of the exam for the right eye.

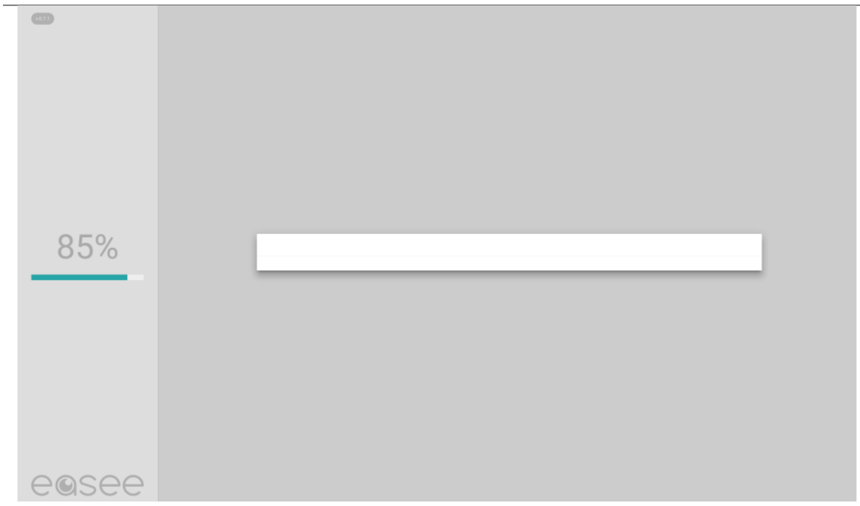

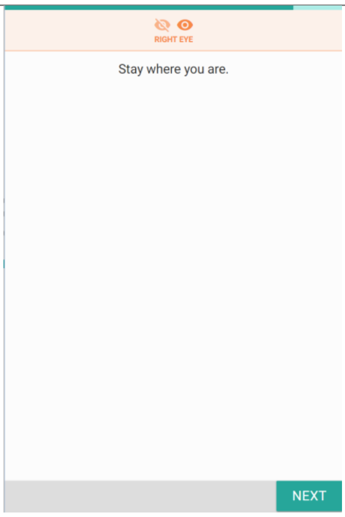

For the next part of the test the user is asked to remain at the same distance to the screen (either 1.5 meters or sitting in front of the screen)

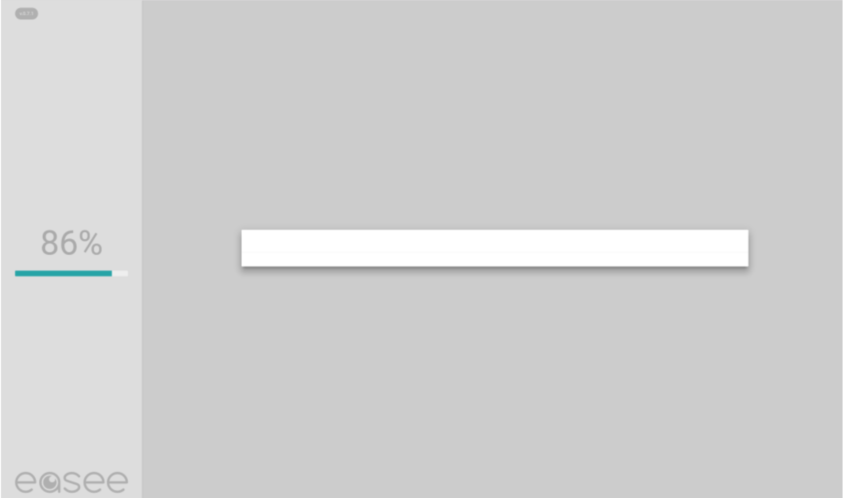

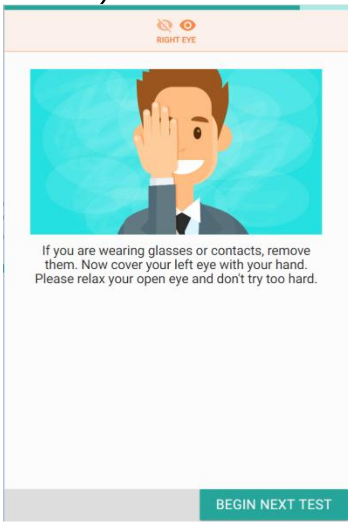

The user is now again asked to cover his left eye to execute the next part of the test.

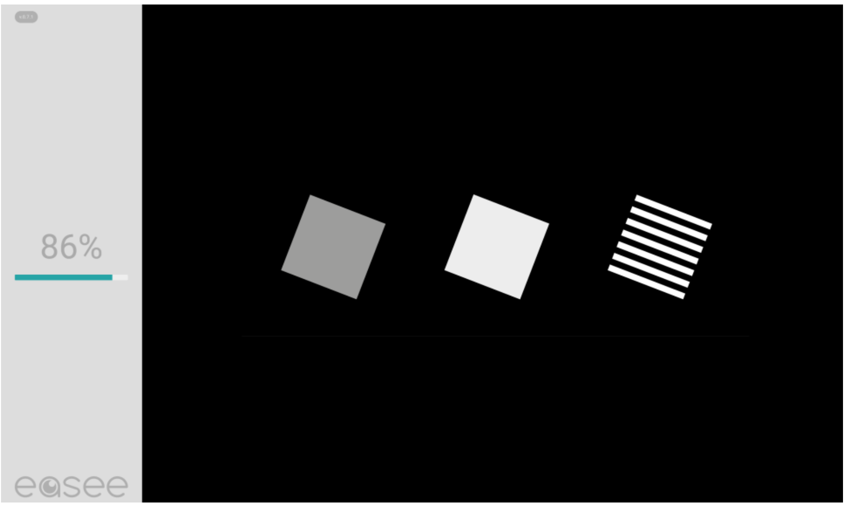

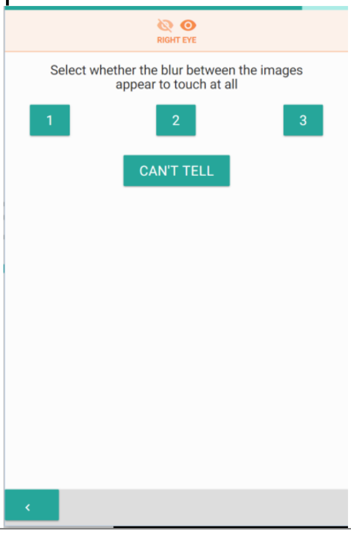

Now the user is presented 3 blocks of which one of them is striped. The user has to indicate the place of the striped block. The blocks are presented at different sizes aking it harder for the user to identify the striped block. When the user is unable to identify the striped block he can click ’can’t tell’.

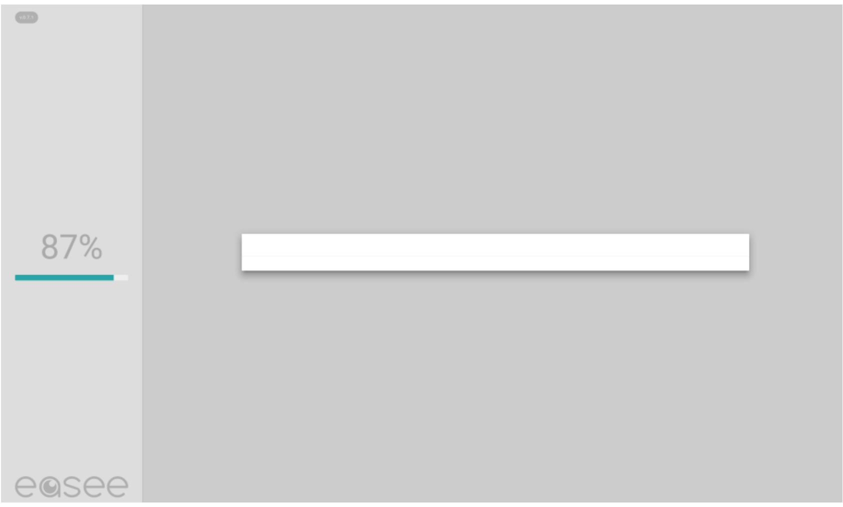

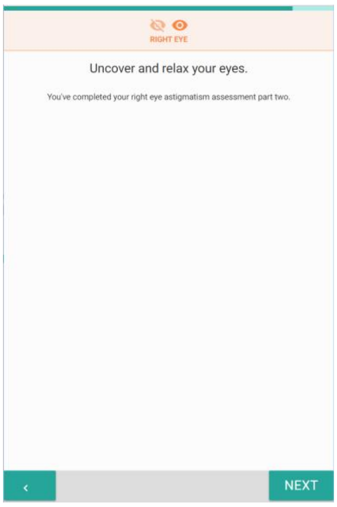

The user will be shown a screen to confirm completion of the exam for the right eye.

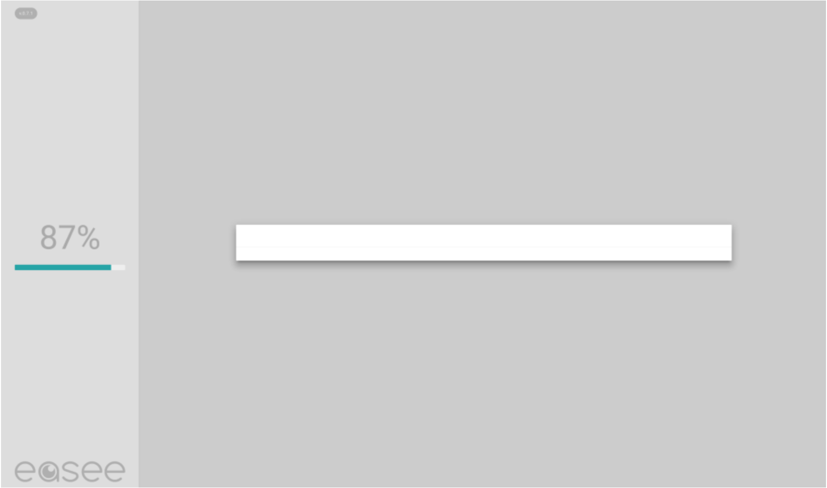

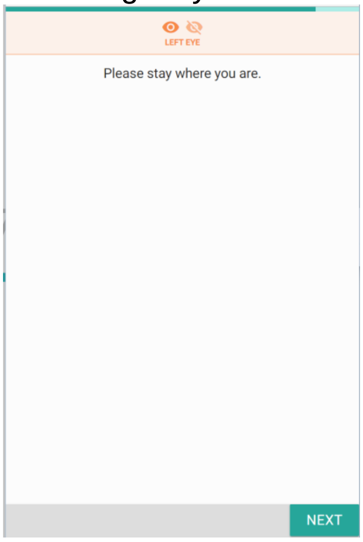

For the next part of the test the user is asked to remain at the same distance to the screen (either 1.5 meters or sitting in front of the screen)

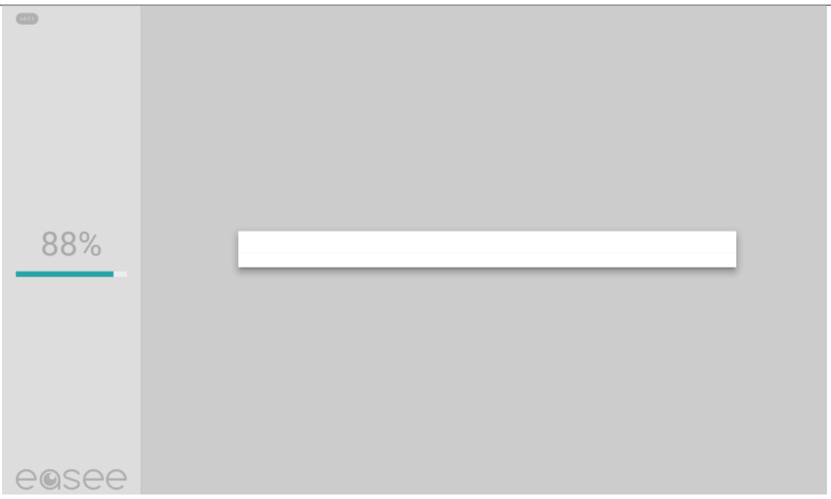

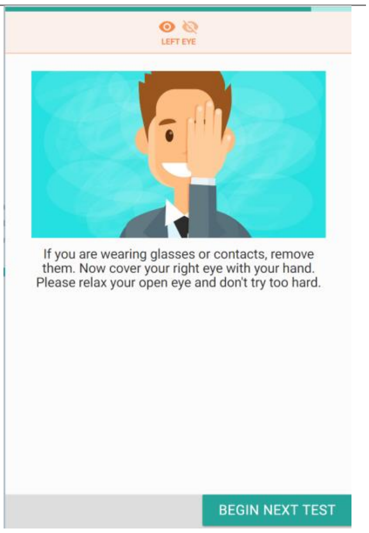

The user is now asked to cover his right eye to execute the next part of the test.

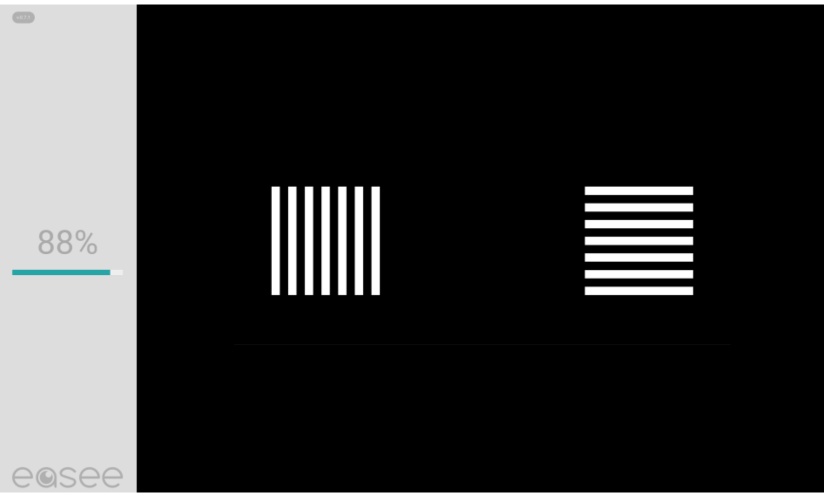

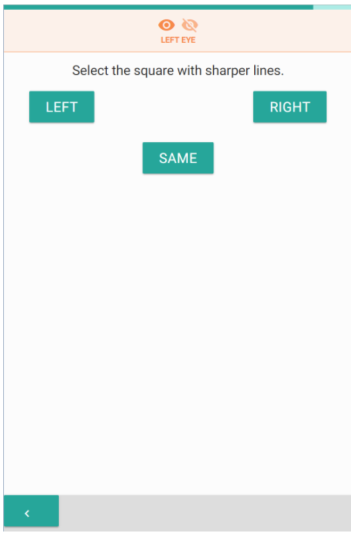

The user is presented two striped blocks that are presented at different angles, at each presentation the user is has to indicate on the phone which of the two has sharper lines, the left of the right one. If they both look the same, he can select same.

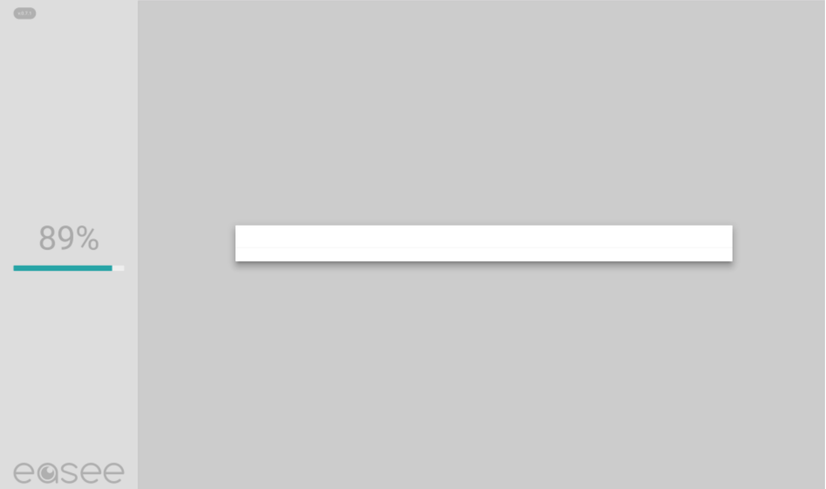

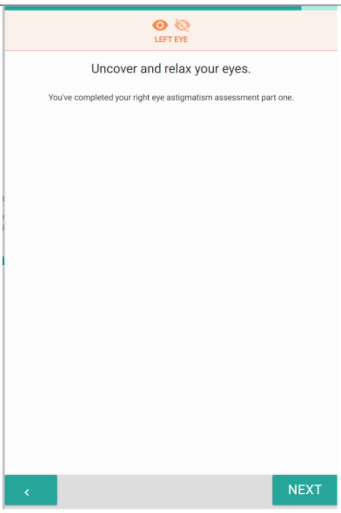

The user will be shown a screen to confirm completion of the exam for the left eye.

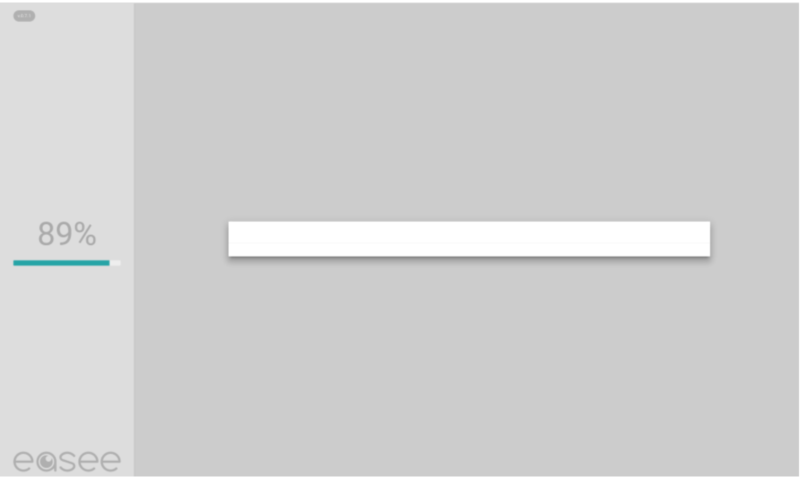

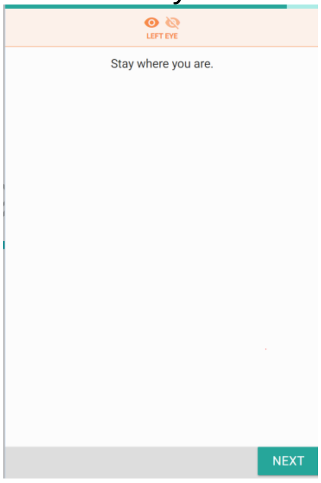

For the next part of the test the user is asked to remain at the same distance to the screen (either 1.5 meters or sitting in front of the screen)

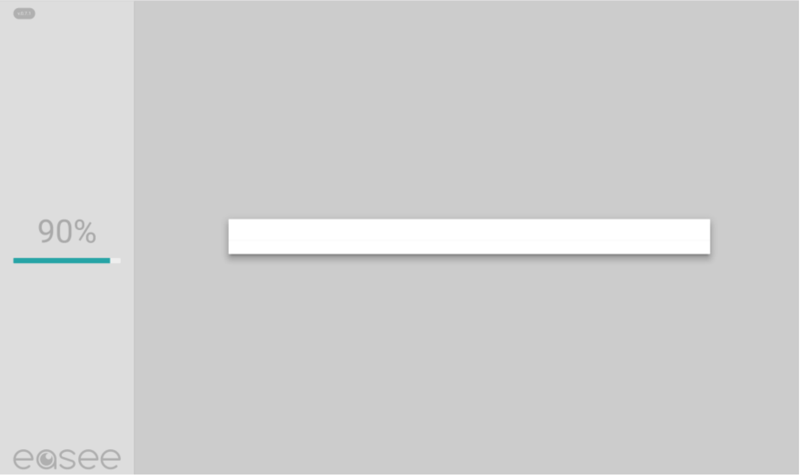

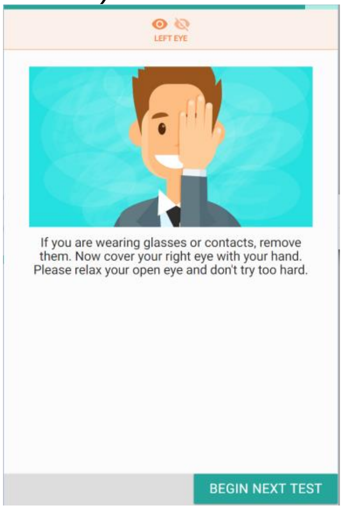

The user is now asked

to cover his right eye to execute the next part of the test.

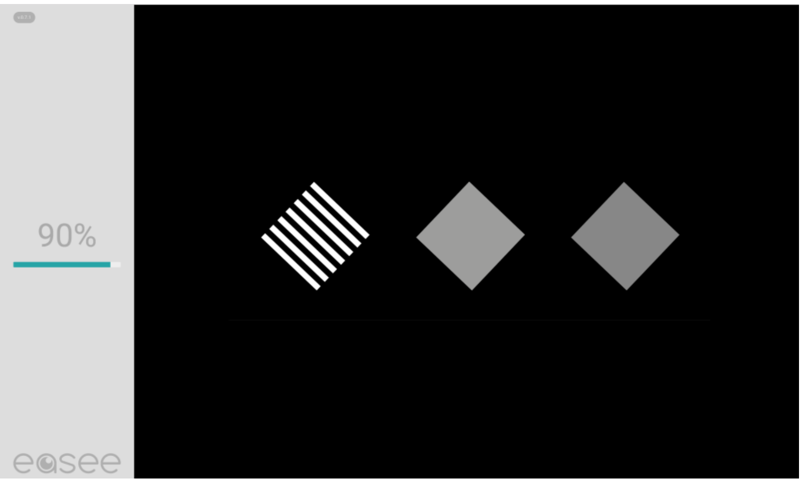

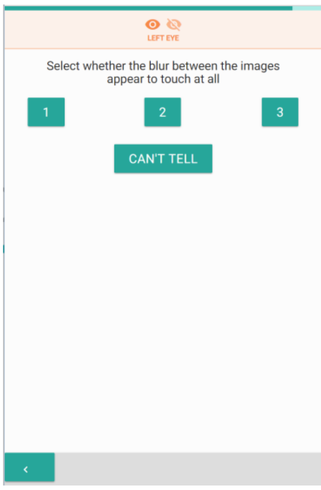

The user is again presented 3 blocks of which one of them is striped. The user has to indicate the place of the striped block. The blocks are presented at different sizes aking it harder for the user to identify the striped block. When the user is unable to identify the striped block he can click ’can’t tell’.

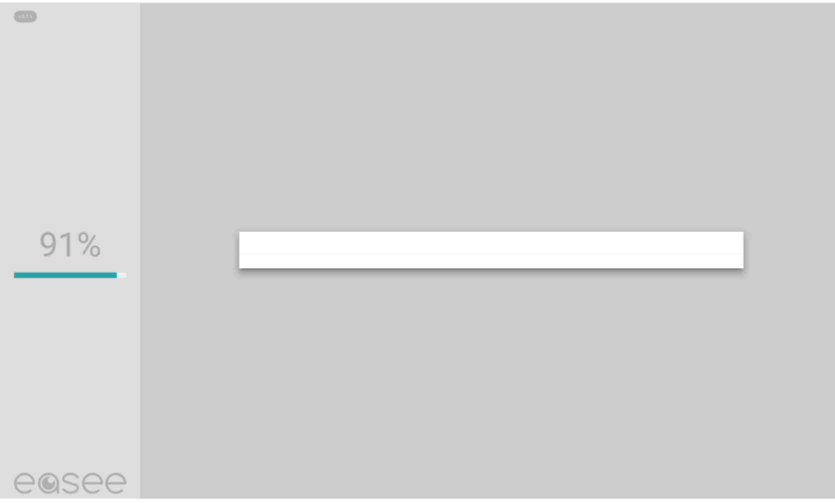

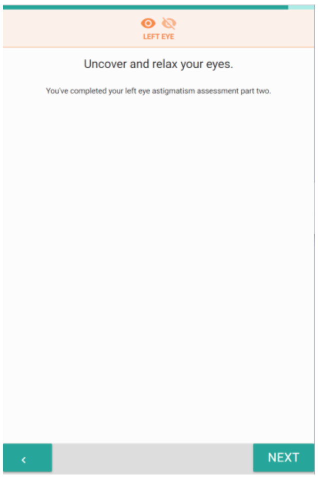

The user will be shown a screen to confirm completion of the exam for the left eye.

Viewing the exam results

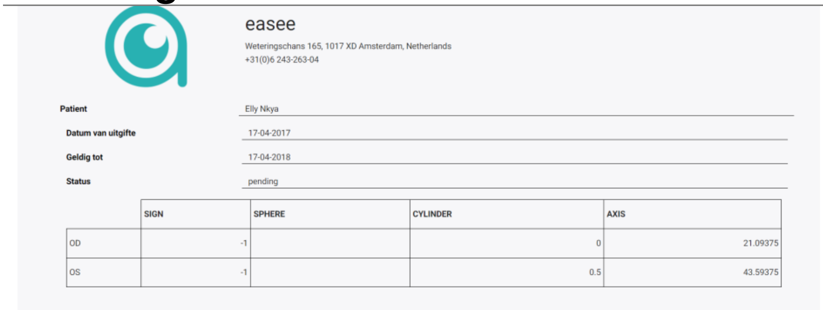
easee
Weteringschans 165, 1017 XD Amsterdam, Netherlands
+31(0)6 243-263-04

Patient	Elly Nkya
Datum van uitgifte	17-04-2017
Geldig tot	17-04-2018
Status	pending

	SIGN	SPHERE	CYLINDER	AXIS
OD	-1		0	21.09375
OS	-1		0.5	43.59375

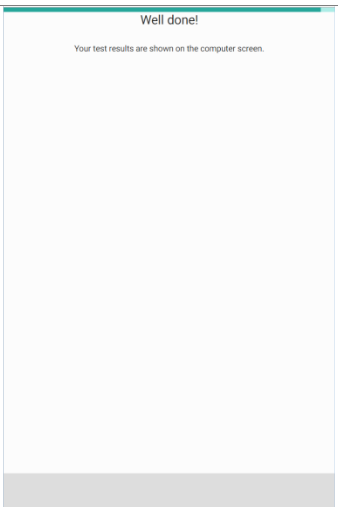

The user will be shown a screen to confirm completion of the entire exam. The results are shown on the computer screen.

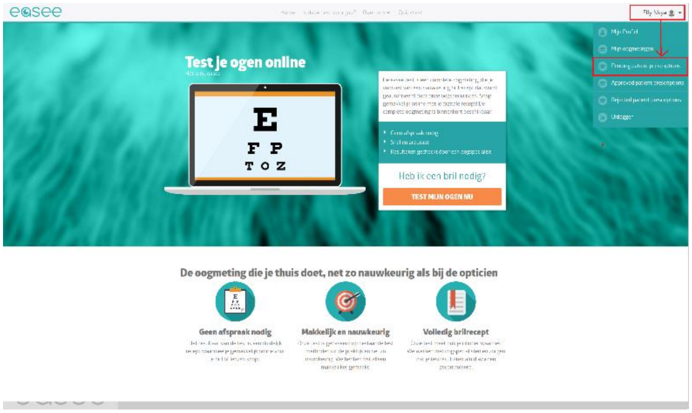

After completing the exam the doctor can view the results under pending patient prescritptions.
