# Supplementary material for: Validation of an Independent Web-Based Tool for Measuring Visual Acuity and Refractive Error (the Manifest versus Online Refractive Evaluation Trial): Prospective Open-Label Noninferiority Clinical Trial
Source: J Med Internet Res. 2019 Nov 8;21(11):e14808. doi: 10.2196/14808 (PMC6874802; doi:10.2196/14808)
Supplement: Multimedia Appendix 3 [file jmir_v21i11e14808_app3.pdf]

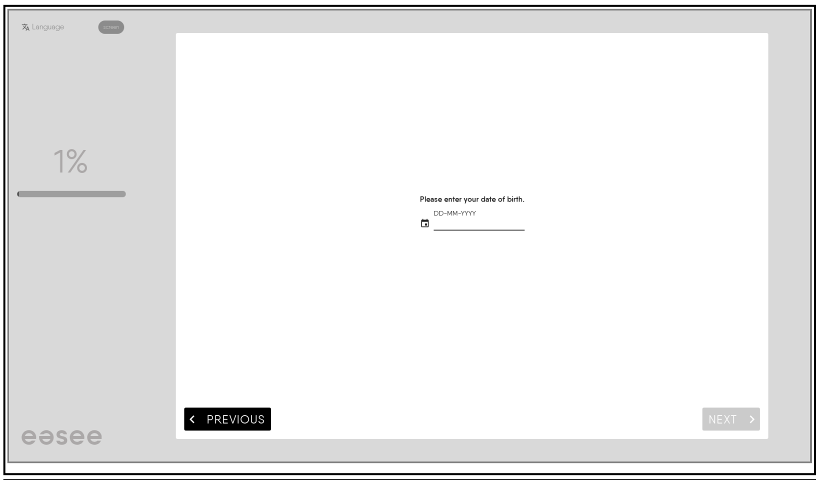
Language
1%
Please enter your date of birth.
DD-MM-YYYY
PREVIOUS
NEXT
easee

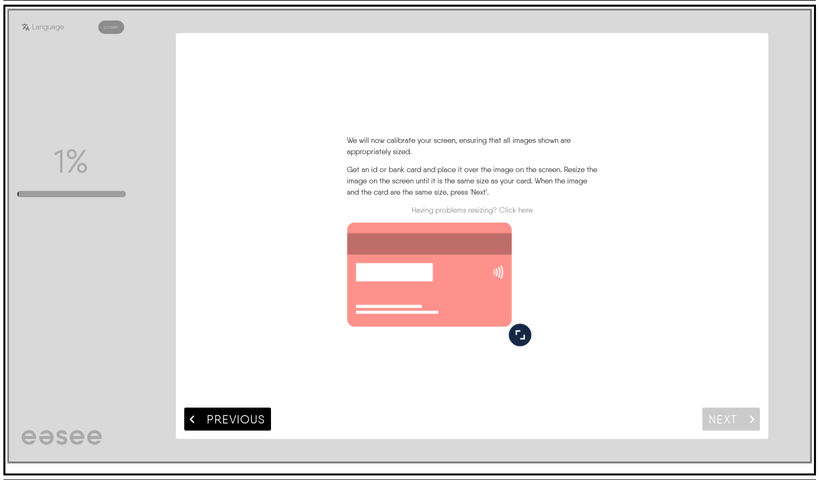
Language
1%
We will now calibrate your screen, ensuring that all images shown are appropriately sized.
Get an id or bank card and place it over the image on the screen. Resize the image on the screen until it is the same size as your card. When the image and the card are the same size, press 'Next'.
Having problems resizing? Click here
PREVIOUS
NEXT
easee

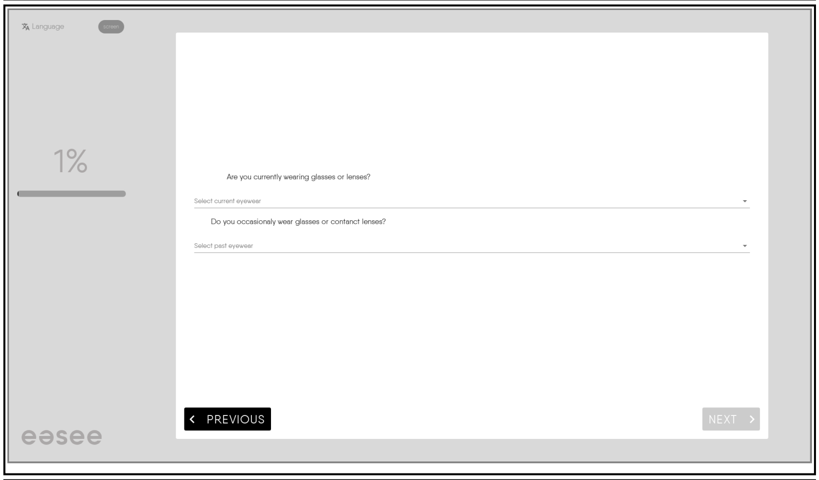
Language
1%
Are you currently wearing glasses or lenses?
Select current eyewear
Do you occasionaly wear glasses or contanct lenses?
Select past eyewear
PREVIOUS
NEXT
easee

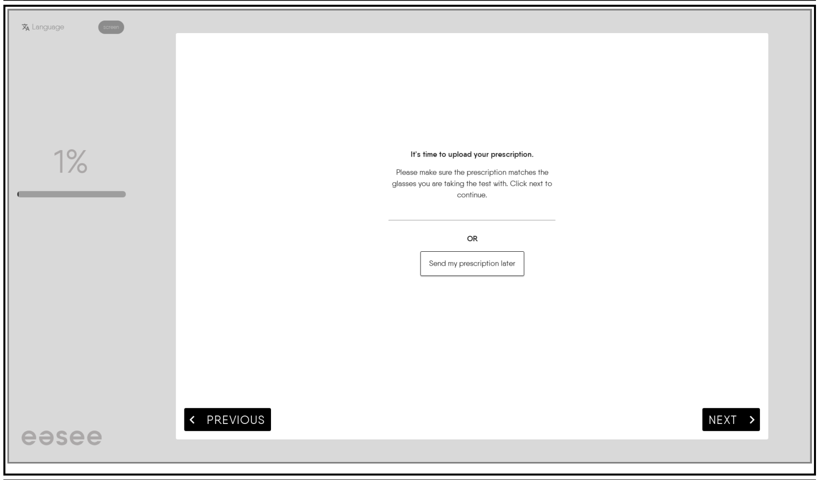
Language
1%
It's time to upload your prescription.
Please make sure the prescription matches the glasses you are taking the test with. Click next to continue.
OR
Send my prescription later
PREVIOUS
NEXT
easee

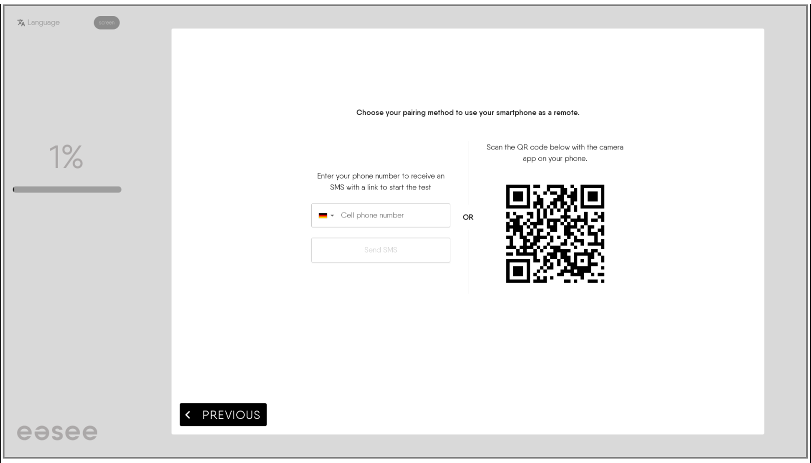
Language
1%
Choose your pairing method to use your smartphone as a remote.
Enter your phone number to receive an SMS with a link to start the test
Cell phone number
Send SMS
OR
Scan the QR code below with the camera app on your phone.
PREVIOUS
easee

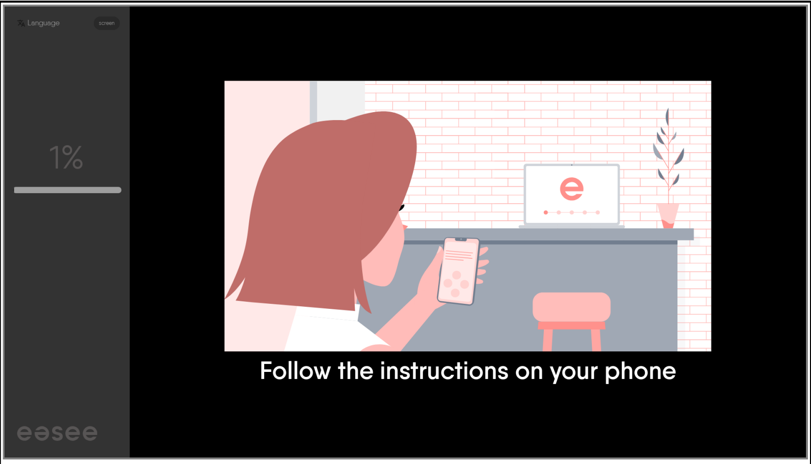
Language
1%
Follow the instructions on your phone
easee

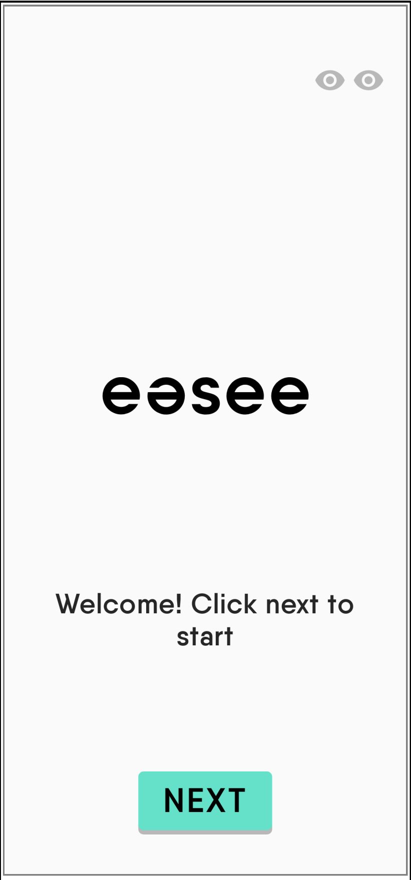
easee
Welcome! Click next to start
NEXT

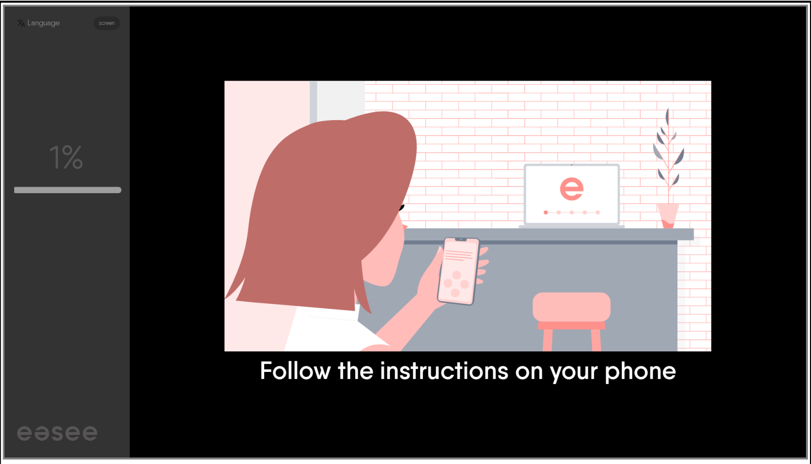
Language
1%
Follow the instructions on your phone
easee

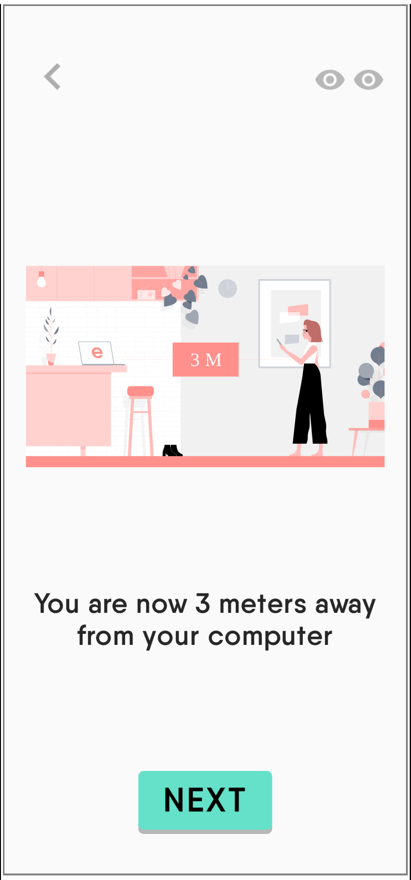
3 M
You are now 3 meters away from your computer
NEXT

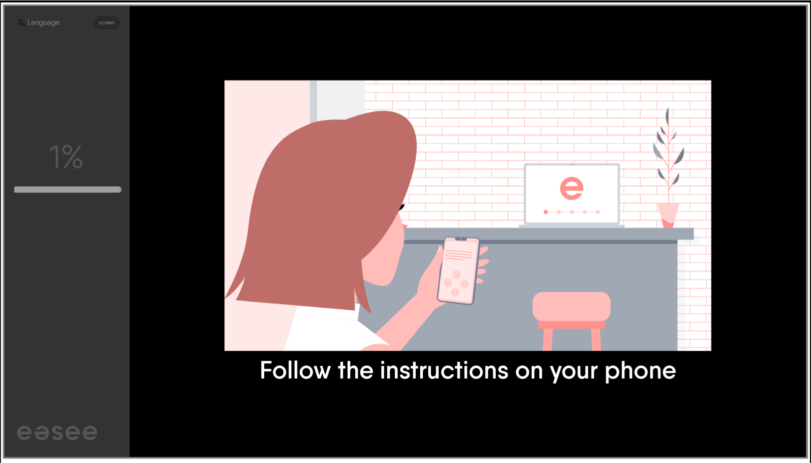
1%
Follow the instructions on your phone
easee

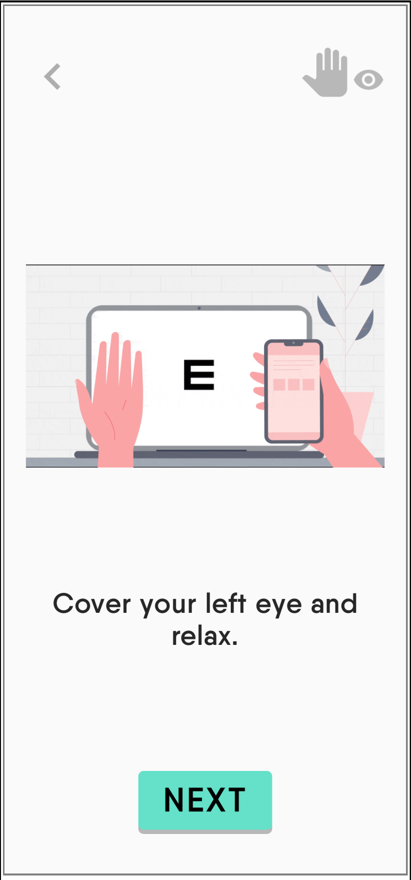
E
Cover your left eye and relax.
NEXT

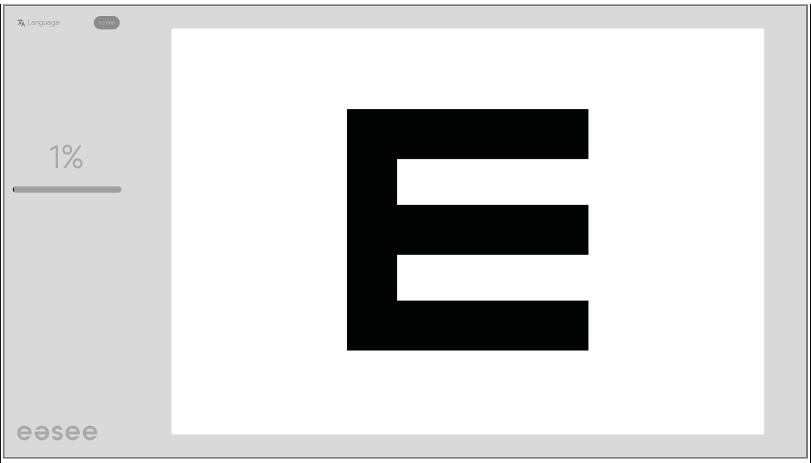
1%
easee

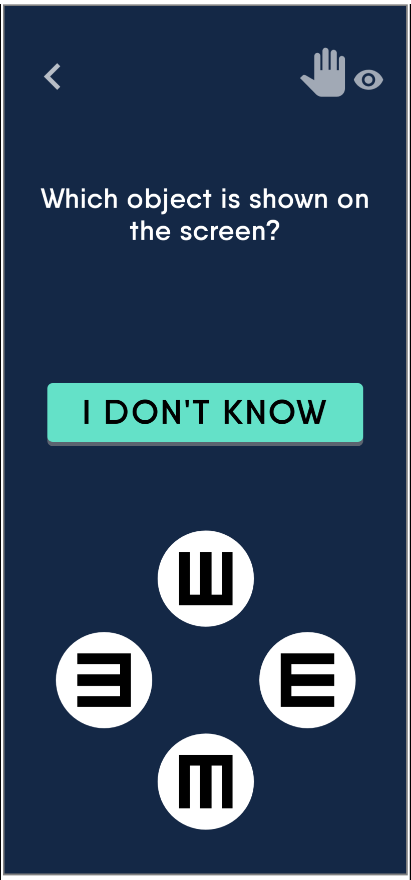
Which object is shown on the screen?
I DON'T KNOW

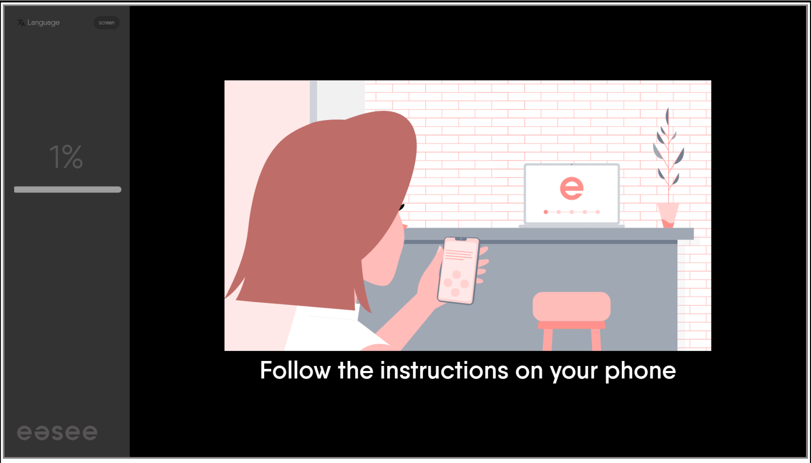
1%
Follow the instructions on your phone
easee

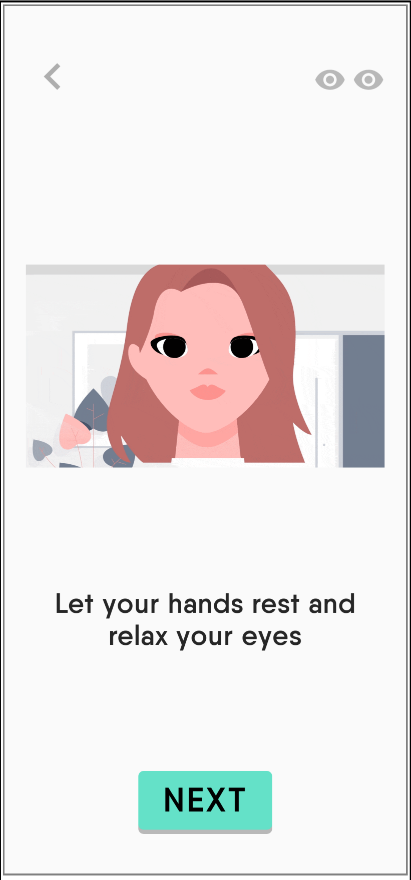
Let your hands rest and relax your eyes
NEXT

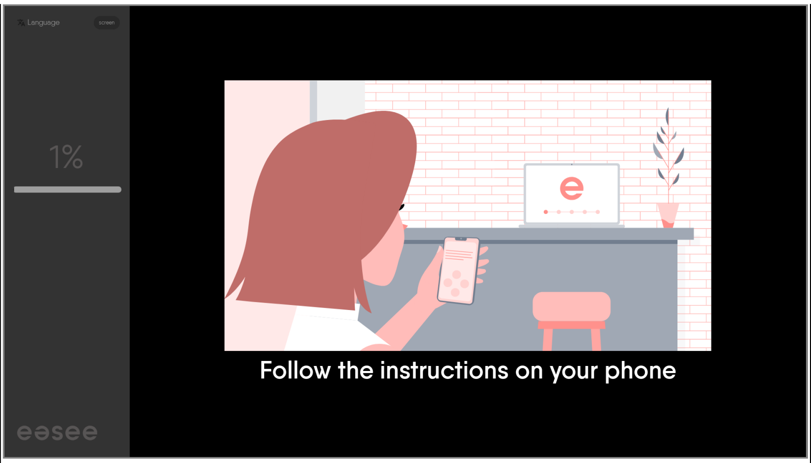
1%
Follow the instructions on your phone

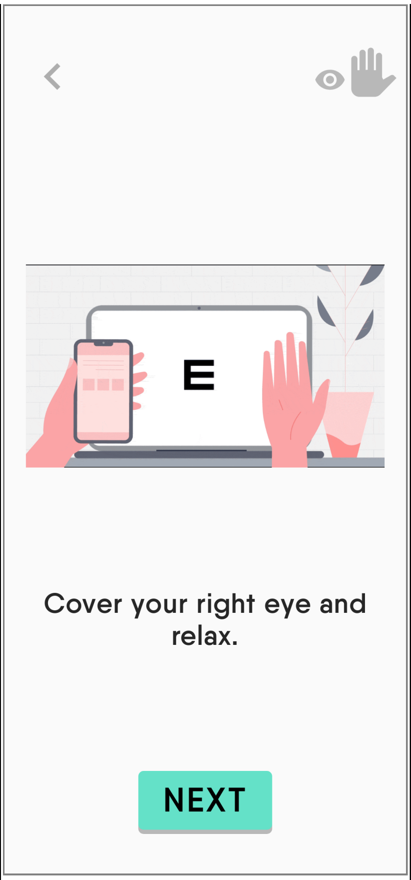
E
Cover your right eye and relax.
NEXT

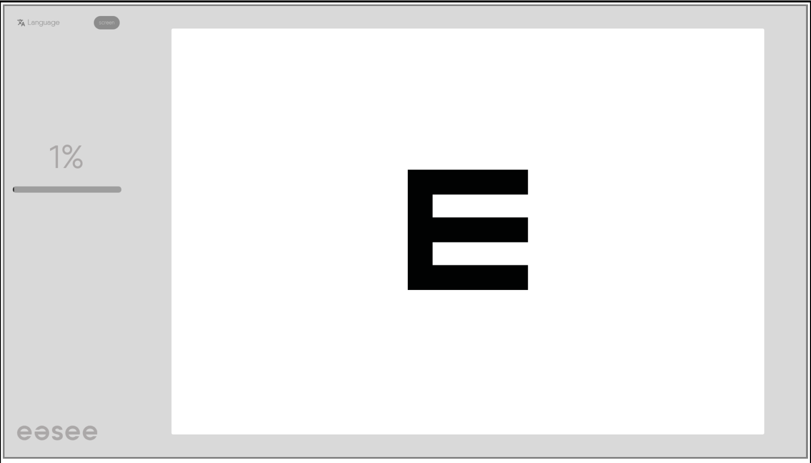
1%
E

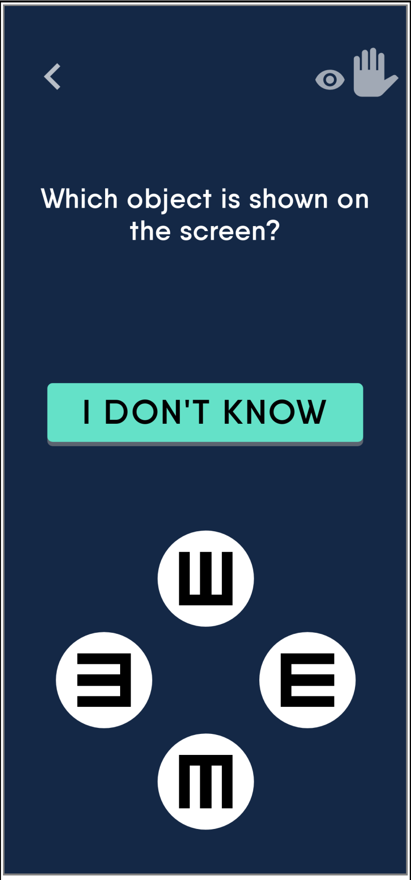
Which object is shown on the screen?
I DON'T KNOW

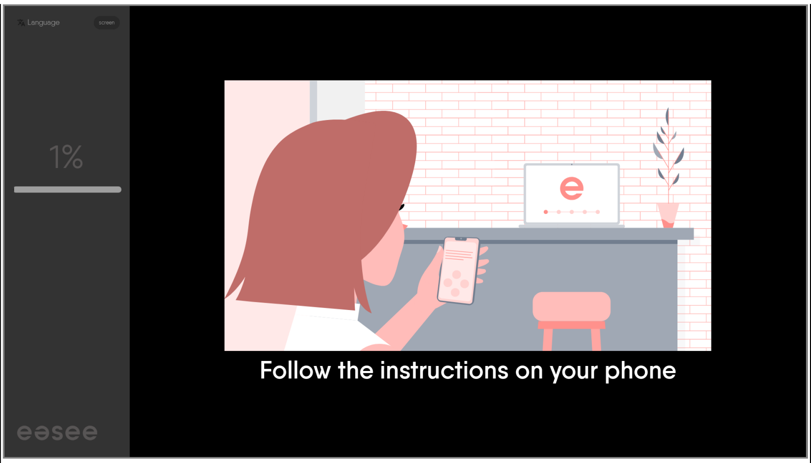1%<br>Follow the instructions on your phone	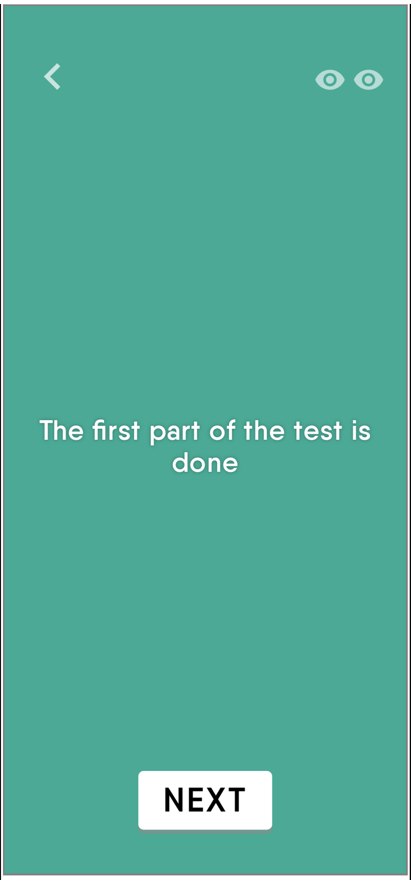The first part of the test is done<br>NEXT
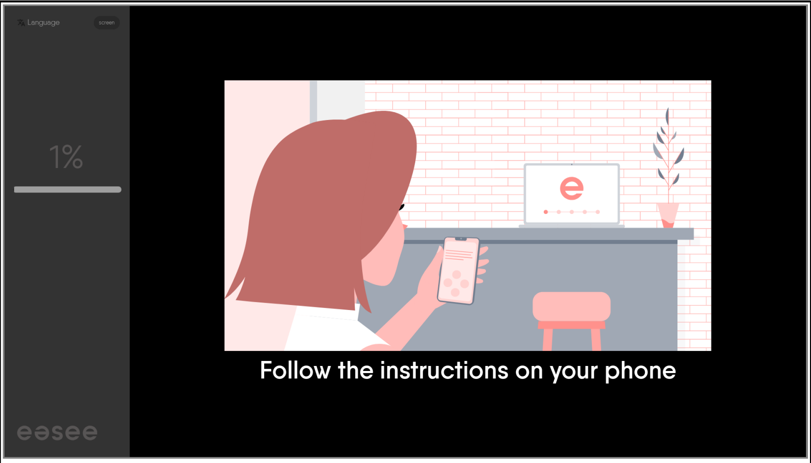1%<br>Follow the instructions on your phone	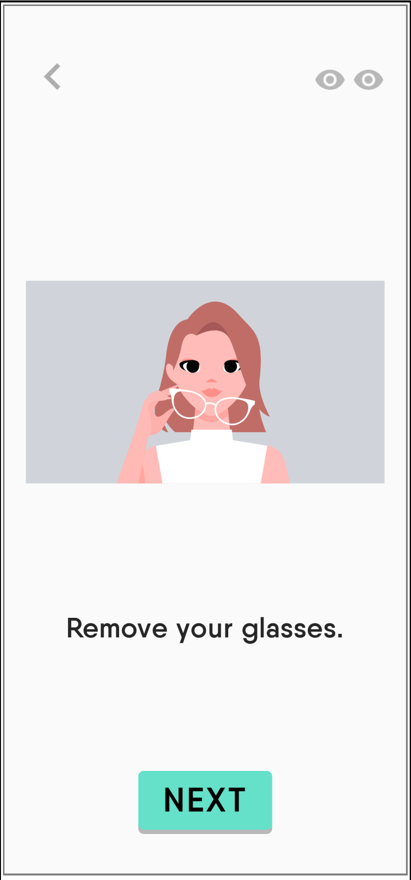Remove your glasses.<br>NEXT

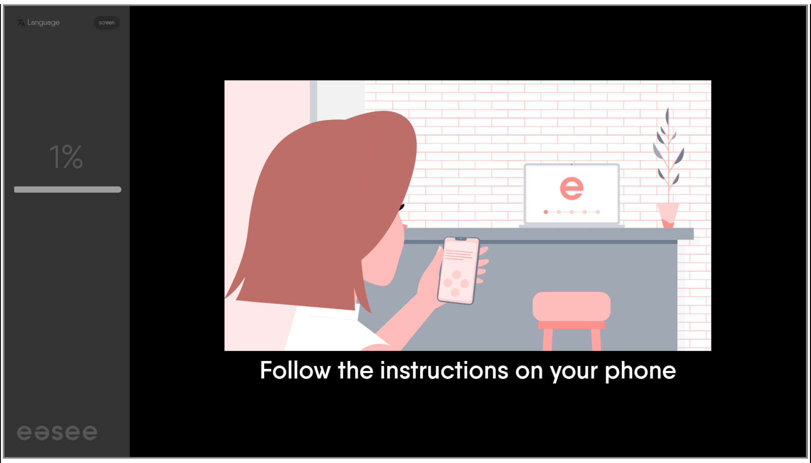
1%
easee
Follow the instructions on your phone

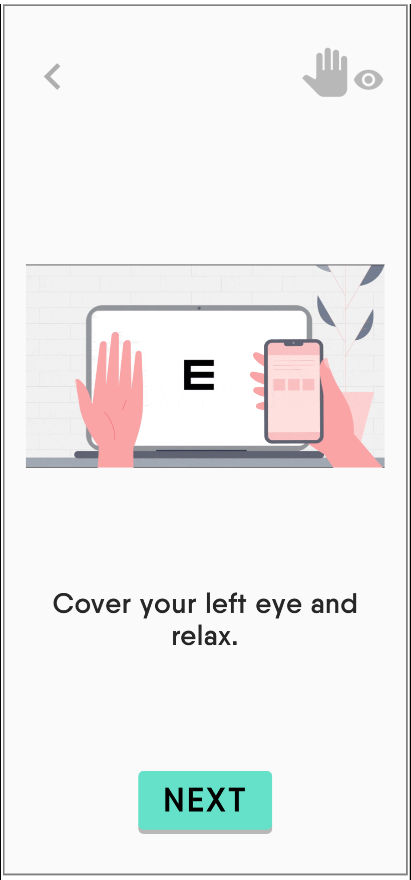
E
Cover your left eye and relax.
NEXT

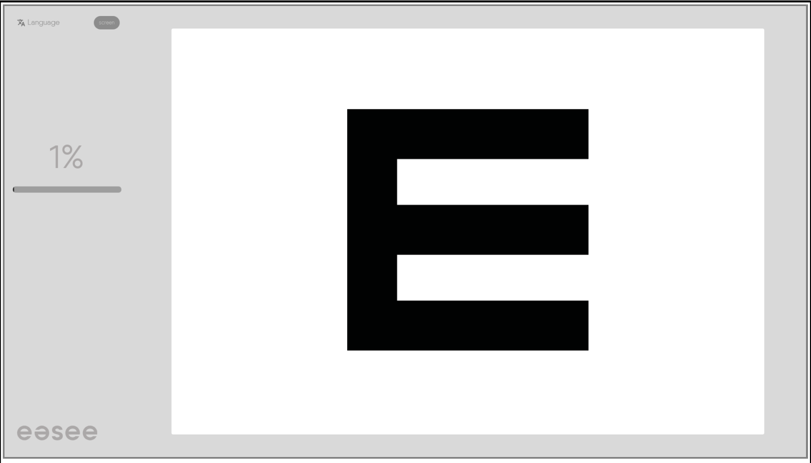
1%
easee

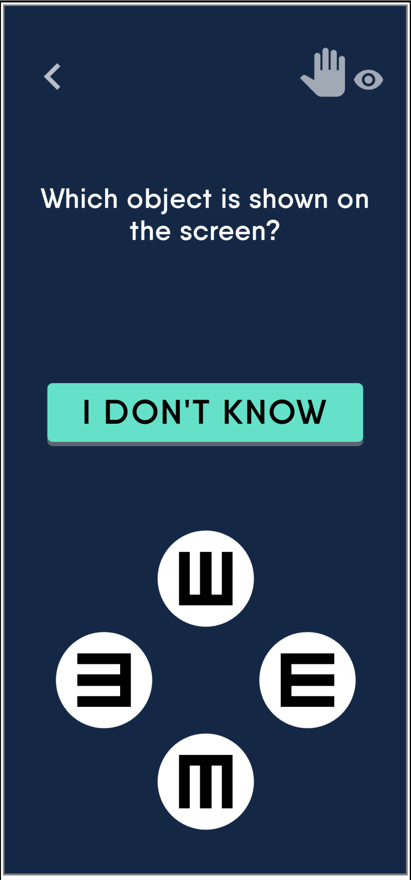
Which object is shown on the screen?
I DON'T KNOW

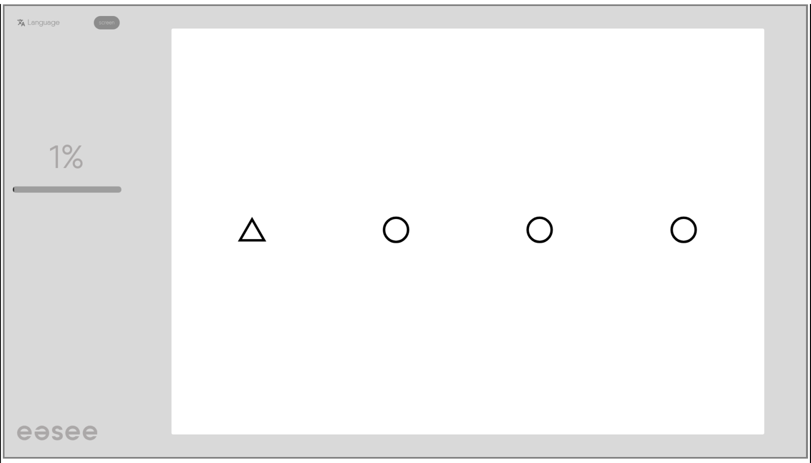
1%
easee

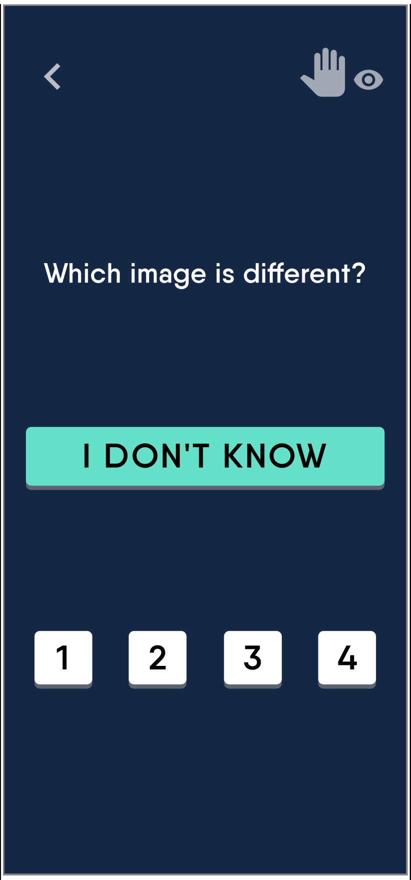
Which image is different?
I DON'T KNOW
1
2
3
4

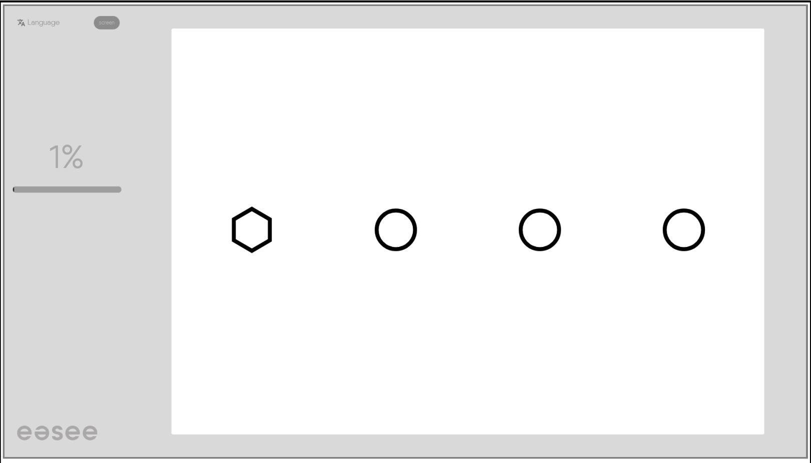
1%
easee

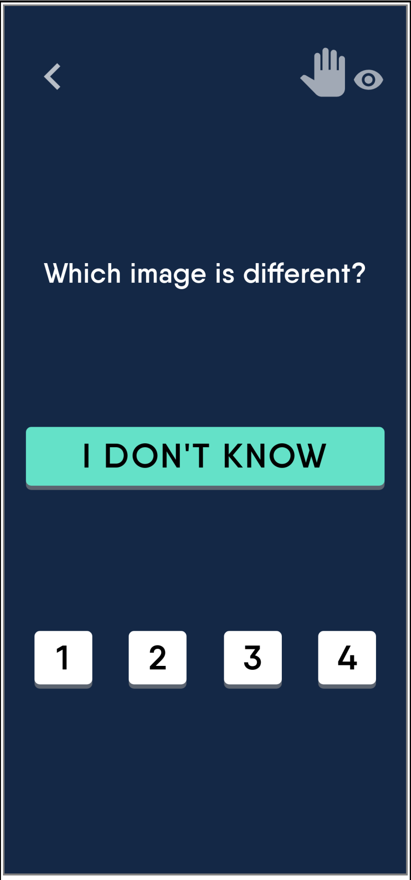
Which image is different?
I DON'T KNOW
1
2
3
4

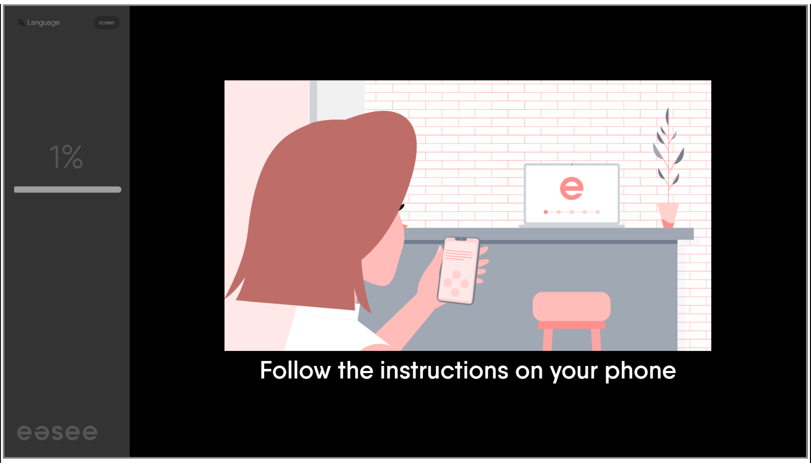
1%
easee
Follow the instructions on your phone

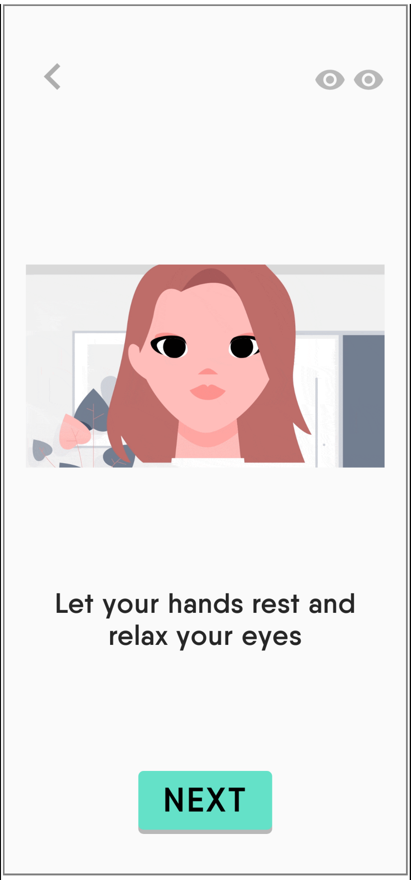
Let your hands rest and relax your eyes
NEXT

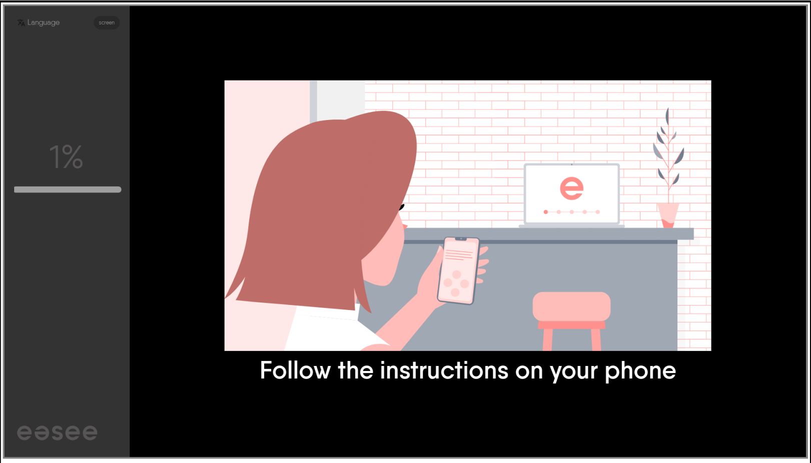
1%
easee
Follow the instructions on your phone

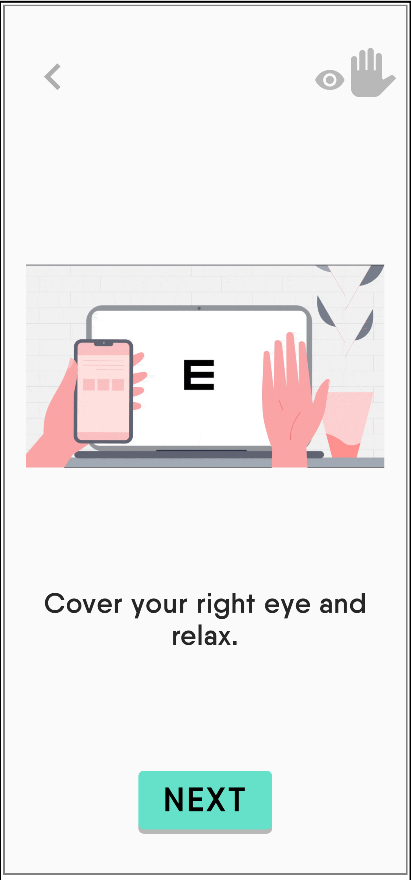
E
Cover your right eye and relax.
NEXT

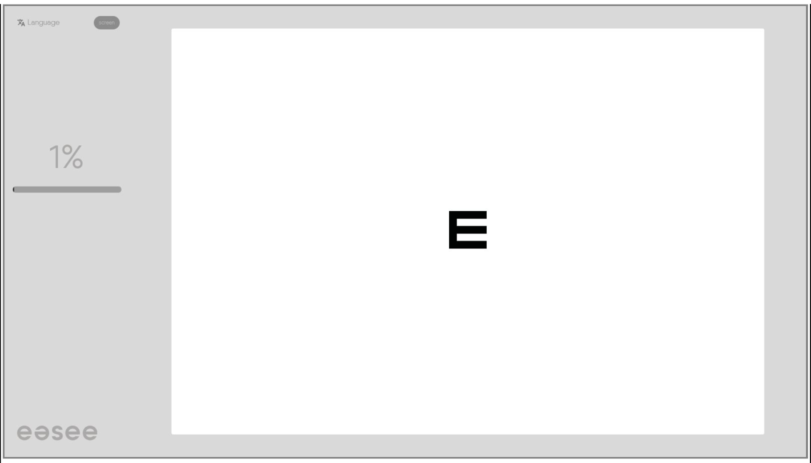
1%
E
easee

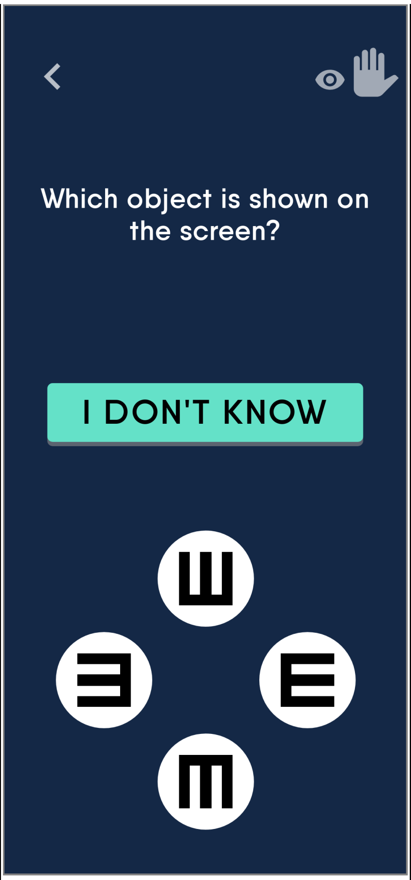
Which object is shown on the screen?
I DON'T KNOW

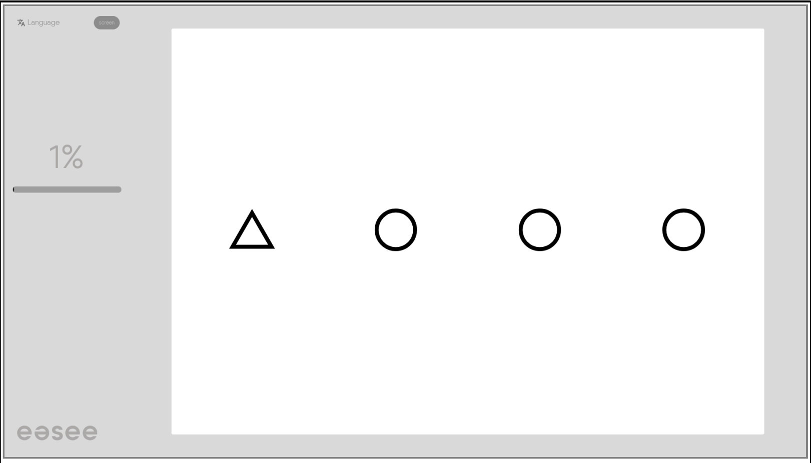
1%
easee

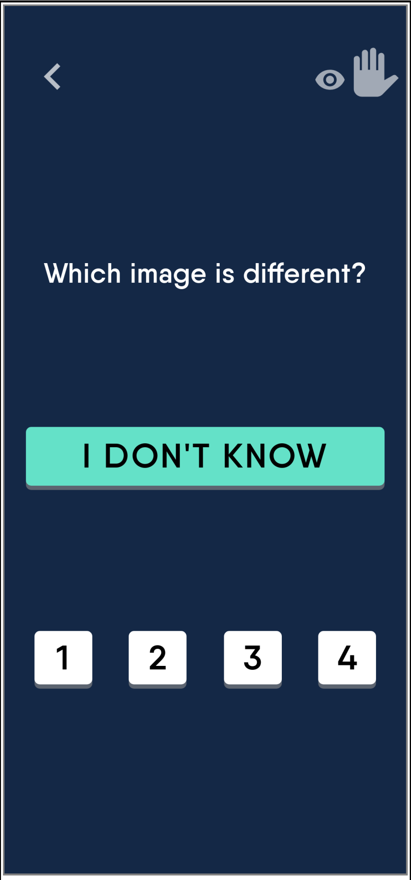
Which image is different?
I DON'T KNOW
1
2
3
4

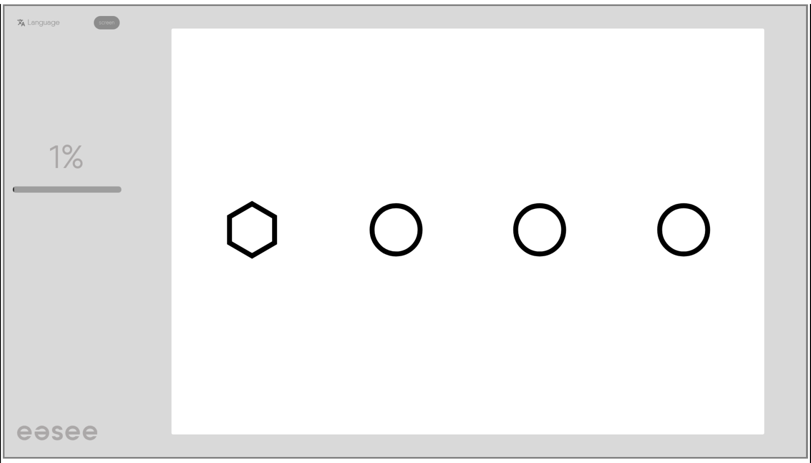
1%
easee

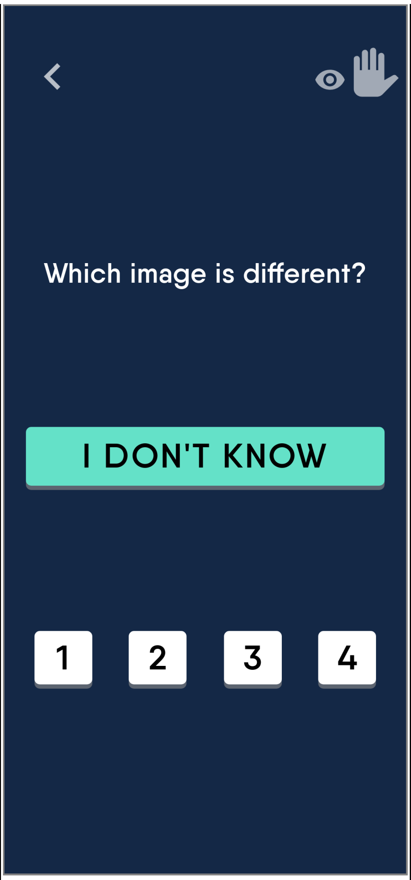
Which image is different?
I DON'T KNOW
1
2
3
4

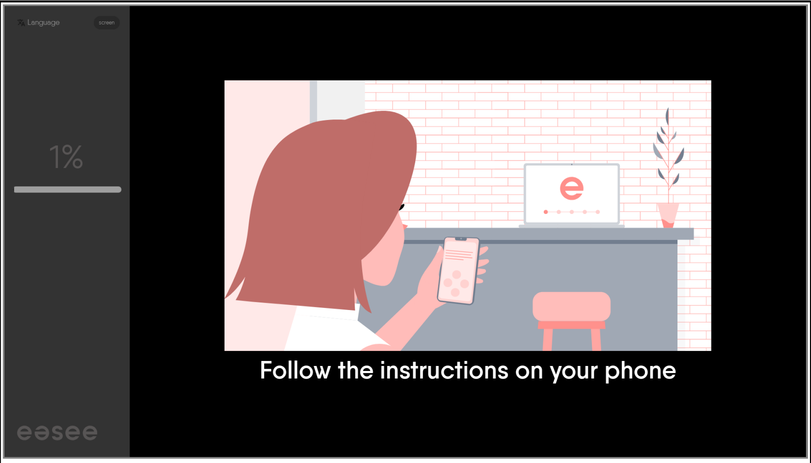
1%
Follow the instructions on your phone
easee

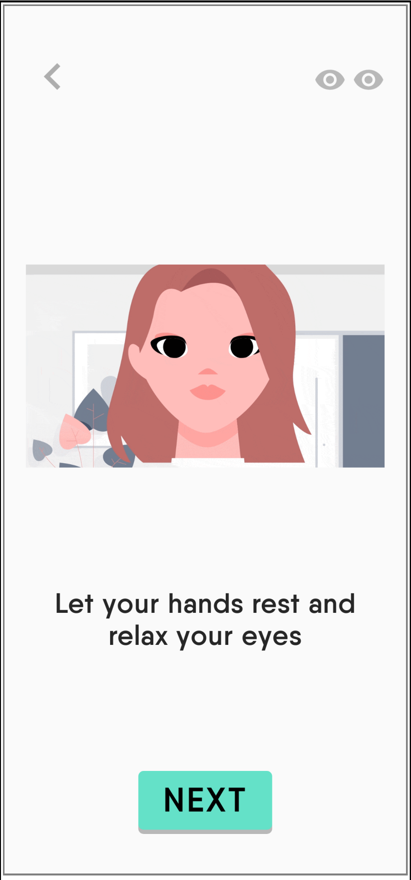
Let your hands rest and relax your eyes
NEXT

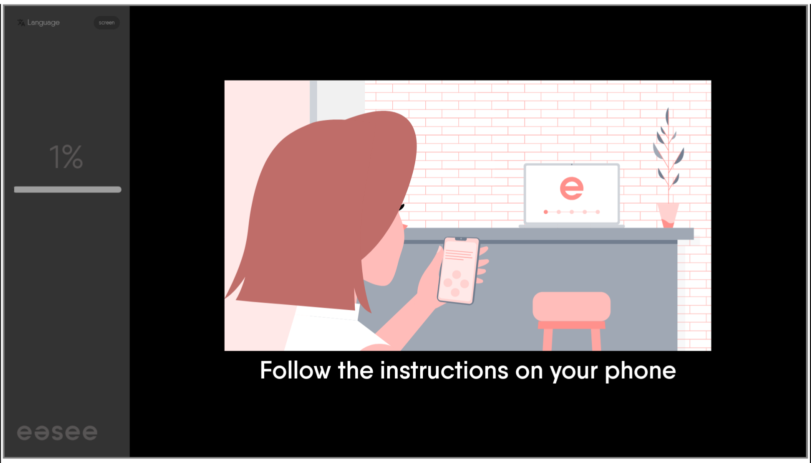
1%
easee
Follow the instructions on your phone

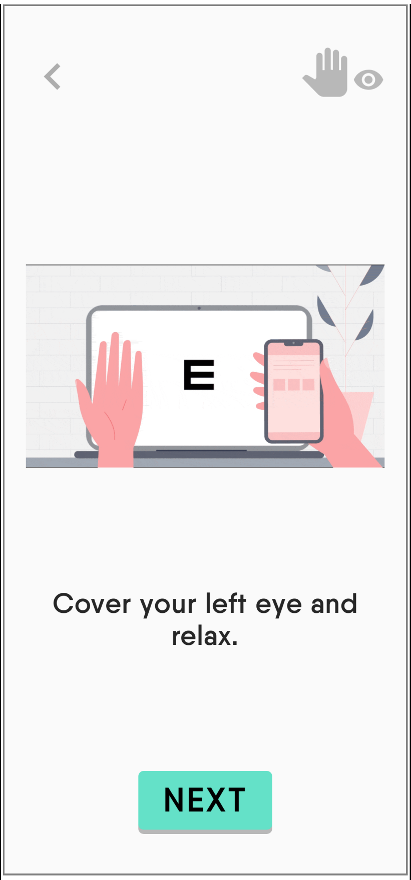
E
Cover your left eye and relax.
NEXT

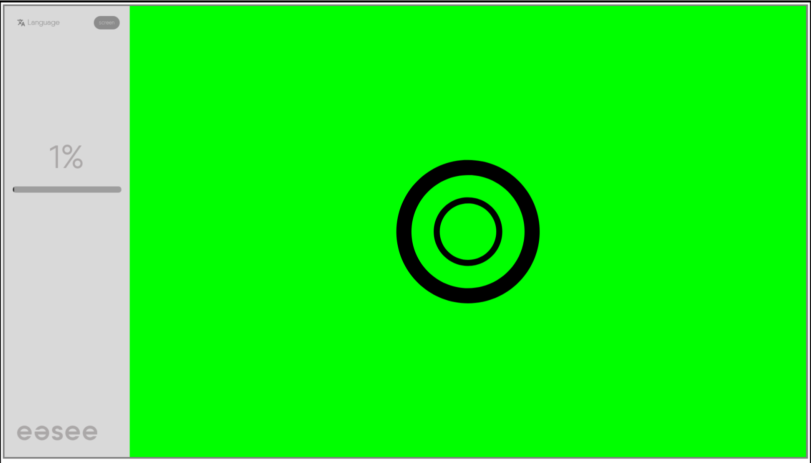
1%
easee

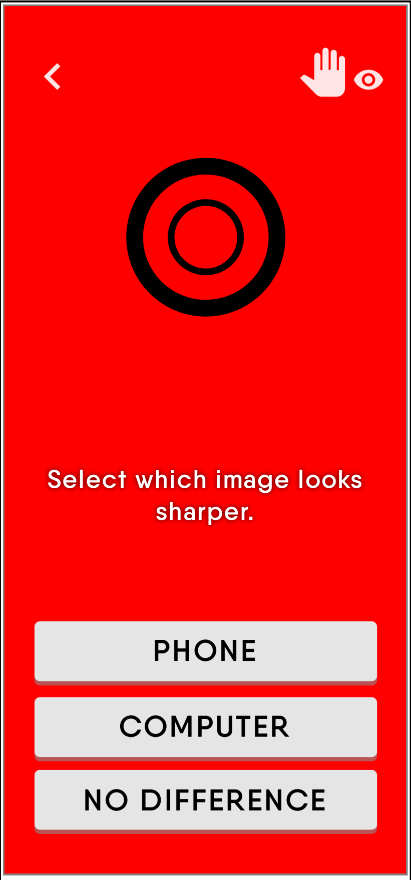
Select which image looks sharper.
PHONE
COMPUTER
NO DIFFERENCE

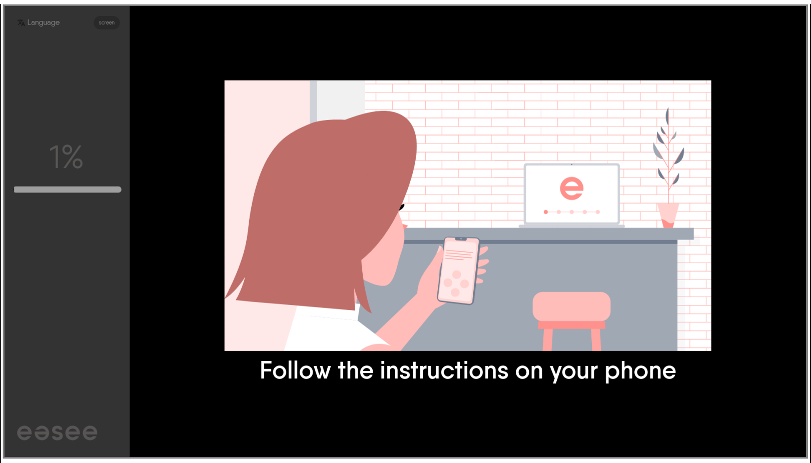
1%
easee
Follow the instructions on your phone

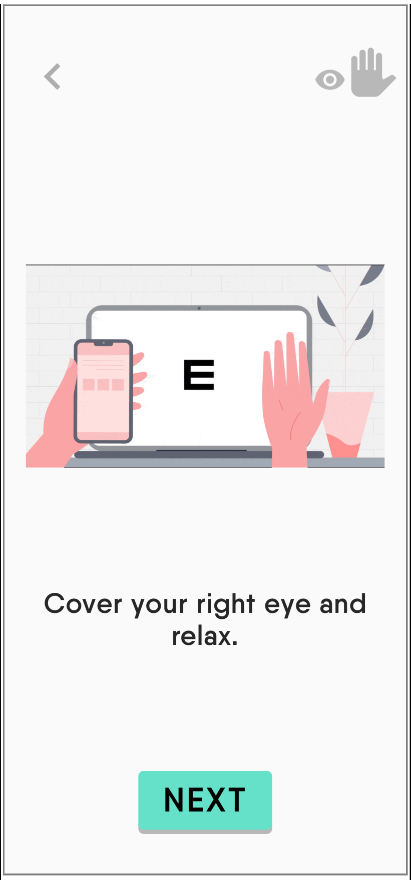
E
Cover your right eye and relax.
NEXT

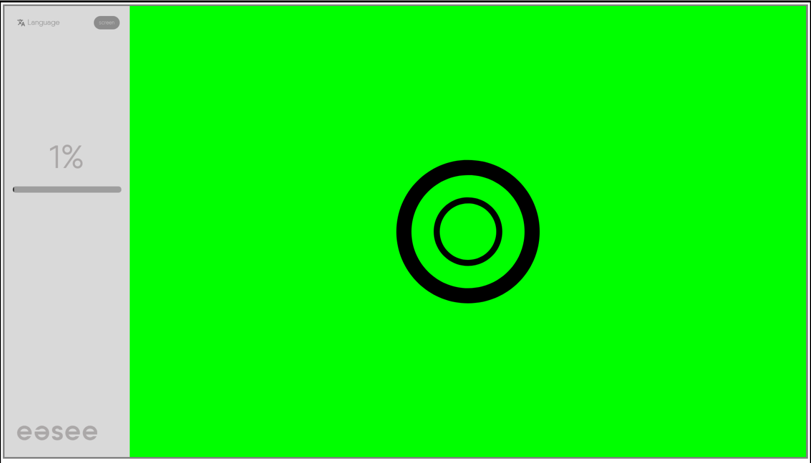
1%
easee

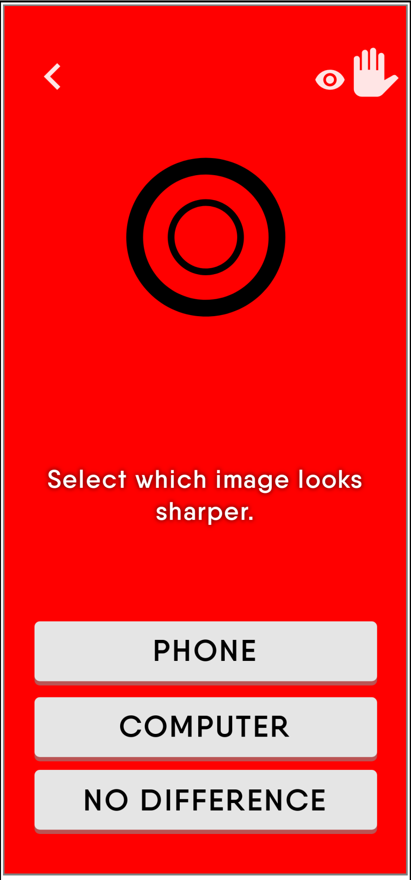
Select which image looks sharper.
PHONE
COMPUTER
NO DIFFERENCE

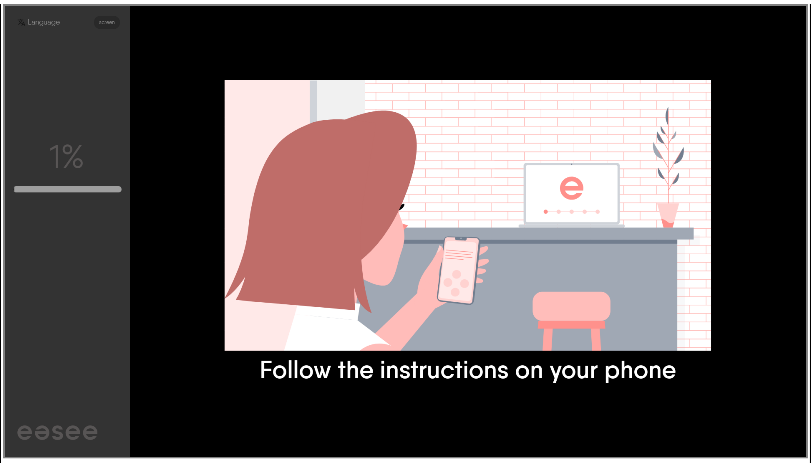
1%
Follow the instructions on your phone
easee

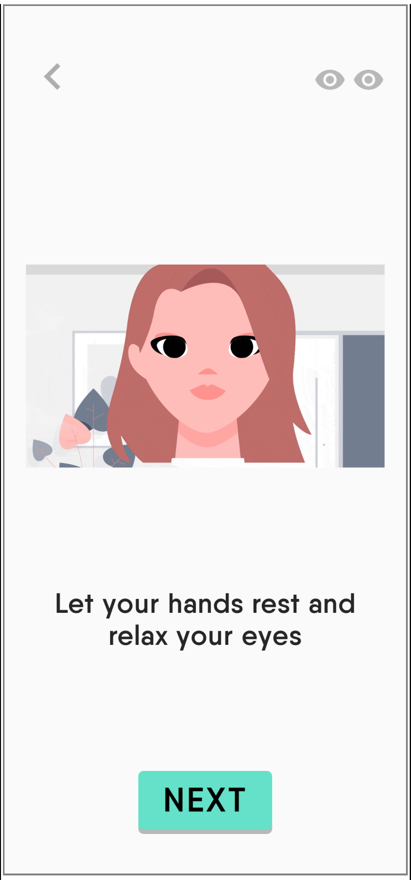
Let your hands rest and relax your eyes
NEXT

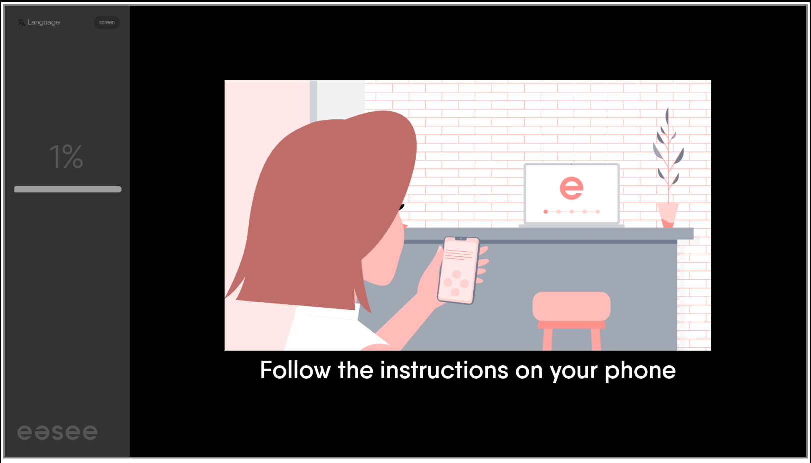
1%
Follow the instructions on your phone
easee

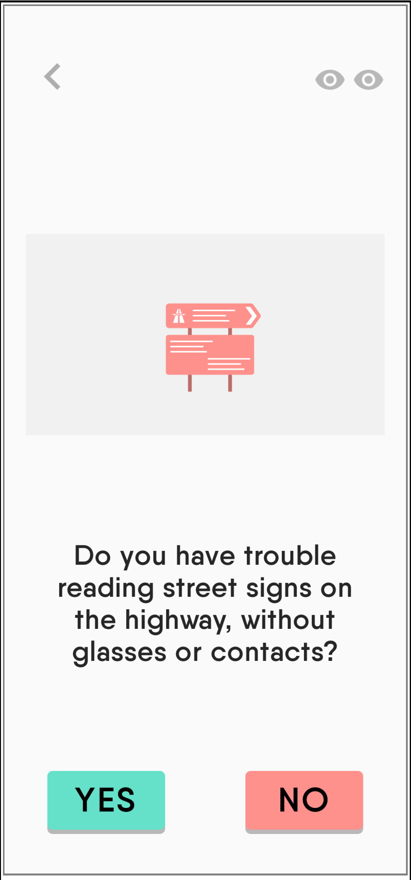
Do you have trouble reading street signs on the highway, without glasses or contacts?
YES
NO

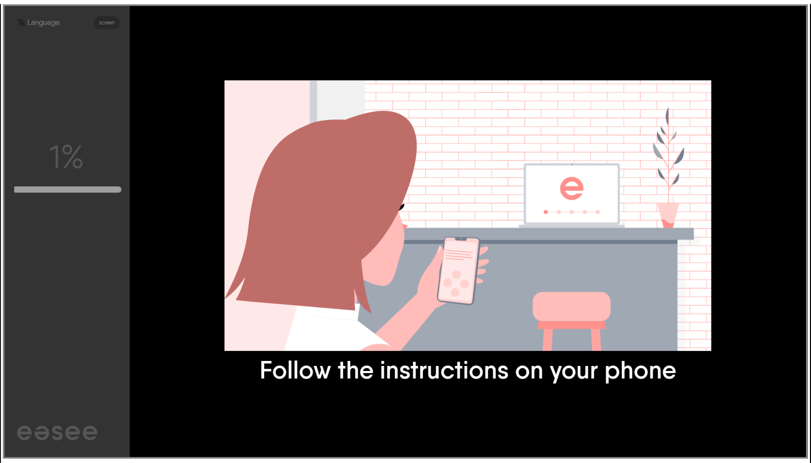
1%
Follow the instructions on your phone
easee

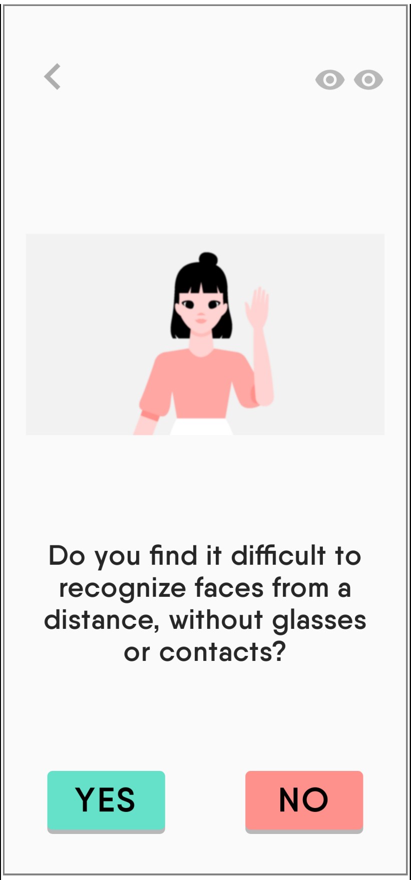
Do you find it difficult to recognize faces from a distance, without glasses or contacts?
YES
NO

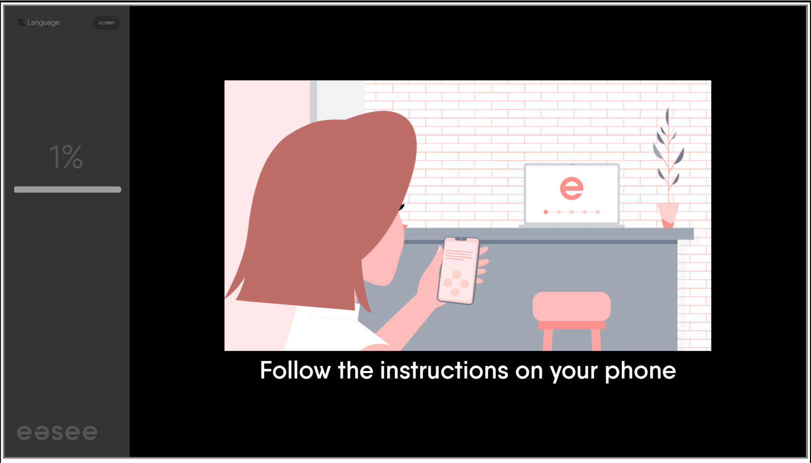
1%
Follow the instructions on your phone
easee

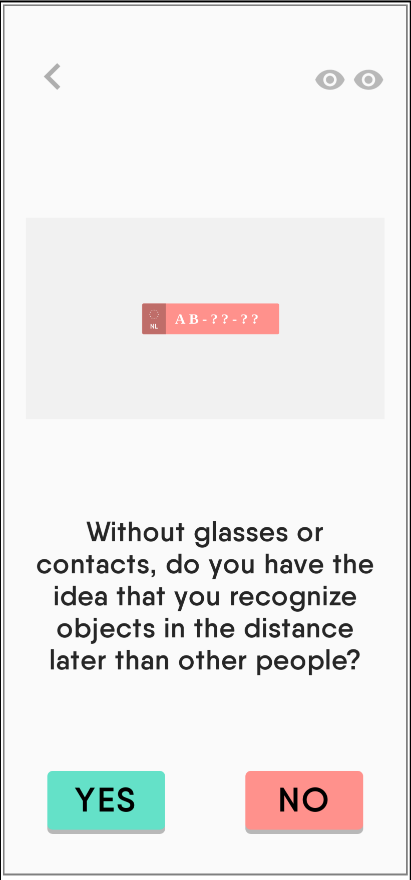
AB-??-??
Without glasses or contacts, do you have the idea that you recognize objects in the distance later than other people?
YES
NO

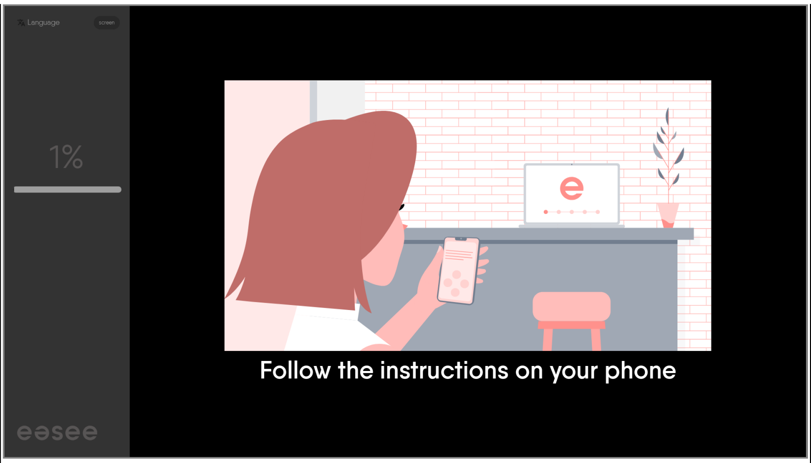
1%
Follow the instructions on your phone
easee

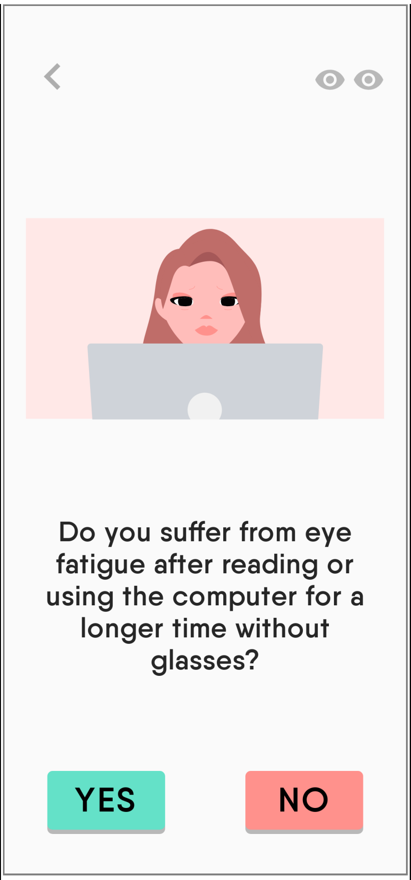
Do you suffer from eye fatigue after reading or using the computer for a longer time without glasses?
YES
NO

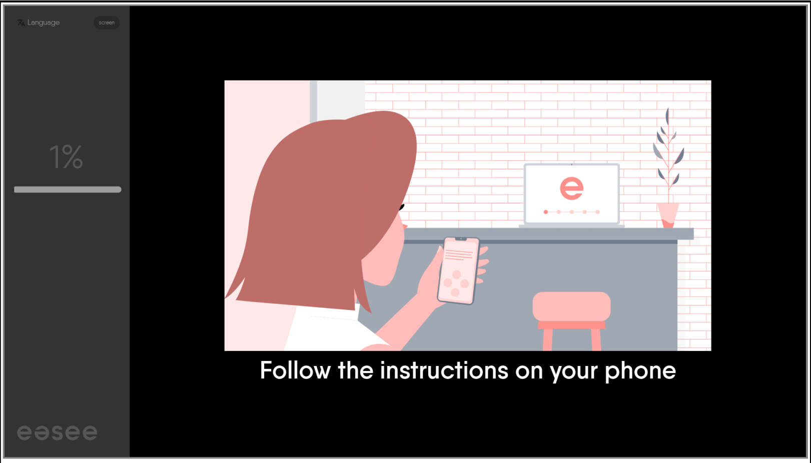
1%
Follow the instructions on your phone
easee

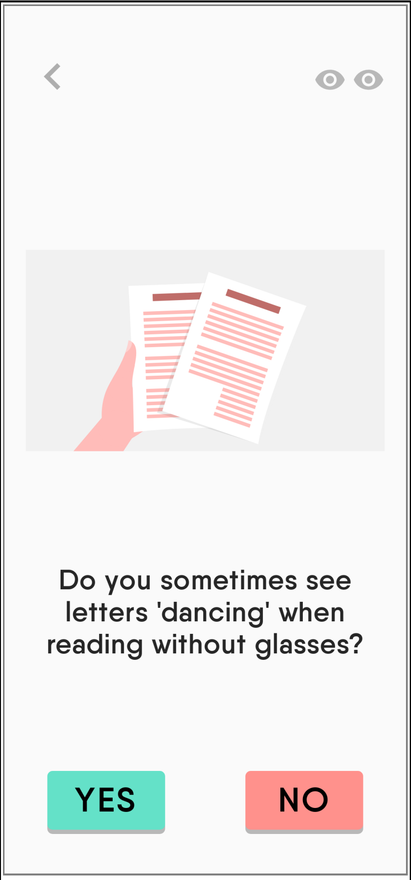
Do you sometimes see letters 'dancing' when reading without glasses?
YES
NO

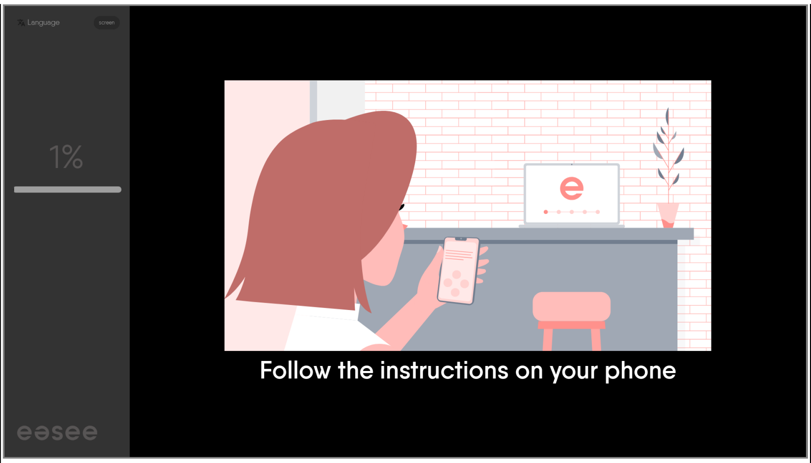
1%
easee
Follow the instructions on your phone

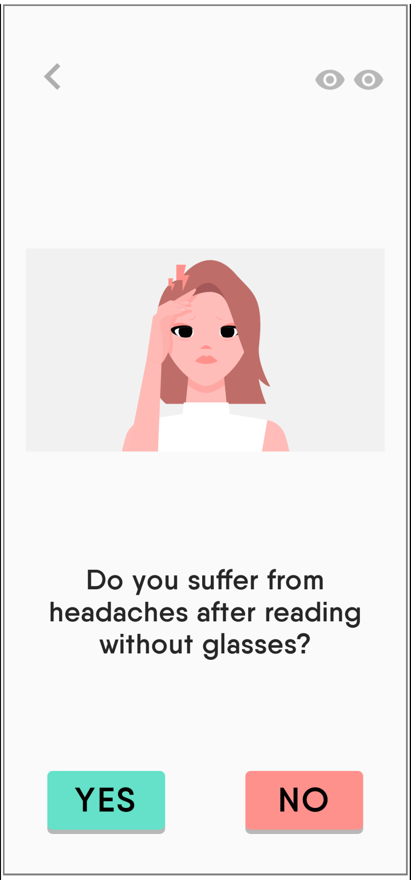
Do you suffer from headaches after reading without glasses?
YES
NO

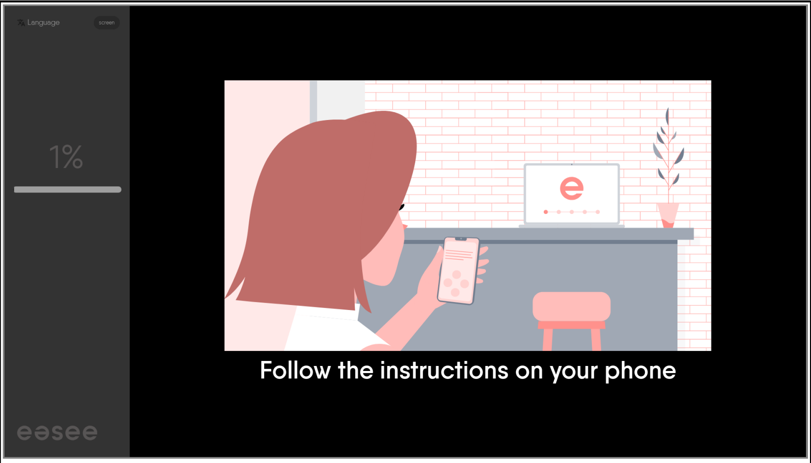
1%
easee
Follow the instructions on your phone

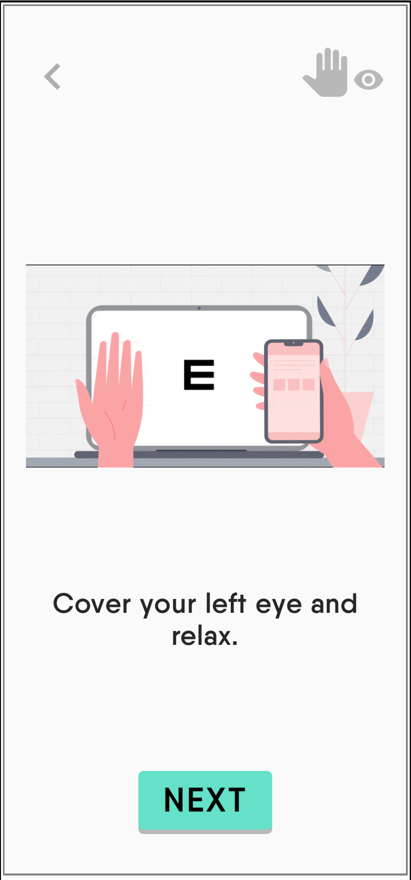
E
Cover your left eye and relax.
NEXT

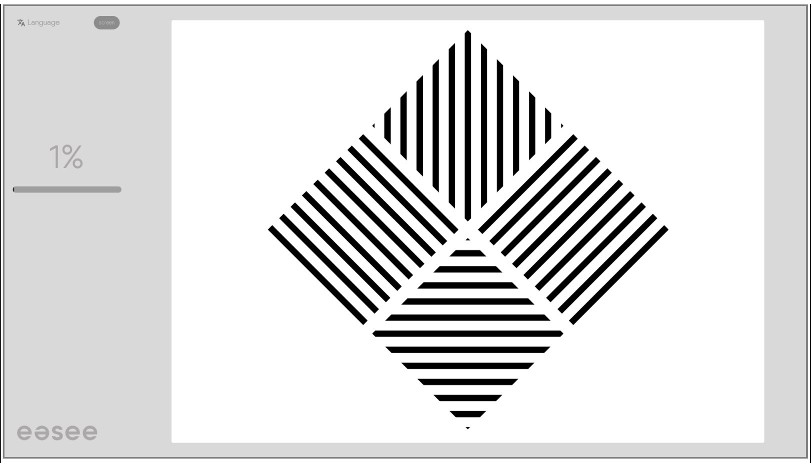
1%
easee

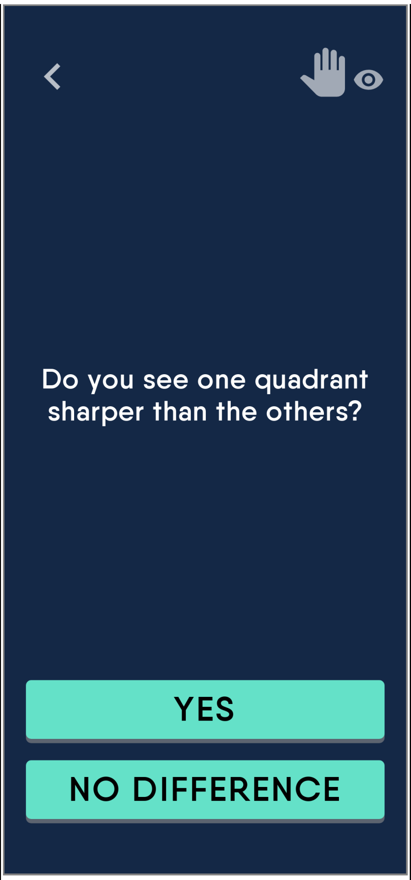
Do you see one quadrant sharper than the others?
YES
NO DIFFERENCE

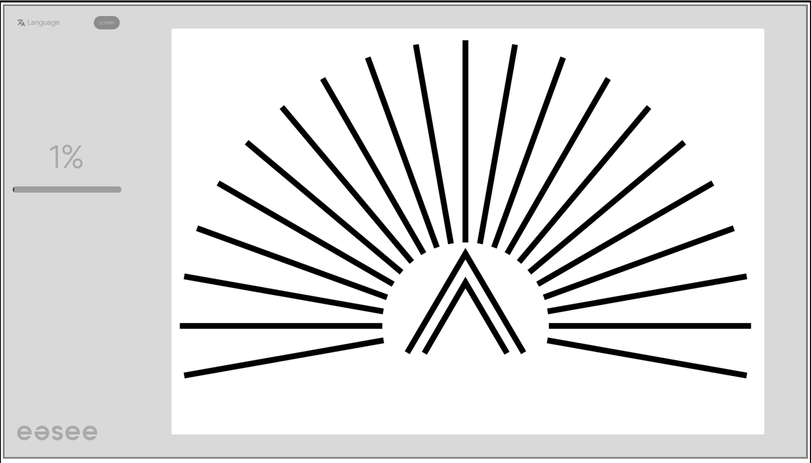
1%
easee

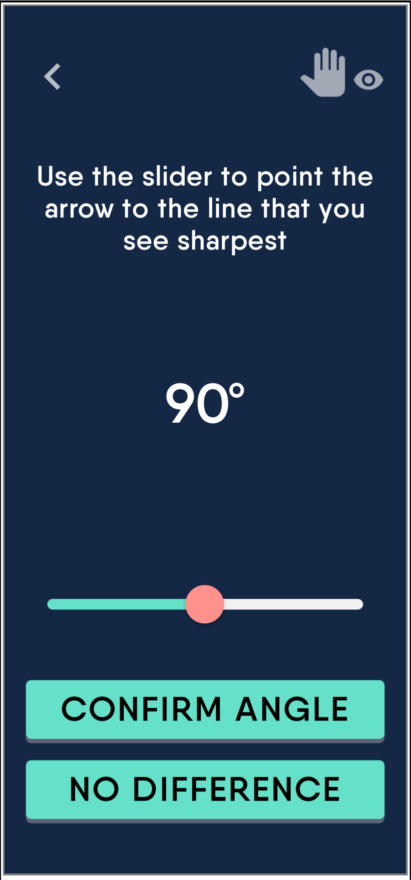
Use the slider to point the arrow to the line that you see sharpest
90°
CONFIRM ANGLE
NO DIFFERENCE

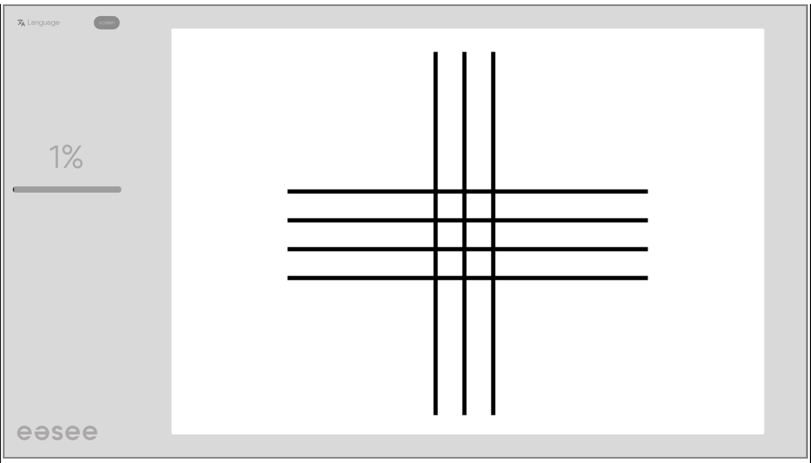
Language
1%
easee

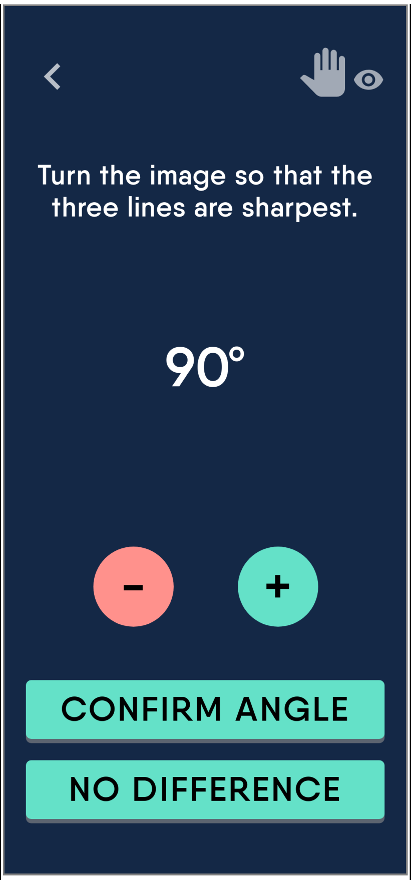
Turn the image so that the three lines are sharpest.
90°
-
+
CONFIRM ANGLE
NO DIFFERENCE

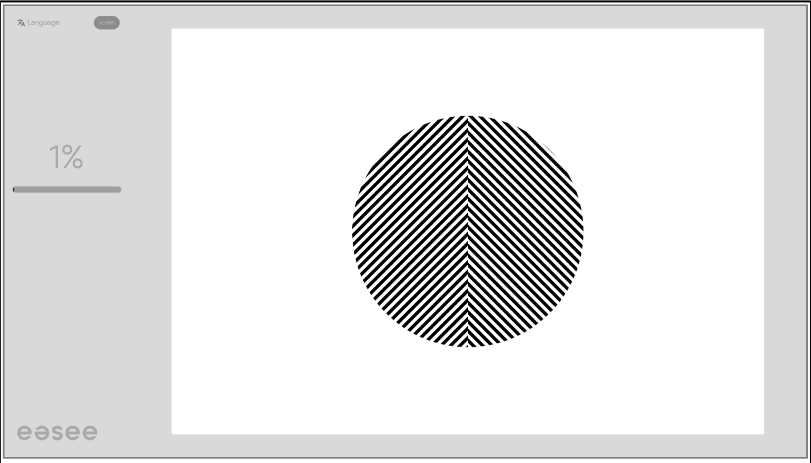
Language
1%
easee

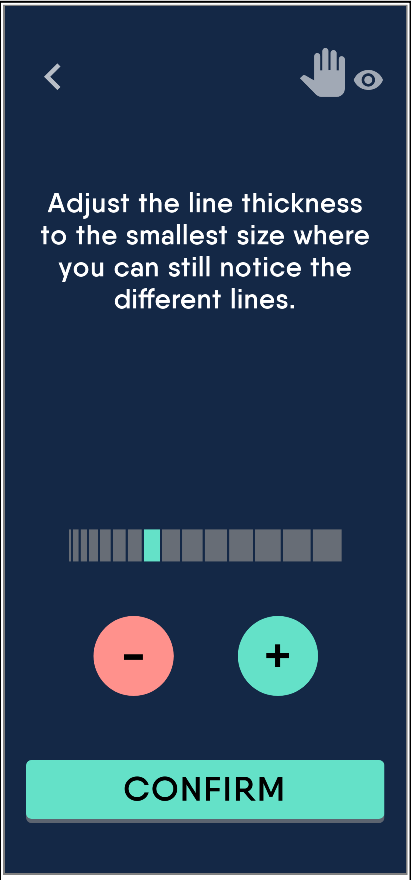
Adjust the line thickness to the smallest size where you can still notice the different lines.
-
+
CONFIRM

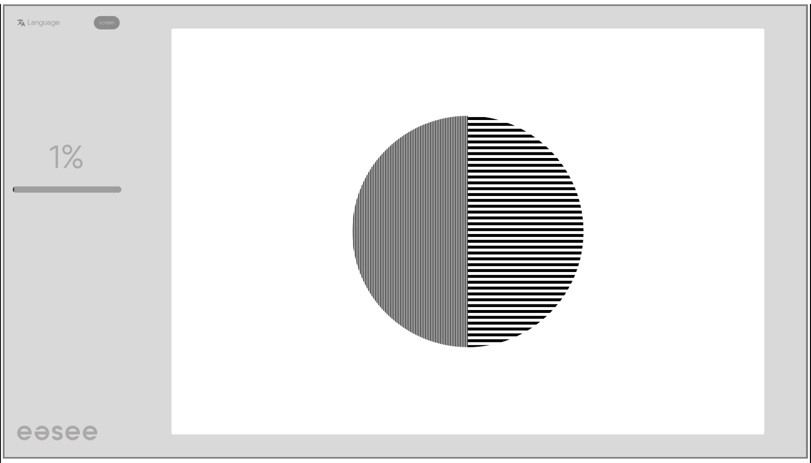
1%
easee

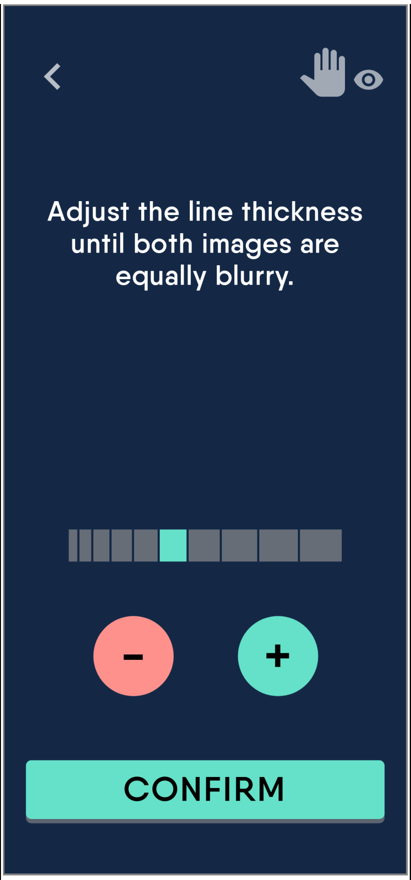
Adjust the line thickness until both images are equally blurry.
CONFIRM

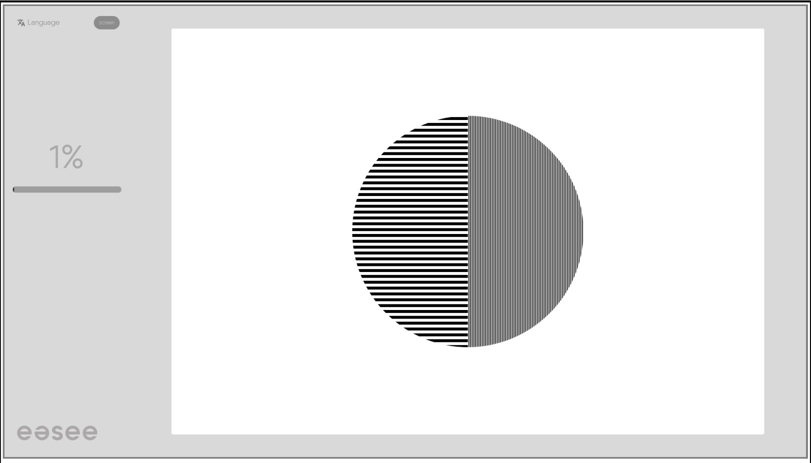
1%
easee

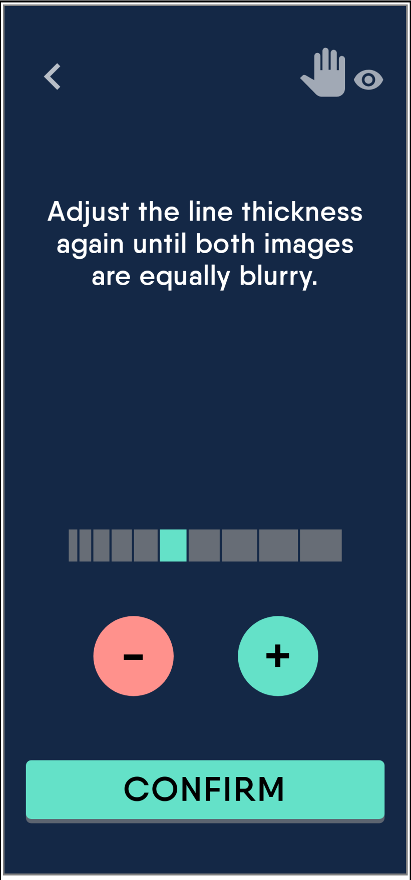
Adjust the line thickness again until both images are equally blurry.
CONFIRM

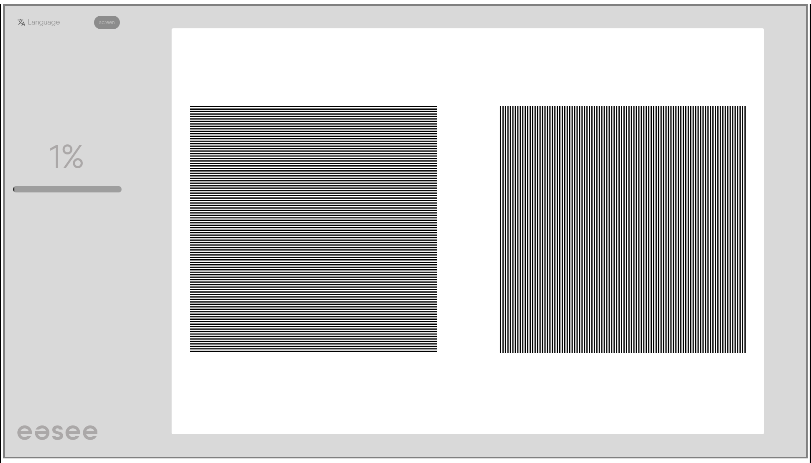
Language
1%
easee

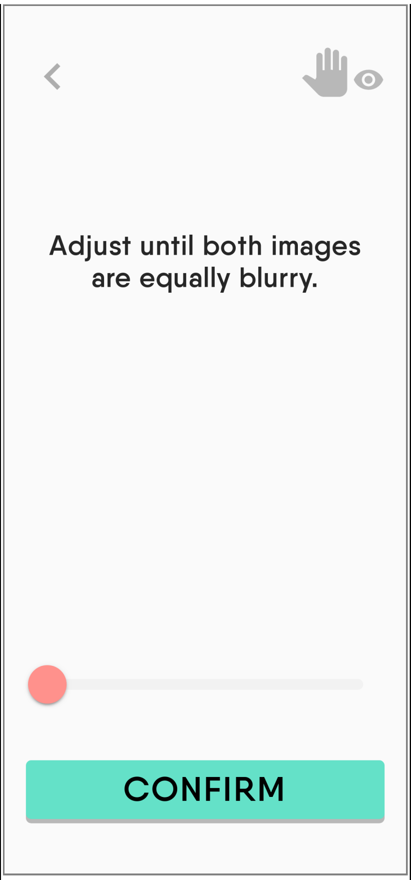
Adjust until both images are equally blurry.
CONFIRM

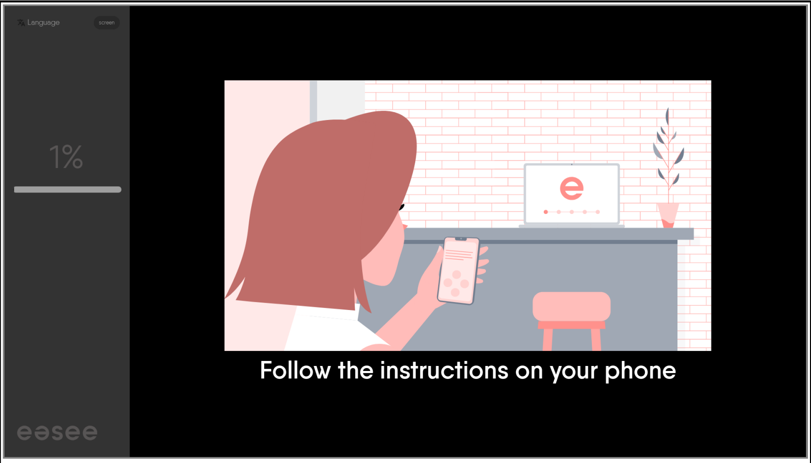
Language
1%
Follow the instructions on your phone
easee

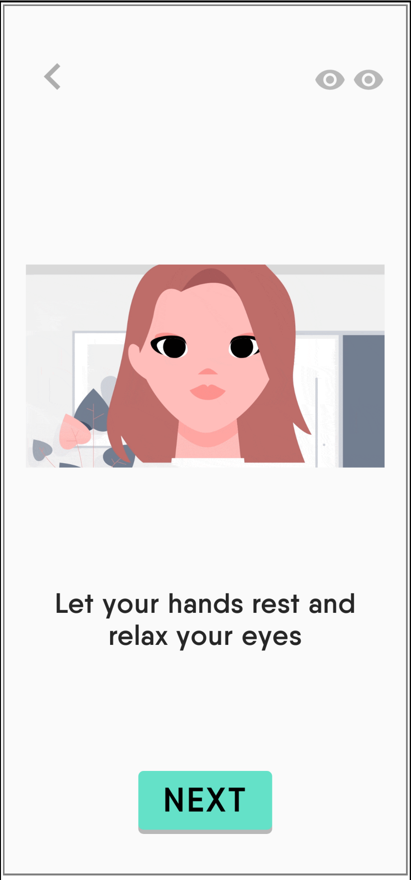
Let your hands rest and relax your eyes
NEXT

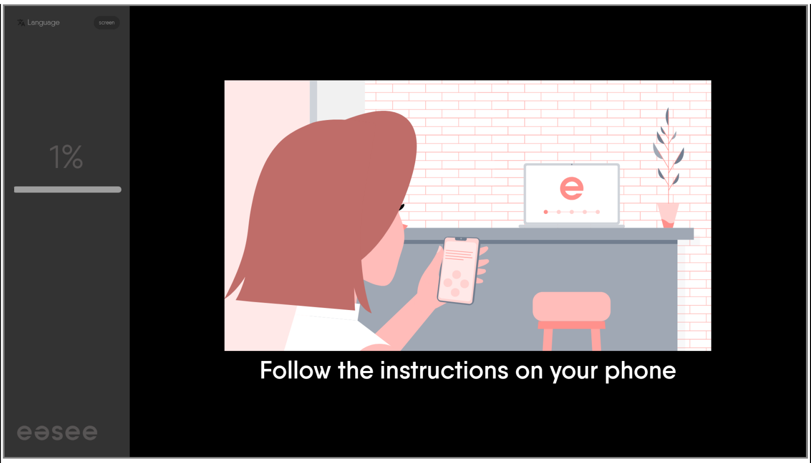
1%
easee
Follow the instructions on your phone

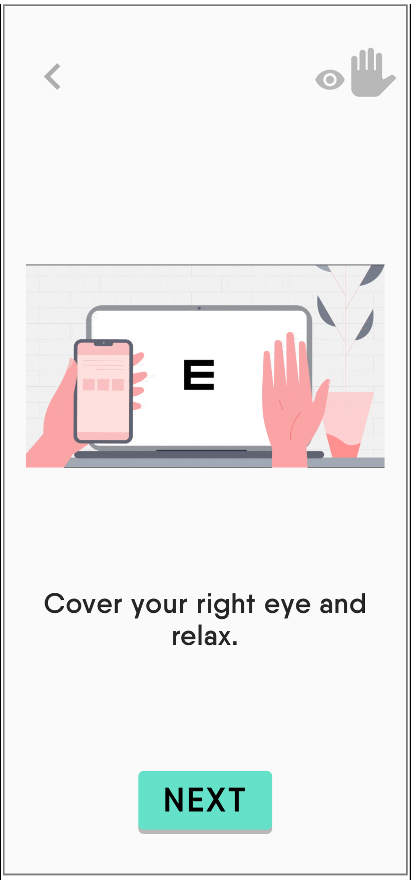
E
Cover your right eye and relax.
NEXT

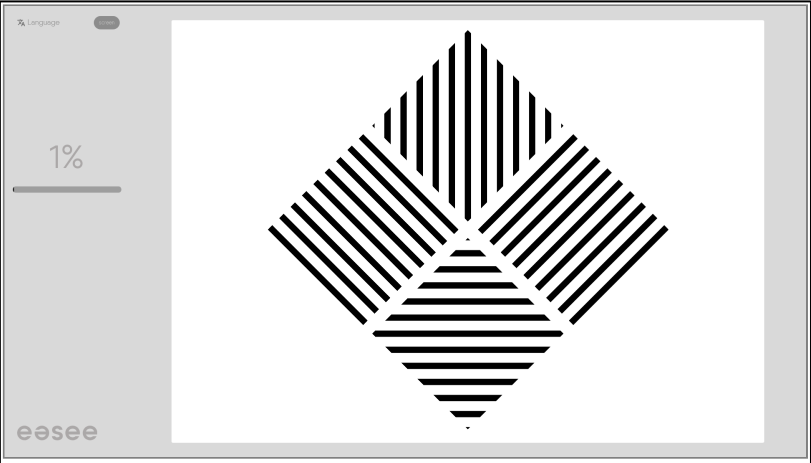
1%
easee

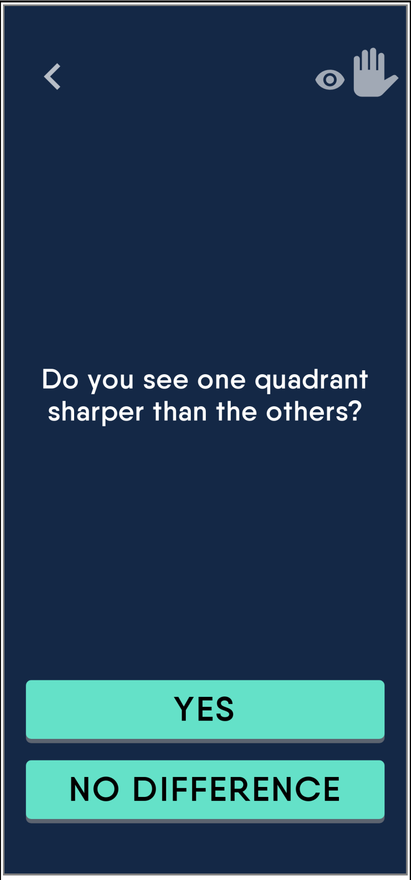
Do you see one quadrant sharper than the others?
YES
NO DIFFERENCE

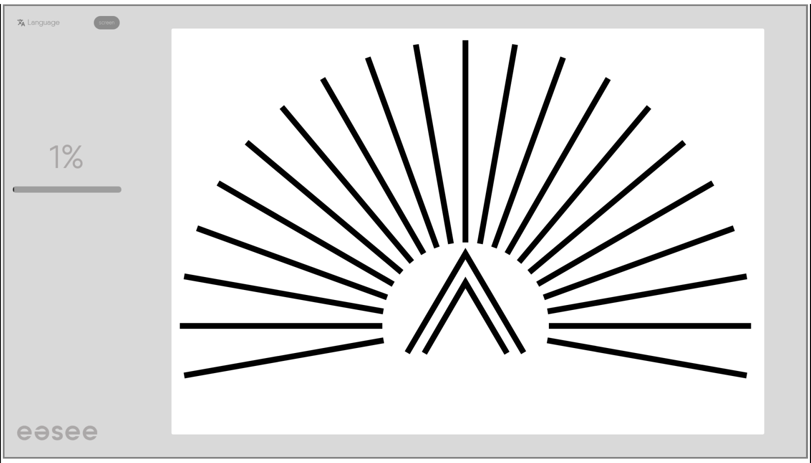
Language
1%

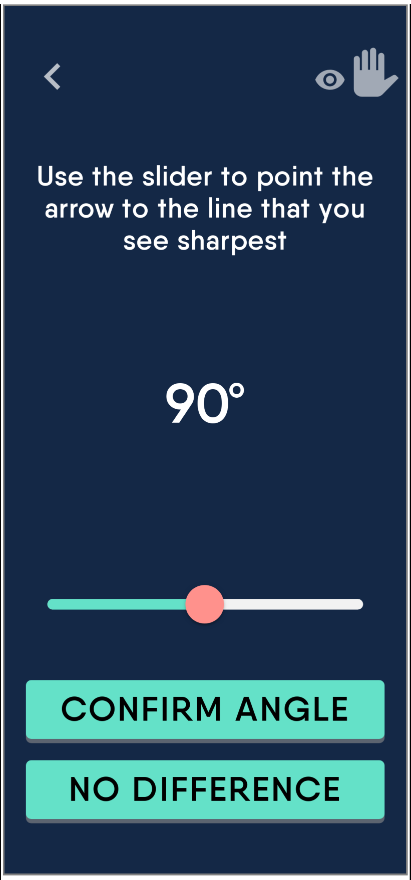
Use the slider to point the arrow to the line that you see sharpest
90°
CONFIRM ANGLE
NO DIFFERENCE

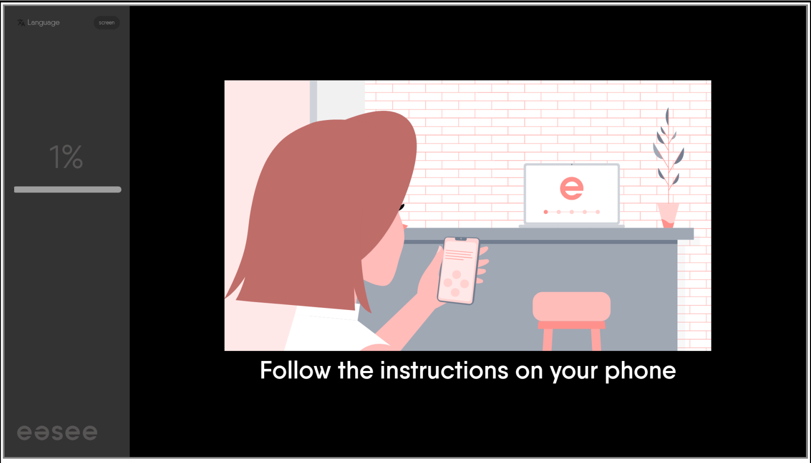
Language
1%
Follow the instructions on your phone

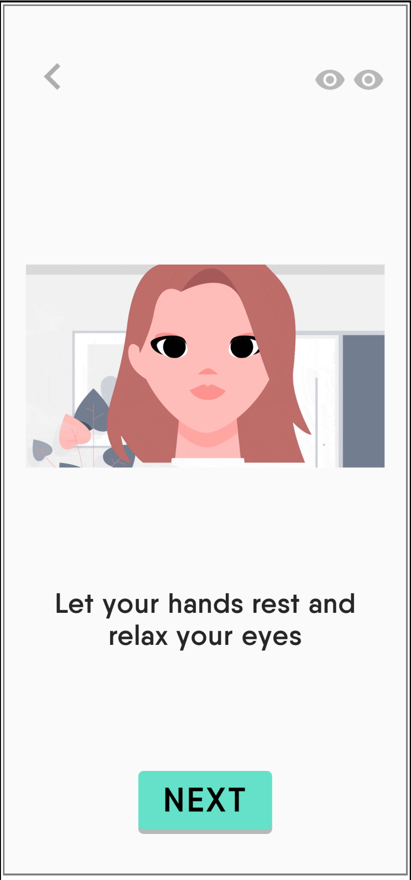
Let your hands rest and relax your eyes
NEXT

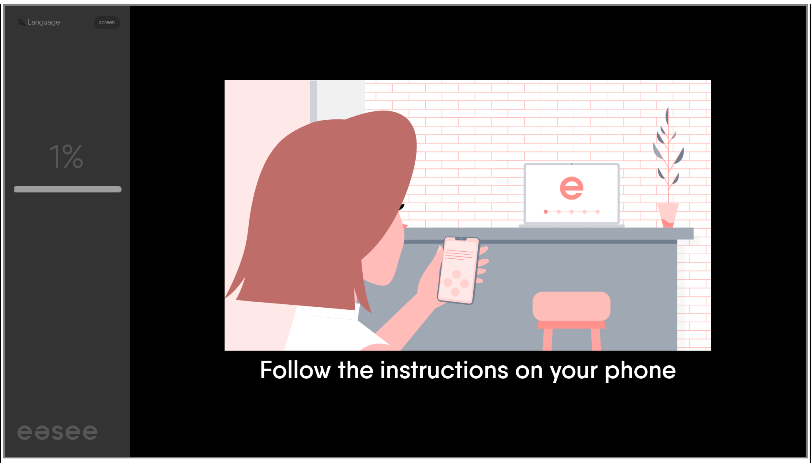
1%
Follow the instructions on your phone
easee

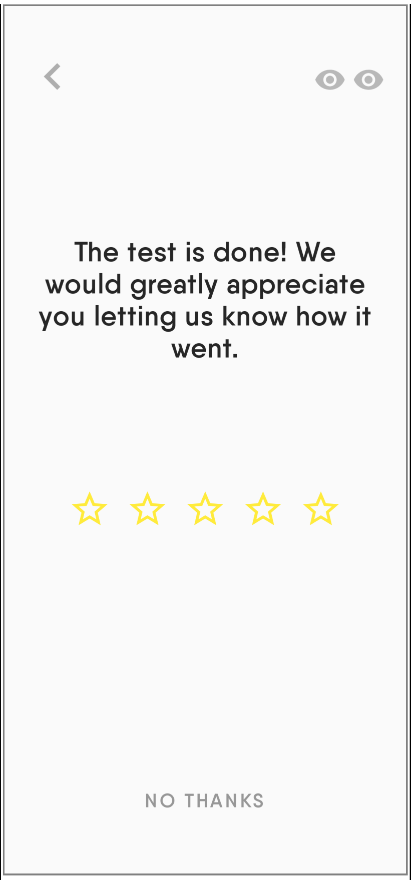
The test is done! We would greatly appreciate you letting us know how it went.
NO THANKS

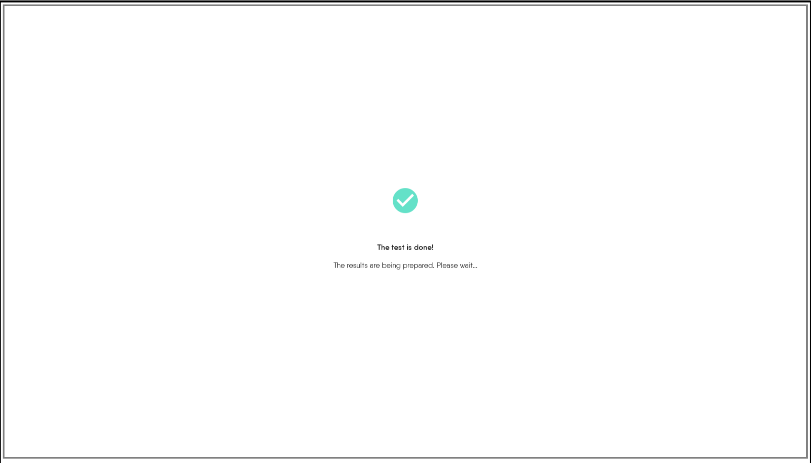
The test is done!
The results are being prepared. Please wait...

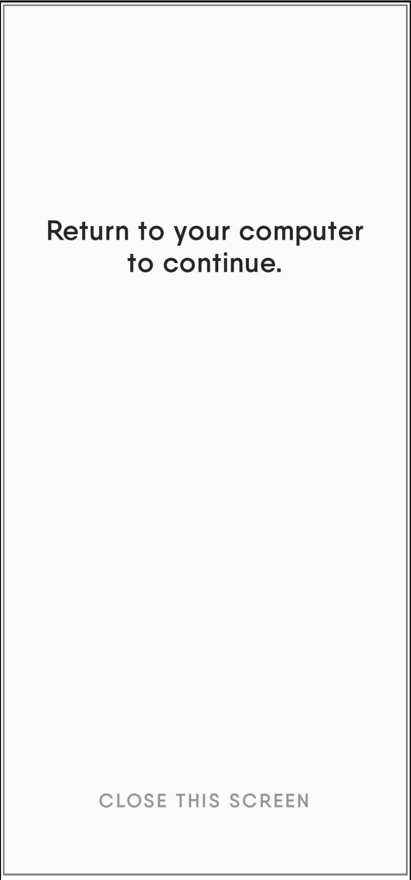
Return to your computer to continue.
CLOSE THIS SCREEN
