# Supplementary material for: Validation of an Independent Web-Based Tool for Measuring Visual Acuity and Refractive Error (the Manifest versus Online Refractive Evaluation Trial): Prospective Open-Label Noninferiority Clinical Trial
Source: J Med Internet Res. 2019 Nov 8;21(11):e14808. doi: 10.2196/14808 (PMC6874802; doi:10.2196/14808)
Supplement: Multimedia Appendix 4 [file jmir_v21i11e14808_app4.pdf]

Supplementary data. Refractive error and visual acuity measured in the myopic and hyperopic participants. (1st & 2nd generation algorithm combined)

	Manifest refraction[a]	Online refraction[a]	Diffe-rence	95% confidence interval		*P*-value[b]
				lower	upper	
**Myopic participants**						
Vector (D)[d]	1.70±1.60	1.58±1.30	0.12	-0.02	0.26	0.08
Spherical equivalent (D)	-1.65±-1.62	-1.47±1.31	-0.18	-0.30	-0.06	
Spherical power (D)	-1.43±1.55	-1.24±1.40	-0.19	-0.33	-0.06	
Cylindrical power (D)	-0.44±0.50	-0.47±1.05	0.02	-0.15	0.19	
Cylindrical axis (degrees)	97±57	85±54	12	5	28	
CDVA LogMAR[e]	-0.13±0.06	-0.02±0.18	-0.11	-0.14	-0.08	<0.01
CDVA Snellen[e]	1.37±0.19	1.12±0.35	0.25	0.20	0.30	
**Hyperopic participants**						
Vector (D)[d]	0.71±0.78	0.22±0.38	0.48	0.21	0.75	<0.01
Spherical equivalent (D)	0.66±0.78	0.02±0.41	0.63	0.31	0.95	
Spherical power (D)	0.84±0.89	0.10±0.43	0.73	0.38	1.09	
Cylindrical power (D)	-0.36±0.38	-0.16±0.27	-0.20	-0.33	-0.08	
Cylindrical axis (degrees)	85±64	66±38	19	-53	91	
CDVA LogMAR[e]	-0.13±0.06	-0.07±0.12	-0.06	-0.10	-0.02	<0.01
CDVA Snellen[e]	1.37±0.18	1.22±0.27	0.15	0.06	0.25	

CDVA, corrected distance visual acuity; LogMAR, logarithm of the minimum angle of resolution; Snellen, decimal visual acuity.

[a] Unless otherwise specified reported as mean ±standard deviation.

[b] Paired-sample Student's *t*-test was performed for predefined primary and secondary outcome parameters only.

[c] Generalized estimates equation model to statistically correct for the inclusion two eyes of one subject, age, and sex.

[d] Spherical and cylindrical power and axes were translated into vectors using Fourier analysis.

[e] Assessed with either the manifest or online achieved correction
